# Revisiting Target‐Aware *de novo* Molecular Generation with TarPass: Between Rational Design and Texas Sharpshooter

**DOI:** 10.1002/advs.75411

**Published:** 2026-04-22

**Authors:** Rui Qin, Zijie Chen, Yurong Li, Meijing Fang, Longji Shen, Yilong Su, Odin Zhang, Qinghan Wang, Qun Su, Jike Wang, Tingjun Hou, Yu Kang

**Affiliations:** ^1^ College of Pharmaceutical Sciences Zhejiang University Hangzhou Zhejiang China; ^2^ Zhejiang Provincial Key Laboratory for Intelligent Drug Discovery and Development Jinhua Zhejiang China; ^3^ College of Computer Science and Technology Zhejiang University Hangzhou Zhejiang China; ^4^ College of Pharmacy Guilin Medical University Guilin Guangxi China; ^5^ Department of Computer Science & Engineering The Chinese University of Hong Kong Hong Kong China

## Abstract

Target‐aware molecular generation models hold promise for drug discovery, but it remains unclear whether they genuinely exploit target information or merely resemble the Texas Sharpshooter fallacy by retrospectively rationalizing outputs. To address this, we introduce TarPass, a benchmark comprising a curated dataset of 18 well‐studied targets with expert‐annotated key interactions and experimentally validated active compounds, enabling fair evaluation of target‐aware *de novo* molecular generation models. We assessed 15 representative models across three paradigms: non‐3D, 3D in situ, and optimization‐based, considering protein‐ligand interactions (PLIs), molecular plausibility, and drug‐likeness. Results show that 3D in situ models have a modest average advantage in predicted PLIs. However, many fail to outperform random sampling. Non‐3D models, benefiting from broader pretraining, generate more drug‐like and synthesizable molecules but exhibit weaker target specificity. Optimization‐based methods effectively redirect outputs toward favorable chemical regions for single properties, often at the expense of others, for example by reducing compliance with Lipinski's rules. Integrating these insights, we propose a multi‐tier virtual screening workflow for target‐aware molecular generation as a post‐processing strategy to enrich molecules with improved PLIs and plausibility. Overall, this study highlights the limitations of current models in capturing fine‐grained target‐specific constraints and provides a standardized framework for future structure‐based drug design.

## Graphical abstract

TarPass provides a rigorous benchmark for target‐aware *de novo* molecular generation by jointly evaluating protein‐ligand interactions, molecular plausibility, and drug‐likeness on 18 well‐studied targets. Results show that current models often fail to consistently surpass random baseline in target‐specific enrichment, while post hoc multi‐tier virtual screening effectively improves candidate quality and practical utility.

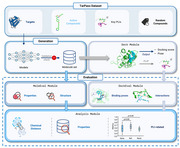

## 1 Introduction

Early stage drug discovery remains a costly and uncertain endeavor, as identifying bioactive compounds typically requires extensive screening of vast chemical libraries. In recent years, generative models have emerged as transformative tools to accelerate this process, enabling efficient navigation of chemical space and the proposal of novel molecules beyond enumerated databases [1]. Among these approaches, target‐aware methods occupy a unique position: rather than generating molecules through unconstrained exploration of chemical space or relying on high‐throughput screening, they explicitly integrate protein target information to guide molecular generation. This paradigm promises to enhance the biological relevance of generated candidates by embedding structural and functional constraints derived from the target, thereby narrowing the search toward compounds with a higher probability of forming meaningful protein‐ligand interactions (PLIs).

**FIGURE 1:**
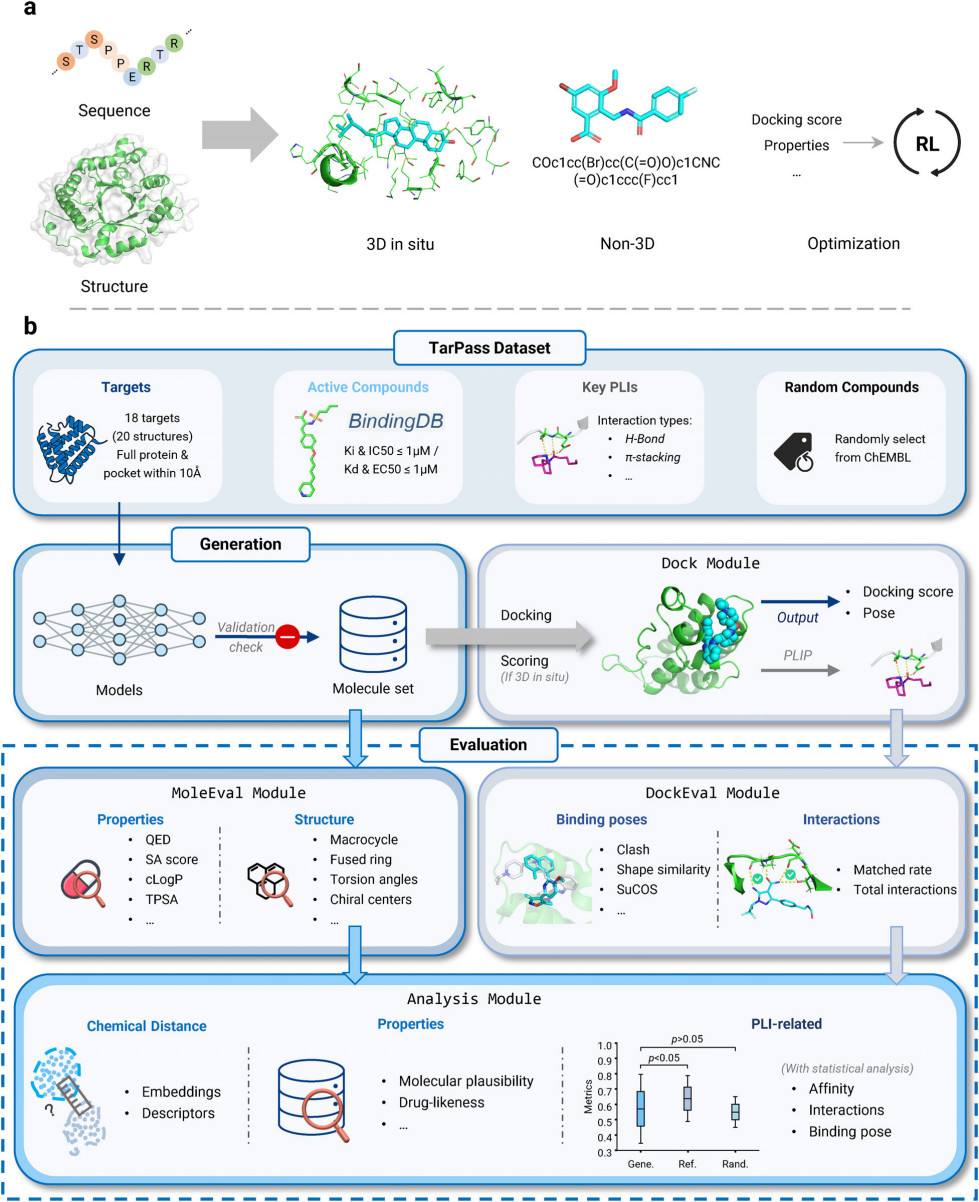
(a) Three paradigms of target‐aware *de novo* molecular generation. (b) Schematic overview of the TarPass benchmark workflow. Initially, the generated molecules are subjected to validation check and organized into sets that contain a sufficient number of unique molecules. Subsequently, these sets are passed to the docking module for primary docking. Following this, the molecule sets and the docking results are jointly directed to the evaluation stage, where relevant properties and metrics are computed by MoleEval and DockEval, respectively. Finally, the results are fed into the analysis module for a comprehensive assessment. Molecular structures were visualized using PyMOL (the PyMOL Molecular Graphics System, Version 3.1.0 Schrödinger, LLC, open‐source build).

Current methodologies for target‐aware molecular generation can be broadly categorized into three paradigms (Figure 1a). First, 3D in situ models, which constitute the mainstream, operate directly on protein pocket structures [2] by employing techniques such as diffusion [3], flow matching [4], or autoregressive atom placement [5] to construct ligands in 3D space. While these models explicitly encode spatial complementarity, they require high‐quality structural inputs and incur considerable computational cost, raising concerns about scalability and generalization [6]. Second, non‐3D models, which do not explicitly rely on structural geometry, leverage target structural information or alternative modalities, such as sequence embeddings or proxy objectives, to generate 2D molecular graphs, chemical strings [7], or self‐defined chemical blocks [8, 9, 10]. These approaches offer broad applicability and computational efficiency, yet their lack of structural grounding limits interpretability and may hinder the recovery of realistic binding modes [11]. Third, optimization‐based methods are built upon either 3D in situ or non‐3D generators by introducing post hoc objectives [12, 13], including reinforcement learning, surrogate affinity predictors [7], or preference‐alignment [4] strategies. While such techniques can steer sampling toward desired regions of chemical space [14], their reliance on imperfect scoring functions often complicates multi‐objective optimization [15] and risks overfitting. These challenges highlight the difficulty of distinguishing true target‐specific learning from artifacts induced by the optimization process.

Despite recent advances, a critical gap persists in assessing the utility of target‐aware generative models. An ideal model should integrate target‐specific information and learned priors to capture the biophysical principles governing protein‐ligand recognition, thereby generating chemically plausible drug‐like molecules that enable rational design in real‐world drug discovery. However, current evaluation metrics are often limited to statistical summaries of docking scores and drug‐likeness metrics on test sets, both of which are coarse surrogates for PLIs and molecular validity of generated outputs.

These assessments are often questioned as being overly dependent on cherry‐picked case studies, which retrospectively justify model capability and risk introducing the Texas Sharpshooter fallacy (drawing conclusions from selectively highlighted data). While it is impractical to experimentally validate all generated molecules, computational filtering followed by targeted validation of a representative subset is necessary. Nevertheless, if a model fails to demonstrate superior enrichment over an equally sized sample from existing molecular databases, the additional computational cost becomes difficult to justify. This highlights the urgent need for a benchmarking framework grounded in well‐studied targets independent of training data, evaluating models holistically across both PLIs and molecular plausibility, thereby providing a more robust measure of performance and practical applicability in drug discovery.

Upon revisiting existing benchmarks, we observe that they provide only partial coverage of these two critical dimensions. For PLIs, most benchmarks focus narrowly on docking scores while neglecting broader interaction patterns across diverse targets, as exemplified by Durian [16]. While CBGBench [17] attempted to address this issue, its analysis of interactions remained limited to structural similarity, offering limited interpretability from a medicinal chemistry perspective. Regarding molecular plausibility, benchmarks predominantly rely on classical drug‐likeness filters and set‐level metrics borrowed from 2D benchmarks such as GuacaMol [11] and MOSES [18]. Few benchmarks have systematically examined molecular plausibility at the 2D level while incorporating active compounds as reference standards. Several benchmarks, such as the work by Sanjrani et al. [19], have made progress toward this goal by incorporating real‐world test targets. However, the majority remain centered on the 3D in situ paradigm, whose results often suffer from serious issues in conformational plausibility [6, 20, 21]. While a few studies have acknowledged this limitation and incorporated non‐3D methods for comparison, such as POKMOL‐3D [20] and the work by Zheng et al. [22], their limited scope and representativeness hinder robust evaluation. Furthermore, the lack of standardized benchmarking protocols exacerbates uncertainty, as inconsistent practices make it difficult to distinguish true target‐specific learning from post hoc rationalization.

Here, we propose TarPass, a benchmark designed to enable fair and comprehensive evaluation of target‐aware *de novo* molecular generation models across all paradigms, simulating their deployment in real‐world drug discovery (Figure 1b). The benchmark dataset consists of 18 well‐studied targets, including major and pharmacologically relevant protein classes, carefully selected to minimize overlap with commonly used training sets. For each target, we curated key interactions and compiled an experimentally validated reference set of approximately 1000 active compounds. The evaluation focused on two core dimensions: PLIs and molecular plausibility, with the latter expanded to include drug‐likeness and chemical distance metrics. Using this benchmark and evaluation framework, we systematically assessed and analyzed 15 methods across all three paradigms, including four non‐3D approaches (DeepBlock [10], DRAGONFLY [23], SimpleSBDD [9], and TamGen [8]), eight 3D in situ approaches (DiffSBDD [2], DrugFlow by Schneuing et al. [4], IPDiff [24], Lingo3DMol [25], MolCraft [26], PocketFlow [27], SurfGen [5], and TargetDiff [3]), and five optimization‐based methods (DrugFlow‐PA [4], MolPilot [28], and REINVENT [13]). DrugFlow‐PA and MolPilot are the optimization variants of DrugFlow and MolCraft, respectively, whereas REINVENT is a non‐3D model optimized via reinforcement learning. Finally, we proposed a multi‐tier virtual screening workflow as a post‐processing protocol for all types of target‐aware molecular generation methods to effectively enrich molecules with improved PLIs and molecular plausibility, thereby enhancing their practical utility in real‐world drug discovery.

## 2 Results

### 2.1 Conceptual Framework of Target‐Aware Molecular Generation

To systematically analyze target‐aware *de novo* molecular generation, we begin by formalizing the training and inference processes into two complementary definitions. These definitions provide a foundation for evaluating whether models truly capture target–molecule relationships or simply rely on superficial correlations.

Definition 1 (training paradigm, Equation 1): during training, a model aims to learn the joint probability distribution of target‐molecule pairs:

(1) $$L\left(\phi \right)=E_{\left(m,\;T\right)}\left[L_{gen}\left(m,\;T;\phi \right)\right]$$

where L(ϕ) denotes the training objective, ϕ represents the model parameters, *L_gen_* is the model's loss function, *m* denotes the ground‐truth molecules, and *T* stands for target information.

Building on this definition, we propose Conjecture 1: molecules generated in a target‐aware manner should encode meaningful PLIs. These interactions span both explicit physical and chemical contacts (e.g., hydrogen bonds) and implicit properties reflected in binding affinity (e.g., docking scores). PLIs also highlight an important constraint in the similarity‐diversity trade‐off of molecular generation [29]: only diversity that preserves the ability to form key interactions, a potential prerequisite for activity, can be considered meaningful. This conjecture raises a critical question: although 3D in situ methods explicitly incorporate spatial relationships, do they sufficiently capture PLIs? Conversely, can non‐3D paradigms, despite lacking explicit structural constraints, implicitly reconstruct PLIs through their learned mappings?

Definition 2 (inference paradigm, Equation 2): during the inference phase, the model generates molecules by sampling from a conditional distribution over the chemical space:

(2) $$m^{*}∼p_{\phi }\left(R|\;T\right),\;m^{*}\subseteq S$$

Here, m* denotes a generated molecule, $p_{\phi }$ represents the parameterized probability distribution, *R* denotes the possible molecular representations, and *S* corresponds to the chemical space.

Building on this definition, we propose Conjecture 2: target‐aware generative models should constrain their outputs to biologically relevant and target‐specific subspaces within the chemical space. In other words, active molecules associated with a given target occupy specific regions of the chemical space, and meaningful generative models should recapitulate these constraints. Molecules outside these regions may exhibit diversity but often lack structural plausibility or drug‐like properties.

Together, these two definitions and conjectures highlight two fundamental dimensions for evaluating target‐aware molecular generation models: PLIs and molecular plausibility. These perspectives mirror the classical paradigms of structure‐based and ligand‐based drug design, respectively, providing a natural and robust foundation for model assessment in real‐world drug discovery scenarios.

### 2.2 Description of TarPass Benchmark

We constructed the TarPass benchmark, which consists of a carefully curated dataset and an end‐to‐end evaluation workflow. The dataset comprises time‐split target structures, each accompanied by expert‐curated key interaction annotations and experimentally validated reference active molecules. This dataset is specifically designed to minimize data leakage from commonly used training sets, enabling an assessment of generalization capabilities under realistic drug discovery constraints, particularly when targets are from highly druggable families. Additionally, it supports assessments of the overall distribution of generated molecules through explicitly quantified and statistically grounded PLIs and molecular property metrics. By standardizing the number of generated molecules, docking protocols, and target‐level aggregated reporting, our framework provides a comparable and extensible testbed for systematic characterization of performance across different paradigms under a unified protocol.

**FIGURE 2:**
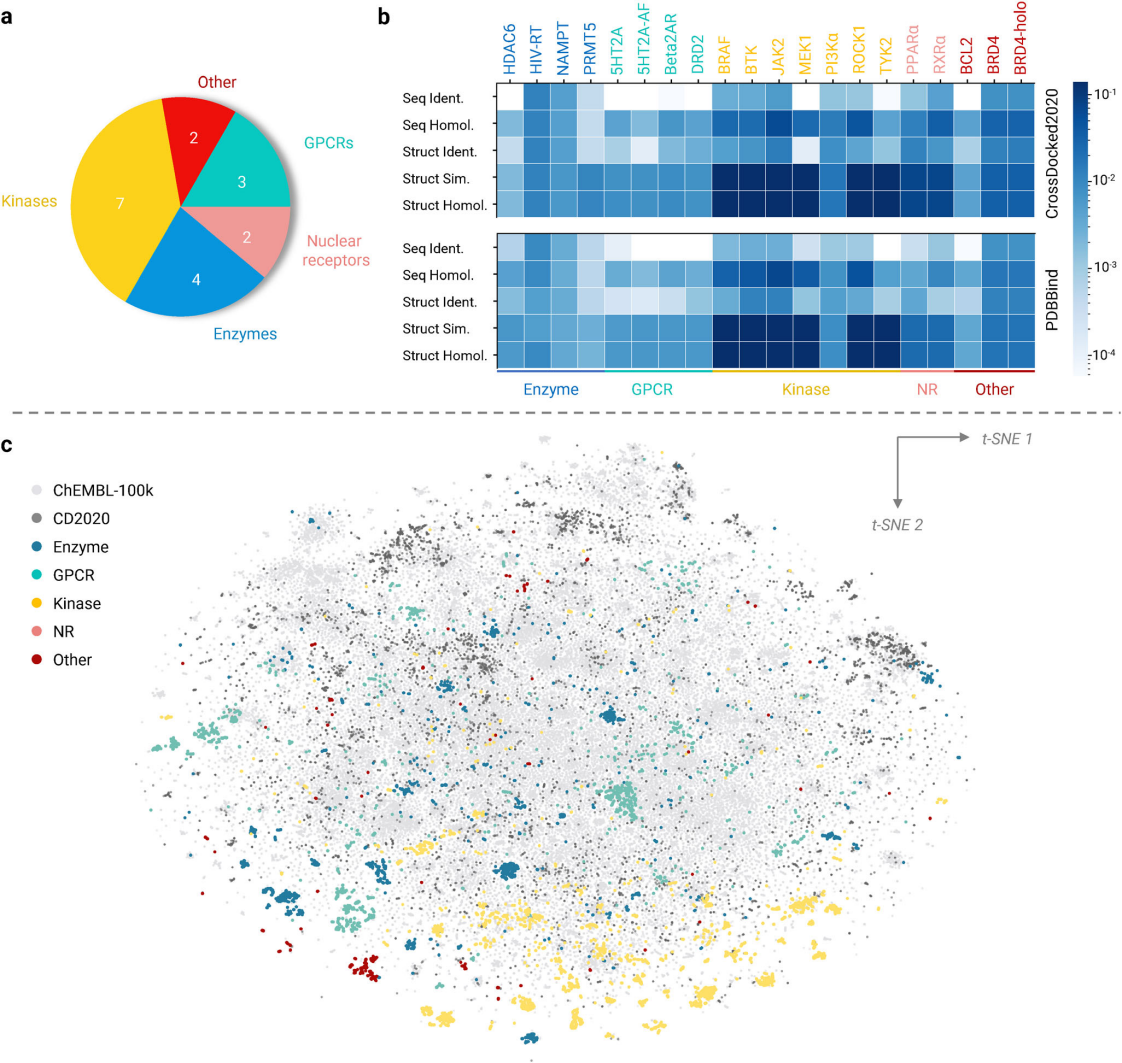
(a) A pie chart showing the classification of test targets by protein family. (b) Sequence and structural similarity analysis between the test targets and those in two commonly used training datasets, CrossDocked2020 (CD2020) and PDBBind. (c) A t‐SNE projection of the chemical space, using ChEMBL molecules as the background, illustrating the distribution of all reference active compounds alongside molecules from the CD2020 training set.

The target structure dataset was sourced from POKMOL‐3D [20] and curated to avoid direct overlap with commonly used structure‐ligand datasets such as CrossDocked2020 [30] and PDBbind [31]. Specifically, we constructed a subset of targets with release dates no earlier than December 2019 and further expanded its coverage by addressing two dimensions that are often underrepresented in existing benchmarks. First, considering that in real‐world drug discovery settings, some targets may only be available in their unbound (*apo*) experimental or computational forms, we incorporated ligand‐free structures for two targets (5‐HT2A and BRD4) as controls, enabling direct comparisons with their bound (*holo*) counterparts. Second, given that allosteric sites are typically not explicitly included in benchmarks, we added two additional allosteric targets (MEK1 and TYK2) to evaluate whether models can generate molecules for those noncanonical binding sites. The final dataset contains 20 structures for 18 targets, spanning kinases, non‐kinase enzymes, GPCRs, nuclear receptors, and two other proteins (Figure 2a, Table S1). Notably, all targets in the dataset are associated either with approved drugs or clinical candidates, ensuring biomedical relevance and alignment with practical constraints in drug discovery.

To quantify the extent of potential data leakage and the inherent challenges of generalization, we compared the test targets against those in CrossDocked2020 and PDBbind using sequence‐ and structure‐based searches with MMseqs2 [32] and Foldseek [33] (Figure 2b). The results reveal that all test targets show <1% sequence identity with entries in these training sets, showing limited direct overlap at the sequence level. Unsurprisingly, structural similarity is higher among kinases across the set (often >10%), reflecting the realistic setting that druggable protein families are heavily represented in public datasets. Consequently, we explicitly framed this benchmark to evaluate generalization within representative protein families, rather than relying on unrealistically “novel‐family” targets.

Key conserved interactions were curated to provide mechanistically grounded evaluation targets, defined based on three stringent criteria: (1) conserved residues and binding features implicated in endogenous ligand recognition supported by the literature; (2) interactions supported by crystallographic evidence or mutagenesis experiments; and (3) recurrent contacts observed across multiple structures identified with PLIP [34]. Most of these interactions consist of hydrogen bonds and hydrophobic contacts, along with salt bridges, π‐stacking, and halogen bonds. Water bridges and metal‐ligand interactions were not considered. The details on targets and interactions are provided in the Supporting Information section.

For each target, experimentally validated active compounds were collected from BindingDB [35] to form target‐specific reference sets. Following dimensionality reduction using t‐SNE [36] (Figure S1), the distribution of these active compounds exhibited distinct clustering patterns by target and protein family, supporting the previous conjecture that active molecules corresponding to a target are inherently constrained within specific regions of chemical space. Compared to randomly selected molecules from the ChEMBL35 [37] dataset (Figure 2c), the actives were broadly dispersed across drug‐like chemical space, whereas molecules from CrossDocked2020 occupied a relatively narrower region, highlighting potential coverage bias.

In addition, a set of 1000 molecules randomly sampled from ChEMBL was included as a baseline. These molecules exhibited a wide range of heavy atom counts (4 to 54, with a mean of 28.40) and QED values (0.04 to 0.95, with a mean of 0.56), reflecting substantial chemical diversity. Notably, as the molecules in ChEMBL are largely enriched for bioactivity, two compounds in the random baseline overlap with reference compounds for specific targets. Although this baseline is not purely noise, it effectively simulates the practical scenario of identifying potential active compounds via virtual screening within a known, drug‐like chemical space. This baseline enables assessment of whether a model provides value beyond random sampling from existing chemical databases, complements target‐specific reference comparisons, and prevents over‐interpretation of absolute docking scores.

We established a unified and transparent benchmark workflow (Figure 1b) to ensure consistent and comparability across models. For each target, the evaluated model is run for up to two rounds of generation to produce 1000 unique molecules, striking a balance between molecular diversity and generation efficiency (Figure S2). Subsequently, the generated molecules are processed through a standardized docking module, which closely mirrors common practice in real‐world drug discovery applications. For 3D in situ methods, an additional rescoring stage is implemented to evaluate the initial conformations of the generated molecules. The docking scores, poses, and interactions obtained from this module are then passed on to the evaluation phase, along with the sets of generated molecules. The evaluation and analysis framework consists of three key modules: (1) assessment of molecular plausibility, (2) analysis of PLIs, and (3) an integrated analysis of the first two modules at the target level. All results are exported in standardized formats, enabling systematic comparison and downstream meta‐analysis across different models.

### 2.3 Basic Performances of Generation

We first evaluated the practical deployability of various target‐aware molecular generation methods within a unified benchmark framework, focusing on three key aspects: runtime efficiency, compatibility with input structures, and quality of set‐level outputs (Table 1).

**TABLE 1:** Overview of the evaluated models and their basic performance.

Paradigm	Model	Training set	Failed/ Substandard	Val	Uniq@1k	Time (s/mol)	Note
Non‐3D	DeepBlock [10]	ChEMBL31 + CD2020		1	1	0.058_±0.018_	Building‐block based
	DRAGONFLY [23]	ChEMBL29 + PDBBind2020		1	1	0.071_±0.004_	Interactome data
	SimpleSBDD [9]	ZINC 250k + CD2020	1(F)	0.966	0.987	0.021_±0.001_	
	TamGen [8]	PubChem + CD2020	1(F)	∼1	0.968	0.030_±0.001_	
3D in situ	DiffSBDD [2]	CD2020	1(S)	0.968	0.987	1.127_±0.328_	
	DrugFlow [4]	CD2020		0.831	0.399	1.772_±0.235_	
	IPDiff [24]	CD2020 & PDBBind2016		∼1	0.940	15.617_±3.575_	Interaction prior
	Lingo3DMol [25]	In‐house + DUD‐E & PDBBind		∼1	0.906	8.563_±6.829_	Interaction prior
	MolCraft [26]	CD2020		1	0.498	2.475_±0.395_	
	PocketFlow [27]	ZINC + CD2020		1	0.692	1.813_±0.183_	Chemical knowledge constraints
	SurfGen [5]	CD2020	1(S)	1	0.948	51.051_±70.671_	
	TargetDiff [3]	CD2020		∼1	0.968	16.664_±9.001_	
Optimization	DrugFlow‐PA [4]		1(S)	0.600	0.637	‐^#^	Property optimization based on DrugFlow
	MolPilot [28]		18(S)	1	1	4.230_±3.727_	VLB‐optimal schedule based on MolCraft
	REINVENT [13]	ChEMBL25		1	1	0.011_±0.001_	

**Training set**: if a plus sign is present, it indicates that the model was pre‐trained on the dataset preceding the plus sign. **Failed/Substandard**: failed to generate molecules (F) / unable to generate molecules at the required standard (S); **Val**: validity; **Uniq@1k**: uniqueness when sampling up to 1000 non‐duplicate molecules. If fewer than 1000 unique molecules are obtained, uniqueness is computed based on the available set. **#**: Since DrugFlow‐PA shares an identical model framework to DrugFlow, no additional speed measurements were conducted. **Abbreviations**: CD2020: CrossDocked2020; VLB: Variational lower bound.

Significant differences in generation throughput were observed across different paradigms. Non‐3D models, such as TamGen, demonstrated relatively fast generation speeds, typically producing a molecule in less than 0.1 s. In contrast, 3D in situ approaches were substantially slower. Specifically, for 3D in situ models, flow‐based approaches (e.g., MolCraft, PocketFlow) required 1–2 s per molecule, while diffusion (e.g., IPDiff) or autoregressive models (e.g., SurfGen) often exceeded 10 s under our testing conditions. It is important to note that the reported timing is measured as the average wall‐clock time from code initiation to the generation of readable molecular files. Although we employed the same hardware to minimize the impact of model initialization overhead, the contribution of post‐processing steps cannot be overlooked. For instance, models like TargetDiff apply additional post‐processing steps to refine their generated outputs, which affects overall runtime.

Next, we assessed whether each method can be executed on the provided structural inputs without requiring model‐specific adaptation. Two models exhibited incompatibilities on specific targets: SimpleSBDD was unable to process the zinc ion present in HDAC6, as well as other metal ions due to the absence of metal atoms in its predefined atom vocabulary, indicating limited support for metal‐coordination environments. Additionally, TamGen does not accept user‐defined structures, so the AlphaFold‐predicted structure of 5‐HT2A was not accepted as input.

At the molecular‐set level, we evaluated whether each model can meet the generation quota across targets, as well as the proportions of valid molecules and non‐duplicate unique molecules produced. DiffSBDD, SurfGen, and DrugFlow‐PA fell short of the required number of unique molecules for only one target, whereas MolPilot failed to meet this criterion for 18 targets. Interestingly, MolPilot achieved a perfect uniqueness score of 1, which is consistent with its VLB‐optimal schedule that employs staged sampling along a “twisted” probability path coupling 2D topology and 3D conformation, thereby reducing repeated revisits to high‐probability 2D graphs and effectively suppressing duplicates. However, under a fixed iteration budget, MolPilot's limited yield of valid samples can hinder its ability to reach the required set size.

In terms of validity, all models outside the DrugFlow series achieved high validity. When invalid outputs occurred, they were dominated by chemically implausible Kekulé forms, suggesting that bond assignment in the generated molecular graphs remains a key failure mode. However, it should be noted that models with post‐processing, as mentioned above, may filter out invalid molecules, thereby artificially inflating their apparent performance.

Regarding uniqueness, non‐3D methods consistently produced highly unique sets. Among 3D in situ approaches, models excluding flow‐based variants also achieved competitive uniqueness, typically higher than 0.9. In contrast, flow‐based models such as DrugFlow and PocketFlow generally exhibited uniqueness scores below 0.7, suggesting potential issues in their sampling schemes over molecular graphs.

### 2.4 Analysis of Protein‐Ligand Interactions

#### 2.4.1 Docking Performance

Docking is commonly used as a rough surrogate metric for evaluating the binding affinity between targets and molecules. Here, docking scores and interaction metrics should be interpreted as outputs of a standardized computational proxy rather than direct estimates of experimental binding affinity, as well as errors in docking may propagate into downstream target‐level and cross‐model comparisons.

**FIGURE 3:**
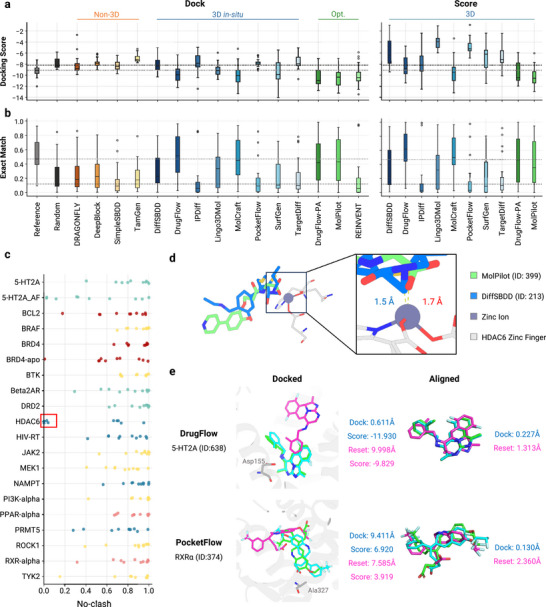
Performance and distribution of the evaluated models or methods in terms of (a) Vina Score (Ref. median = −9.109 in gray dash and Rand. median = −8.217 in black dash) and (b) interaction recovery characterized by exact match rate (Ref. median = 0.470 and Rand. median = 0.121). (c) Distribution of no‐clash rates for the initial poses of 3D in situ models and optimized variants across targets. The red box highlights the unusually high number of clashes observed for HDAC6. (d) Steric clashes between generated molecules and the zinc finger domain of HDAC6. (e) Representative cases selected from the models for conformation‐reset docking, including docked and aligned conformations, together with their RMSD relative to the initial poses.

Our analysis, across redocking and rescoring (Figure 3a,b; Table S2), revealed that 3D in situ methods show a clear average advantage over non‐3D paradigms in terms of docking scores. However, only a small subset of these methods demonstrated statistically significant improvements over random baselines. It is worth noting that, due to the inherent bias of ChEMBL toward bioactive chemical space, the baseline performance may be somewhat overestimated. Nevertheless, these observations suggest that optimizing docking scores may remain a challenging aspect for current target‐based generative models.

Redocking results demonstrated that 3D in situ models consistently achieve better docking scores than non‐3D paradigms, with mean median docking scores ranging from −7.5 to −10, which further improved to below −10 after optimization. However, statistical comparison against reference and randomly selected molecules showed that only one non‐3D, one optimization‐based model, and four 3D in situ models significantly outperformed random molecules in docking scores across more than 50% of targets, implying that the performance of many other models may not even surpass that of randomly selected molecules. Among the 3D in situ models, despite their generally better performance in redocking, only MolCraft and DrugFlow consistently outperformed non‐3D methods. The rescoring results further reinforced this trend: only these two models achieved median scores below −7.5, while lower‐performing models such as DiffSBDD even yielded positive scores.

In contrast to docking scores, most models exhibited substantially higher ligand efficiency compared to both random and reference molecules, primarily attributable to their lower molecular weights (Table S3). Considering the bias introduced by molecular weight on docking scores [38], this observation suggests that although models such as Lingo3DMol did not achieve better docking scores, they still partially captured the binding pocket and formed favorable interactions with a limited number of atoms. However, the spatial utilization of the binding site remains insufficient, indicating that improving this aspect could be an effective direction for further enhancing model performance.

#### 2.4.2 Interaction Recovery

Beyond evaluating binding affinity, the ability to accurately reproduce designated key interactions provides a more stringent test of a model's target awareness and reveals significant disparities between current generative models and reference ligands. Our evaluation of interaction recovery focused on two key metrics: the exact match rate (Exact Match), which measures the proportion of all specified interactions precisely reproduced, and the more relaxed match rate (Match Ratio), which accounts for the reproduction of any single designated interaction.

As shown in Table S4, reference ligands reached only a 51.4% exact match rate due to limitations in accuracy under the reliability of the current docking protocol, while the strict geometric and distance constraints imposed by PLIP may further introduce false negatives due to minor coordinate deviations. Nevertheless, this was still more than twice that of the random baseline (22.3%), thereby supporting the rationale for evaluating models based on key interactions. Under redocking conditions, most models performed no better than random molecules. Notable exceptions included DrugFlow and MolCraft, along with their optimized variants, which demonstrated performance comparable to that of reference ligands. Models incorporating explicit interaction priors showed divergent outcomes: IPDiff performed below the random baseline, whereas Lingo3DMol outperformed several 3D in situ approaches, suggesting that the design of interaction priors critically determines their effectiveness. REINVENT dropped below the random baselines, which likely resulted from its reinforcement learning procedure focusing solely on affinity as the reward.

Overall, accurate reproduction of key molecular interactions proved even more challenging than achieving favorable affinity, with only a few selected models approaching the performance level of reference ligands. Although docking accuracy and potential false negatives introduced by PLIP may lead to underestimation of the recovery, the relative trends remain consistent and support the conclusions above. In both docking scores and interaction metrics, the 3D in situ paradigm showed slightly superior average performance compared to the non‐3D paradigm, with DrugFlow and MolCraft standing out in particular. However, these two models require additional ligand input and extensive sampling. To examine this, we conducted further tests (Figures S3 and S4), revealing that their strong performance might be highly correlated with the original input ligands. This casts doubt on the robustness and generalizability of these two models.

#### 2.4.3 Initial Conformation and Docking Pose

Pose‐level analyses reveal that steric feasibility and binding mode fidelity continue to be significant limitations for most models: initial conformations generated by 3D in situ methods frequently contain clashes, and only a small subset of models achieves reference‐like binding‐mode similarity and sub‐ångström centroid accuracy, consistent with their superior interaction and affinity metrics.

Assessment of steric clashes using criteria from the PoseBusters suite [39] (Table S5) showed that initial poses from most 3D in situ models contained a certain proportion of clashes. With the exception of IPDiff, no method achieved a no‐clash rate above 85%. Optimization methods improved performance: DrugFlow‐PA and MolPilot increased their no‐clash rates by ∼13% and ∼8%, respectively, compared to their base models, with both exceeding 90%.

Structural analysis uncovered target‐specific factors contributing to steric conflicts. For 5‐HT2A_AF and HDAC6, nearly half of the models yielded a no‐clash rate below 0.5 (Figure 3c). In the case of 5‐HT2A_AF, the primary issue stemmed from models such as TargetDiff using an incomplete pocket with a 10 Å cutoff, which leads to clashes with the ECL2 region in the full binding pocket. For HDAC6, clashes primarily originated from the zinc finger domain, where most models either ignored the zinc ion or failed to capture zinc‐ligand coordination (Figure 3d). For example, DiffSBDD produced poses where the closest atom to the zinc ion was a carbon positioned at 1.5 Å, incompatible with the coordination requirement. Similarly, MolPilot generated an oxygen at 1.7 Å, both shorter than the typical Zn‐O bond length observed in crystal structures (>2 Å) [40].

The analysis of binding mode similarity further highlighted systematic trends. Using the co‐crystallized ligands from the target structures as references, we computed SuCOS, which combines shape overlap and chemical feature overlap of docking poses [41], and decomposed it into shape similarity and electrostatic similarity (ESP‐Sim) [42]. Reference ligands consistently outperformed random baselines across all metrics, confirming their validity. Overall, the patterns of binding mode similarity aligned with the affinity and interaction results: non‐3D models and certain 3D in situ models, such as IPDiff and PocketFlow, performed close to random baselines, while DrugFlow and MolCraft achieved similarity values comparable to those of reference ligands, indicating that only a subset of models can reliably reproduce native‐like binding modes. Among the contributions of similarity components, shape similarity exhibited the greatest variation across models, ranging from 0.268 for TamGen to 0.523 for DrugFlow‐PA, whereas electrostatic similarity remained relatively stable, within the range of 0.50‐0.59, suggesting that shape features predominantly accounted for the differences in binding mode similarity.

To further quantify the accuracy of the generated poses, we measured the centroid displacement between the generated poses and the original ligand. Non‐3D models exhibited substantially larger displacements. In contrast, DrugFlow and MolCraft reached displacements of ≤1 Å in their initial poses, even lower than those of reference ligands. Importantly, a negative correlation was observed between centroid displacement after docking and the Exact Match interaction metric (*R* ^2^ = 0.836), indicating that larger deviations in centroid position reduced the likelihood of maintaining key residue interactions, thereby highlighting the importance of accurate pose generation for effective molecular docking.

#### 2.4.4 Exploration of Initial Poses in 3D In Situ Paradigm

Based on an analysis of a series of docking‐related results (Tables S2,S5), we observed that redocking generally improved both the no‐clash rate and docking scores of 3D in situ models. Additionally, strong rescoring performance of initial poses was a prerequisite for successful redocking, reinforcing earlier concerns about the conformational reliability of 3D in situ frameworks [20]. However, the centroids of redocked conformations exhibited a clear tendency to deviate from the initial conformations relative to the co‐crystallized ligands, which also serve as sampling centers (Table S4). This displacement leads to reduced interaction match rates and binding mode similarity, suggesting that the spatial accuracy of initial pose prediction remains limited, likely due to steric clashes or the inability to capture energetically optimal configurations. This observation raises a new question: if we disregard suboptimal initial poses and instead fully reset the ligand conformations for docking, could this serve as a more precise evaluation strategy for identifying active compounds?

To explore this hypothesis, a subset of 3D in situ models with varying performance were selected for conformation‐reset docking (Table S6). Compared with redocking, conformation‐reset docking introduces an additional preprocessing step wherein the initial conformations are discarded and replaced with MMFF94‐optimized conformations. Because 3D in situ models tend to generate molecules within a predefined region, particularly around key residues, conformation‐reset docking offers a valuable complementary approach. In scenarios where both the initial conformations of the ligand and its predicted poses are suboptimal, this procedure facilitates the escape from local minima that cannot be overcome by standard redocking, enabling a more robust evaluation of models within this paradigm.

For all the models tested, conformation‐reset docking produced lower affinity, interaction match rates, and binding mode similarity, compared with redocking using initial poses. Nevertheless, the relative performance ranking of these models remained largely consistent, with DrugFlow, Lingo3DMol, and MolCraft continuing to outperform non‐3D baselines. Further comparisons were conducted to analyze pose displacement under the two docking procedures (Figure S5). In general, models that generated more plausible initial conformations (Table S7) achieved lower coordinate and aligned root‐mean‐square deviations (RMSDs) after re‐docking (median <2.5 Å), whereas those with less optimal initial conformations showed median RMSDs >5 Å. After conformation‐reset docking, coordinate RMSDs increased to varying degrees across all models, consistent with the observed trends in pose center displacement. Several cases were selected to illustrate the effects of conformation‐reset docking, where initial poses varied in plausibility but all exhibited substantial displacement after conformation‐reset docking (Figure 3e). Although the redocked poses showed low aligned RMSDs and partially corrected the original positions, subsequent conformation‐reset docking pushed the poses away from their initial locations and guided them toward regions that are likely more reasonable from a structural and energetic perspective. For instance, the fused‐ring system in DrugFlow moved into a broader cavity with only a minor loss in docking score, while the molecule in PocketFlow showed an improvement in its docking score after conformation‐reset docking.

Overall, these results indicate that conformation‐reset docking provides a supplemental and model‐independent assessment by decoupling ligand placement from the influence of initial conformational bias. Despite the decline in absolute performance metrics following conformation reset, the consistent ranking across models suggests that their relative performance is robust. Together, the systematic centroid displacement, reduced interaction recovery, and increased RMSDs highlight that current 3D in situ frameworks are still constrained by the accuracy of initial pose predictions, and evaluation protocols that reset ligand conformations can more effectively reveal genuine differences in models’ ability to recognize targets and generate plausible binding modes.

### 2.5 Analysis of Plausibility, Drug‐likeness, and Chemical Distance

#### 2.5.1 Molecular Plausibility

Molecular plausibility was primarily assessed at the 2D structural level. Here, both reference and random molecules are collectively termed “real compounds”, as generative models can generate molecules that are theoretically valid but may not be practically feasible.

In terms of basic structural properties (Table S8), certain graph‐based 3D in situ models, such as DrugFlow and IPDiff, exhibited incompleteness, likely due to the generation of disconnected graphs. Stereochemical features also diverged from those of real compounds, which typically contain, on average, fewer than one chiral center and ∼0.03 spiro atoms. Non‐3D models basically reproduced this trend, while diffusion‐based models within the 3D in situ paradigm generated an excessive amount of stereochemistry (>3.5 chiral centers and >0.08 spiro atoms), markedly undermining molecular plausibility. For molecular flexibility, most models generated molecules with an average of 4–7 torsional angles, and the fraction of rotatable bonds ranged between 0.2 and 0.3, in line with the characteristics of real compounds. However, SurfGen and IPDiff produced molecules with lower flexibility, likely attributable to their preference for generating rigid polycyclic scaffolds [43]. Additionally, the average ratio of heteroatoms, which contribute critically to interactions, was also considered. IPDiff and PocketFlow produced ratios <0.2, lower than those of real molecules and most other models, indicating an underrepresentation of heteroatoms that may compromise the drug‐likeness of the generated molecules.

Regarding ring‐associated properties (Table S9), most models generated molecules with an average of 3–5 rings, a distribution closely aligning with that observed in real compounds. Given the critical role of ring systems in drug‐like molecules, we further quantified the proportion of acyclic molecules, which was negligible in the reference ligands (0.035%), whereas all other models exhibited substantially higher proportions, ranging from 2 to 8 times those of random molecules and reaching up to 6.4%. For aromatic rings, the reference set averaged 3.226 per molecule, while most models yielded fewer than 3 aromatic rings, with some methods, such as IPDiff, generating fewer than one per molecule.

Rings that are overly complex or unstable may pose significant synthetic challenges or may not exist under real‐world conditions. Here, 3‐ and 8‐membered (or larger) rings were defined as undesired. Although these rings are typically valid structural motifs and appeared at a frequency of 15.8% in the reference compounds, most models generated them at much lower rates, with non‐3D models averaging below 10%. In stark contrast, DiffSBDD generated undesired rings in 42% of its molecules. For fused ring systems (Table S9), the majority of models produced an average of 0.5–0.8 fused ring systems per molecule, consistent with the distributions in real compounds. Non‐3D models exhibited slightly lower frequencies, averaging around 0.5. Bicyclic fused systems are important pharmacophores in drugs [44], but systems containing three or more fused rings increase molecular rigidity and synthetic difficulty, and were thus defined as highly fused [27]. Non‐3D models generally produced fewer highly fused rings than real molecules, whereas most 3D in situ models yielded markedly higher frequencies, with most exceeding 25%. SurfGen was an extreme outlier, with 74% of its molecules containing highly fused systems.

Overall, pretrained models, including all non‐3D models as well as Lingo3DMol and PocketFlow, demonstrated a tendency to avoid generating excessive stereochemistry or overly complex ring systems. The molecules they produced more closely resembled real compounds in terms of flexibility and complexity, thereby exhibiting better molecular plausibility. In contrast, other graph‐based 3D in situ models tended to overproduce implausible or synthetically inaccessible scaffolds, likely reflecting their limited coverage of the chemical space during training.

#### 2.5.2 Drug‐Likeness

Building on the structural analysis, we further examined drug‐likeness indices and structural alerts (Table S10). For the QED metric [45], non‐3D models generally outperformed others, with most scoring above 0.6, while the majority of other models ranged between 0.45 and 0.6. The reference compounds had an average QED score of 0.474, and only a few models fell slightly below this level. In terms of compliance with the classical empirical Lipinski's Rule‐of‐Five [46], nearly all models achieved ∼3.9 passes, a performance comparable to that of real compounds, except for the optimization‐based models DrugFlow‐PA and REINVENT. The evaluation of Synthetic accessibility (SA Score) [47] showed more pronounced differences among models. Sequence‐based models, regardless of their underlying paradigms, scored close to or below the reference average of 3, whereas graph‐based 3D in situ models, with the exception of PocketFlow, exceeded 3.5, with SurfGen and DiffSBDD reaching >4. Targeted optimization proved effective, as DrugFlow‐PA improved synthetic accessibility by 0.9 compared with its base model.

The analysis of structural alerts further highlighted model disparities. Matches to the PAINS [48] filters showed little variation across models and remained close to reference levels. In contrast, SureChEMBL alerts, which identify unfavorable medicinal chemistry groups [49], were notably higher in most 3D in situ models (∼0.3 hits, ∼50% above the reference), while all non‐3D models, except SimpleSBDD, performed better than real compounds. DrugFlow‐PA once again demonstrated targeted gains, reducing SureChEMBL matches by more than threefold relative to its base model. For Glaxo alerts [50], all models except REINVENT performed worse than the reference, although most were comparable to random molecules, with DiffSBDD emerging as the main outlier.

In summary, pretrained models generally achieved superior drug‐likeness and synthetic feasibility, but graph‐based 3D *in‐situ* models remained susceptible to the generation of undesirable structural motifs. Notably, property‐focused optimization mitigated these weaknesses, highlighting their value as a complementary strategy to pretraining in enhancing the overall quality and applicability of generated molecule.

#### 2.5.3 Chemical Distance

To further assess ligand‐level behavior within targets, we revisit Conjecture 2, which frames the assessment in terms of distances in the chemical space. We calculated two diversity indices to capture intra‐target diversity. For internal diversity (IntDiv) [18], our analysis revealed only minor variations across models, with values ranging from 0.85 to 0.9, which were close to those of random molecules but higher than those of active compounds (0.798). In contrast, the #Circle metric [51] was more discriminative: reference compounds yielded a value of 75, while random molecules scored 517. Most models exceeded a score of 100, with DiffSBDD and MolCraft surpassing the random baseline. These findings suggest that many models generate molecular sets that are overly dispersed, thereby increasing both the computational burden associated with constructing target‐focused libraries and the validation cost in downstream screening.

We then compared the chemical distances between generated molecules and both reference and random molecules, applying two categories of distance measures: embedding‐based and descriptor‐based. The former included the Tanimoto similarity from the ECFP4 fingerprints [52] and the Fréchet ChemNet Distance (FCD) [53] derived from deep‐learning embeddings. Descriptor‐based distances, introduced in this study, were defined as the average Wasserstein distance (Wass., Equation 3) across 15 physicochemical and structural descriptors that displayed significant differences between reference and random molecules (Figure S6):

(3) $$Wass=\frac{1}{n}\sum_{i=1}^{n}W\left(P_{i},Q_{i}\right)$$

where *W* denotes the Wasserstein distance for a single descriptor, *P* the test distribution, and *Q* the reference distribution.

**FIGURE 4:**
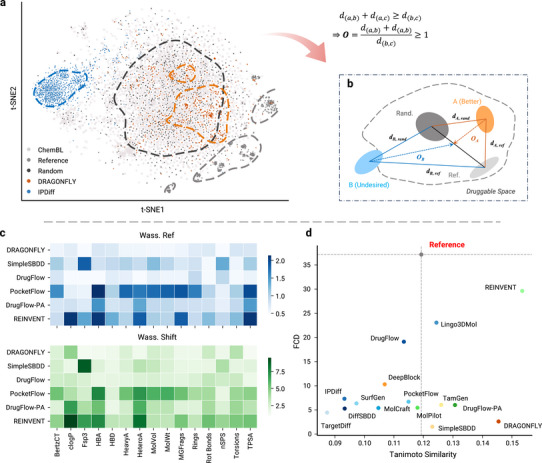
(a) Using JAK2 as an example, a t‐SNE projection of the ECFP4 fingerprints is shown to illustrate the chemical distance relationships of random molecules and reference actives relative to the molecules generated by a poorly performing (IPDiff) and a well‐performing model (DRAGONFLY), with ChEMBL compounds as the background representation of the drug‐like chemical space. The dashed regions represent probability density contours calculated using Gaussian KDE. (b) Abstracted from (a), we designed a relative shift metric (***O***), leveraging the triangle inequality properties of the Fréchet and Wasserstein distances and the advantage of shared edges in high‐dimensional distance geometry. The dashed arrows labeled ***O***_A_ and ***O***_B_ in the schematic are illustrative and do not indicate actual numeric magnitudes. (c) Heatmap showing the per‐descriptor contributions for the selected models. (d) Distribution of the inter‐target similarity plotted along two dimensions: FCD and Tanimoto similarity.

Since both reference and random molecules are derived from the bioactivity‐associated chemical space, we designed a shift index to quantify the extent to which generated molecules deviate from the drug‐like space, leveraging the triangle inequality of Fréchet and Wasserstein distances [54] (Equation 4). We use the triangle‐inequality‐based shift rather than reporting distance‐to‐reference and distance‐to‐random separately because the latter requires joint interpretation of two scale‐dependent quantities. The shift index summarizes the relative geometry among generated, reference, and random sets into a single normalized value, making comparisons across targets and across distance metrics more direct and less sensitive to baseline scale differences. In addition, we argue that within the similarity‐diversity trade‐off, maintaining closeness to the drug‐like space is essential for the selected two metrics that describe molecular properties. In contrast, Tanimoto similarity, which capture structural features, can be treated more flexibly to enable broader exploration of the chemical space and enhance molecular diversity. A graphical illustration of this concept is provided in Figure 4a,b:

(4) $$O=\frac{d\left(test,ref\right)+d\left(test,rand\right)}{d\left(rand,ref\right)},\;O\in \left[1,+\infty \right)$$

where *O* denotes the shift, and *d* represents either the FCD or Wasserstein distance.

To facilitate interpretation, we note that a shift value close to 1 indicates that the generated molecules remain approximately within the same drug‐like chemical space as the random ChEMBL baseline, whereas smaller values indicate closer agreement with the reference actives. In contrast, increasingly larger shift values indicate that the generated distribution departs from both the reference actives and the baseline drug‐like space.

As shown in Table 2, in embedding‐based distances, Tanimoto similarity showed limited discriminative power, with non‐3D and optimized methods achieving only modestly higher values. In contrast, FCD revealed larger differences: nearly all models scored worse than random molecules, with pre‐trained models clustering near 30 and others approaching 40. The corresponding FCD shifts revealed the same trend, with pre‐trained models maintaining values <2.5 and models prone to generating undesired structures exceeding 3, suggesting increasing divergence from the drug‐like chemical space. Optimized methods also displayed elevated shifts, indicating that tuning for single properties can drive molecules away from the drug‐like domain. Notably, two ChEMBL‐pretrained models achieved the lowest shifts (1.3–1.4), reflecting their close proximity to real compounds.

**TABLE 2:** Performance in internal diversity and in embedding‐ and descriptor‐based distance metrics.

Model		Diversity		Embeddings Dist. (↓)			Descriptors Dist. (↓)	
		IntDiv	#Circle	Sim. Ref (↑)	FCD Ref	FCD Shift	Wass. Ref	Wass. Shift
Non‐3D	DRAGONFLY	0.854	192	**0.127**	27.131	1.443	**0.629**	**2.166**
	DeepBlock	0.880	274	0.120	**22.982**	**1.313**	0.884	2.685
	SimpleSBDD	0.874	621	0.112	39.009	2.665	0.856	2.322
	TamGen	0.865	105	0.103	36.648	2.078	1.346	4.243
3D in situ	DiffSBDD	0.901	887	0.094	31.824	**2.093**	1.170	3.957
	DrugFlow	0.852	371	**0.130**	32.178	2.442	**0.633**	**2.512**
	IPDiff	0.889	467	0.069	50.292	3.830	1.906	6.896
	Lingo3DMol	0.830	35	0.122	**29.541**	2.131	0.822	3.408
	MolCraft	0.881	623	0.112	38.369	2.880	0.763	2.649
	PocketFlow	0.879	320	0.097	36.967	2.469	1.414	4.722
	SurfGen	0.875	206	0.110	42.562	3.245	1.122	4.126
	TargetDiff	0.882	211	0.096	44.594	3.497	1.233	4.719
Opt.	DrugFlow‐PA	0.836	151	**0.128**	42.897	3.371	0.955	3.658
	MolPilot	0.866	561	0.119	**41.495**	**3.208**	**0.796**	**3.156**
	REINVENT4	0.688	14	0.103	49.921	3.890	1.392	5.592
Reference		0.798	46 (75)^*^	—	—	—	—	—
Random		0.884	517	0.116	26.984	1	0.695	1

*Note: Because #Circle is sensitive to sample size and the number of reference compounds varies across targets, two values are reported: 46 represents the average across all targets, while 75 corresponds to the average for targets with 1000 reference compounds. Bold represents the best performance result in this paradigm.

Descriptor‐based metrics provided complementary and interpretable insights. The Wasserstein distance to reference molecules (Wass. Ref) demonstrated a significant correlation with FCD (ρ = 0.58, p = 0.02), validating its utility as a similarity measure. Similar to FCD, most models performed worse than random molecules, although ChEMBL‐pretrained models consistently ranked among the top performers. The Wasserstein shift metric proved more sensitive to outliers, amplifying deviations in specific descriptors (Figure 4c). For example, REINVENT showed a pronounced deviation in clogP (mean 6.59), consistent with its bias towards heteroatom, while DrugFlow‐PA diverged in terms of hydrogen‐bond acceptors, heteroatom counts, and TPSA, highlighting the potential risks of property‐focused optimization.

Finally, we examined whether molecules generated for different targets occupied distinct chemical spaces, as would be expected for target‐specific compounds. Cross‐target comparisons confirmed that no model exceeded reference molecules in terms of inter‐target similarity (Figure 4d). Most non‐3D models perform poorly in both FCD and Tanimoto similarity, with clusters concentrated in the lower‐right quadrant, suggesting potential overfitting. The Tanimoto similarity analysis against the CrossDocked2020 training set (Table S11) further reflects this trend, with pretrained non‐3D methods exhibiting notably higher similarity. In contrast, 3D in situ models benefit from greater structural diversity, resulting in lower Tanimoto similarity but higher FCD values, indicating suboptimal diversity in bioactivity‐relevant features.

### 2.6 Preliminary Exploration of Target Specificity

According to Conjecture 2, target‐aware molecular generation should be constrained by the specific conditions associated with each target, implying that molecules generated for different targets are expected to exhibit discernible differences, that is, target specificity. In the previous section, we briefly evaluated the similarity patterns among generated molecules across different targets for each model. In this section, we shift our focus to the targets themselves, conducting a preliminary exploration of whether specificity arises across different targets.

#### 2.6.1 Specificity from Protein Families Perspective

To explore molecular similarities across different protein targets, we initially calculated pairwise FCDs among all targets (Figure S7). As previously observed, non‐3D models exhibited potential overfitting tendencies, so we performed independent calculations for the non‐3D and 3D in situ paradigms. On average, the inter‐target FCD values for non‐3D models were markedly lower than those of 3D in situ models, and both were lower than reference values, consistent with our earlier findings. In both generation paradigms, kinases and GPCRs consistently showed lower intra‐family FCD differences, indicating a higher degree of molecular similarity within the same family. This can be attributed to their highly conserved nature and similar structural features, suggesting that the models can, to some extent, capture and incorporate target‐specific structural characteristics during molecular generation. Other protein families also indirectly support this conclusion. For instance, RXR‐alpha exhibited generally high FCD values when compared to most other targets but showed lower FCD with its family counterpart, PPAR‐alpha. In contrast, non‐kinase enzyme families, which are structurally and functionally diverse, demonstrated relatively small differences both within and between families.

To evaluate target specificity from the perspective of PLIs, we used docking score and interaction match rates as evaluation metrics. A two‐way ANOVA was conducted with model and target as factors for all generated molecules (Table S12). The results revealed significant main effects for both the model and the target, as well as an interaction effect, suggesting that both factors collectively influence molecular performance. Based on these statistical results, we performed post hoc tests to compare performance across different targets. The significance heatmaps (Figure S8) revealed that, for both redocking and rescoring, no significant differences were observed in docking scores or match rates within the same protein family. This suggests that models displayed comparable average performance across targets within a family, implying limited intra‐family heterogeneity. Although both indicators were affected by the intrinsic characteristics of the targets, our findings indicate that models still exhibited performance variations depending on target type. Overall, these results demonstrate that current target‐aware molecular generation exhibits a certain degree of target specificity at the protein family level.

#### 2.6.2 Consistency and Specificity in Similar Structures

**FIGURE 5:**
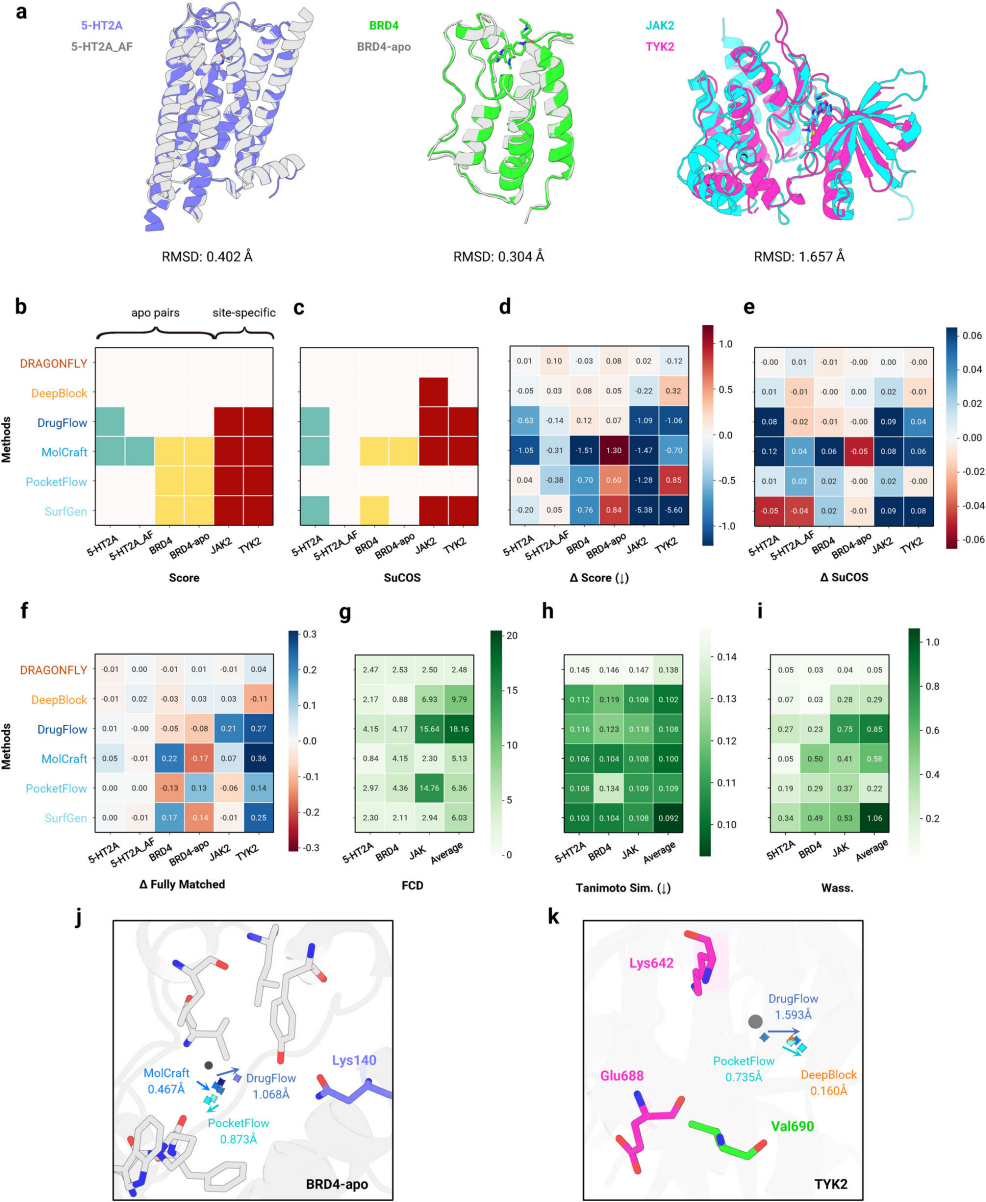
(a) Conformational alignment and RMSDs of the protein pairs in the consistency‐specificity test set. (b‐c) Significance heatmaps of the differences in docking scores (Score) and SuCOS between the molecules generated for the native target and those generated for the paired target after cross‐docking. Cells are colored when the difference is significant. The statistical procedure follows the description in the Methods section, except that two‐sided tests are used here. (d‐f) Differences in docking scores, SuCOS, and fully matched interaction recovery rates after cross‐docking. The blue direction indicates the ideal direction of change. (g‐i) Heatmaps showing differences in multiple molecular‐similarity metrics between the molecules generated for the native protein and those for the paired protein; darker colors indicate increased chemical dissimilarity. (j‐k) Shifts of the average ligand centroids relative to key residues after cross‐docking; the gray circle marks the center of the docking grid.

We curated a consistency‐specificity test set from the TarPass collection, which includes two categories of protein pairs. The *apo*‐*holo* pairs of 5‐HT2A and BRD4, with RMSDs of 0.402 and 0.304 Å, respectively (Figure 5a), serving as structurally matched targets for assessing generative consistency. The site‐specific JAK2‐TYK2 pair (RMSD = 1.657 Å), both belonging to the JAK family [55], differs in binding pockets (ATP‐binding pocket vs. pseudokinase domain), enabling an assessment of target specificity under substantial motif‐level differences.

We utilized FCD to quantify molecular similarity (Table S13). For the 5‐HT2A apo pair, most models yielded low FCDs, typically ranging from 1 to 4. However, for BRD4, more models exhibited above‐average FCDs. In contrast, for the site‐specific JAK2‐TYK2, none of the models demonstrated the expected target specificity relative to the corresponding reference ligands (∼18.6). Ideally, the FCD for apo pairs should be lower than that for the site‐specific pair, reflecting structural consistency and target specificity, respectively. Overall, only DeepBlock, DrugFlow, and PocketFlow followed this expected pattern, although it is noteworthy that DrugFlow relied on sampling from the original ligand. Most non‐3D models showed FCD values clustered around the cross‐target average, while several 3D in situ models (e.g., DiffSBDD, IPDiff) even displayed the opposite trend.

Further cross‐docking on models with distinct performance (Figure 5b–f) further highlighted these differences at the PLI level. Non‐3D models rarely produced significant distinctions for either apo or site‐specific pairs. DRAGONFLY showed strong overfitting, whereas DeepBlock captured limited PLI differences for the JAK2–TYK2 pair, suggesting that it preserved a modest degree of target‐specific discrimination. In contrast, the 3D in situ models exhibited more heterogeneous behaviors. The results were consistent for 5‐HT2A but varied markedly for the BRD4 apo‐holo pair. For JAK2‐TYK2, most produced marked differences favoring the original target across multiple PLI‐related metrics.

Building on our observations on PLIs, we further examined whether differences between specific molecules could be reflected as the deviations in the centroids of their docking poses. As shown in Table S14, non‐3D models showed uniformly small centroid shifts across all protein pairs, consistent with their smaller PLI discrepancies. Among the 3D in situ models, we observed a possible link between model performance and the relative displacement between the ligand centroid and key residues (Figure 5j,k). Specifically, in the cases of BRD4‐apo and TYK2, molecules originating from the non‐native proteins exhibited centroid shifts away from core residues, accompanied by inferior PLI performance compared to molecules generated from the native proteins. Moreover, although the sampling center selected for BRD4‐apo through structural alignment remained within the binding pocket, it was displaced from Lys140, a residue crucial for forming a key hydrogen bond. Given that some 3D in situ models only consider the pocket environment within 10 Å of the sampling center, this displacement may suggest that these models are overly sensitive to the local pocket environment, thereby failing to exhibit robustness in holo‐apo comparisons. Together with the irregular patterns observed across multiple chemical‐distance metrics (Figure 5g–i), these results imply that 3D in situ models may still struggle to sensitively and accurately discriminate between structurally similar proteins. The molecules generated by these models show inconsistent trends in structural similarity, pronounced centroid‐based positional deviations in PLI evaluation, and suboptimal and partly stochastic PLI behavior as previously demonstrated.

Overall, given that non‐3D models generally exhibit weak target specificity, the extent to which current 3D in situ models can achieve fine‐grained, truly target‐specific generation remains to be further validated and would benefit from more comprehensive and systematic evaluation, as assessments above are still based on a limited set of commonly studied protein families.

### 2.7 Post‐Processing via Multi‐Stage Virtual Screening Improving Model Usability

Despite the limitations observed in PLI and molecular plausibility, we explored whether the diversity advantage of well‐performing models could still be harnessed to enrich the pool of novel active compounds. To this end, drawing inspiration from previously reported cases [22, 28, 56, 57], we implemented a multi‐tier virtual screening workflow as a post‐processing strategy by utilizing hard constraints to prioritize molecules that perform well in terms of PLIs and molecular plausibility. Our approach aims to enhance the practical usability of generative models in realistic drug discovery settings and, to some extent, facilitates a comparative assessment of model performance differences based on screening outcomes.

The first stage applies hard filters based on PLIs, structural plausibility, and drug‐likeness, which are automated and customized using insights gained from the TarPass benchmark results. The subsequent stage employs more refined screening techniques, such as empirical soft filters or clustering, to further narrow down the candidate pool to a manageable and verifiable scale. As a test case, we selected JAK2 and TYK2 to enable both protocol assessment.

**FIGURE 6:**
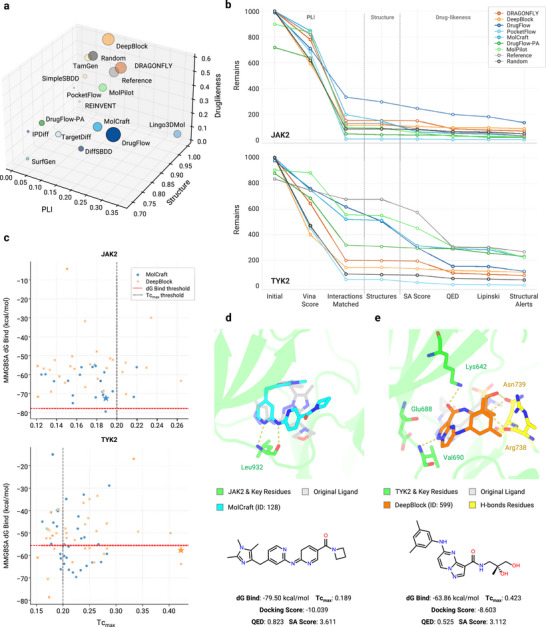
(a) Pass rates of the tested models across three evaluation criteria, with circle diameters indicating relative overall pass rates. (b) Trends in the number of remaining molecules during sequential filtering. (c) Scatter plots of ΔG binding free energies calculated by MM/GBSA and Tc_max_ values for molecules after downscaling to an appropriate range. Stars correspond to molecules shown in (d) and (e): docking poses, 2D structures, and related information of the selected molecules for JAK2 and TYK2. Yellow dashes indicate additional residues involved in reciprocal interactions.

In the first stage, we applied broad and conservative criteria to simulate the practical challenges associated with poorly characterized targets (see Methods). For JAK2, an analysis of the independent pass rates for the three criteria revealed that only 3D in situ models and their optimized variants achieved a success rate exceeding 40% at the PLI level, while non‐3D models performed slightly less effectively in this regard but retained an advantage in terms of structural feasibility and drug‐likeness, consistent with previous evaluations (Figure 6a). Sequential filtering of molecules generated by well‐performing models further demonstrated that the majority were eliminated due to mismatched key interactions or failure to meet the thresholds for SA Score and QED (Figure 6b), highlighting these properties as dominant bottlenecks in model outputs and clear priorities for future model refinement. Naturally, many reference compounds were also filtered out during these two stages, owing to limitations in docking accuracy and the lack of drug‐likeness optimization.

After applying the first‐stage hard filters across the three dimensions, both 3D in situ and non‐3D paradigms retained ∼10% of the molecules, suggesting that each paradigm holds practical applicability in drug discovery settings. However, the remaining pool of molecules was still too large for experimental validation. To address this challenge, we focused on two well‐performing representatives: MolCraft (3D in situ) and DeepBlock (non‐3D), and implemented further refinement. While clustering based on 2D similarity is a common strategy for downscaling libraries in virtual screening or molecular generation [56, 58], the high internal diversity of the chosen models rendered clustering ineffective in this case. Therefore, we applied experience‐based soft filters (see Methods) to further narrow down the pool to a manageable set of 20–30 molecules.

At this refined scale, we assessed the enrichment capability by calculating retrospective metrics derived from reference molecules. Binding free energies were calculated using the Molecular Mechanics/Generalized Born Surface Area (MM/GBSA) method, with the original ligand serving as the reference. Molecular similarity was evaluated via Tc_max_ [59] against the reference ligand (Figure 6c). To balance novelty and target relevance, we adopted a Tc_max_ cutoff of 0.2, below which molecules may risk insufficient specificity. Enrichment in both affinity and similarity was observed for JAK2 and TYK2, albeit with distinct patterns. For JAK2, most candidates fell slightly short of the thresholds but clustered within a region characterized by favorable binding energies and moderate novelty. In contrast, for TYK2, a larger proportion of molecules met both affinity and similarity criteria. From these regions, two representative molecules were selected for illustration (Figure 6d,e). Both exhibited chemically plausible structures and reasonable docking poses, but their interaction profiles revealed certain limitations: the JAK2 candidate did not show additional stabilizing interactions, while the TYK2 candidate did not sufficiently occupy the alanine pocket between Glu688 and Lys642 that governs selectivity [60]. These results indicate that, although enrichment was achieved and the molecules show signs of potential activity, they remain far from the quality expected of strong lead compounds.

In summary, this multi‐tier virtual screening strategy, which combines coarse and refined filtering as a post‐process strategy for current target‐aware molecular generation models, demonstrated a promising enrichment effect. The initial stage, which incorporated hard filters based on PLIs, structural plausibility, and drug‐likeness, reduced the candidate pool to approximately 10% of the original size. Subsequent fine‐grained filtering further narrowed the pool to a tractable scale for experimental validation. While the hard filtering stage prioritizes validity in PLIs and molecular plausibility over diversity such as constraining scaffold novelty, this trade‐off can be partially alleviated through the use of more flexible criteria in the soft filtering stage.

Depending on the specific target and the inherent performance of the generative model, this refinement can be flexibly guided by empirical soft filters, clustering, or molecular dynamics simulations, thereby enabling efficient enrichment and facilitating the prioritization of candidate compounds for downstream experimental testing. However, multi‐stage screening servers as a necessary but still insufficient bridge from generation to discovery, and highlights that further improvements must come from improving pose accuracy, interaction fidelity, and molecular plausibility, rather than relying solely on filtering strategies.

## 3 Discussion

With the rapid development of generative models, deep learning‐based target‐aware *de novo* molecular generators have emerged as promising tools for exploring novel chemical space in drug discovery. However, these models often struggle to capture the true nature of target‐ligand relationships. Furthermore, the limited chemical‐space coverage of training datasets further constrains the fidelity of generated molecules, ultimately restricting their applicability in practical drug discovery. To evaluate the genuine capabilities of such models, we introduce the TarPass benchmark, which assesses 15 models across three classical paradigms. Utilizing well‐characterized targets, we systematically evaluate their performance based on three key criteria: PLIs, structural plausibility, and drug‐likeness. By designing a standardized downstream evaluation protocol, we aim to improve the fairness of comparisons as much as possible; however, differences in training data, model capabilities, and input requirements mean that such evaluations cannot fully eliminate these inherent discrepancies.

Accurate modeling of PLIs represents the most fundamental requirement for target‐aware molecular generation. By analyzing multiple dimensions, we revisit the central question derived from Conjecture 1: although 3D in situ models explicitly incorporate spatial relationships and show a slight advantage in predicted affinities compared with non‐3D models, many of them fail to demonstrate significant improvements over random molecules in terms of both affinity and interaction recovery. Moreover, their performance is further compromised by poor initial conformations and inaccurate pose predictions. On the other hand, non‐3D models struggle to reconstruct PLIs by implicitly learning target‐ligand relationships, and even directly optimizing for affinity does not guarantee correct interaction matching. Approaches such as DrugFlow that sample from the original ligand improve PLI recovery, but these effects are confined to targets with favorable ligands, thereby limiting their broader applicability.

For structural plausibility and drug‐likeness, the non‐3D paradigm, dominated by sequence‐based models, excels due to their greater accessibility to pretraining on large drug‐like molecules, which enables the generation of molecules with improved drug‐likeness, synthetic accessibility, and fewer structural alerts. Similarly, graph‐based and 3D in situ models can also benefit from pretraining in this regard. In contrast, models without pretraining often produce undesirable structures, such as those with excessive stereocenters or complex ring systems, which impede their real‐world accessibility and validation. Property‐driven optimization within 3D in situ models can alleviate deficiencies in individual attributes; however, given the inherently multi‐objective nature of molecular design, this often comes at the cost of deteriorated performance in other properties. Analyses of chemical distance support these observations: pretrained models produce molecules that are closer to reference ligands and the drug‐like chemical space, and these molecules exhibit fewer deviations from real‐world compounds in both structural and property features.

In terms of target specificity, although these models can exhibit specificity at the protein family level, they continue to face challenges in accurately recognizing nuanced structural differences between closely related binding sites. Taken together, these results revisit Conjecture 2, indicating that current models still have difficulty in guiding generated molecules into biologically relevant and target‐specific regions of chemical space based solely on target information.

Overall, current target‐aware *de novo* molecular generation models are still far from achieving satisfactory design. Nevertheless, the molecules they produce are still retain practical value. To address these limitations, we developed a virtual screening workflow as a post‐processing step that applies hard filters based on PLIs, structural plausibility, and drug‐likeness to eliminate a large number of unfavorable molecules, followed by empirical refinement for meaningful enrichment, thereby supporting further validation. This workflow can, at a very low cost, filter out most undesirable molecules when deploying current generation models in practical drug discovery settings, identify valuable potential hits, and thereby improve the overall usability of the models.

In the results, models such as TamGen and PocketFlow that progressed to experimental validation tended to generate small, readily synthesizable molecules that are easy to obtain. However, these approaches generally exhibited limited performance in terms of PLIs, often requiring screening tens of thousands of candidates to identify molecules with only micromolar‐level activity. This suggests that such models primarily function as stochastic molecule generators rather than truly rational design tools. Consequently, claims of model superiority under such conditions are susceptible to the Texas Sharpshooter fallacy. Additionally, current modeling paradigms still suffer from notable limitations, including overfitting observed in pretrained models and the robustness of 3D in situ models in perceiving the binding pocket. More stringent use cases also warrant attention, including explicit treatment of metal ions, conserved water molecules, and other cofactors. Within the diversity‐similarity trade‐off, structural diversification becomes meaningful only when generated molecules already align with known actives in terms of PLI behavior and key molecular properties, thereby exhibiting plausible potential for target engagement. Simply maximizing novelty without ensuring such alignment is not desirable. Thus, to enable more targeted and effective molecular generation, future approaches should incorporate priors, such as interaction patterns characterized by precise physicality, to better capture the biophysical underpinnings of PLIs, while leveraging pretraining to broaden the coverage of chemical space.

## 4 Methods

### 4.1 Dataset Curation and Preparation

#### 4.1.1 Target Structures Dataset

The selected targets and their corresponding PDB IDs were derived from POKMOL‐3D [20], with the release date in the RCSB PDB [61] database used as the temporal criterion. Targets released after December 2019 were directly included. For other targets, if a structure with the same UniProt ID released after 2019 contained a ligand exhibiting the same pharmacological effect as that in the original structure, the dataset was updated with this newer entry. The remaining targets were excluded. For ligand‐free structures, we selected the AlphaFold‐predicted structure for 5‐HT2A [62] (5‐HT2A_AF) and the experimentally determined *apo* structure of BRD4 [63] (BRD4‐apo). Additionally, we chose MEK1 and TYK2 from the Allosteric Database [64] as allosteric targets, with ligands corresponding to type III and type VI allosteric inhibitors [65], respectively.

Unprocessed raw target structures were downloaded from the RCSB PDB. Preprocessing was performed using the Protein Preparation Wizard module in the Schrödinger 2018 suite. For each structure, only one monomer was retained and preprocessed by the following steps: adding hydrogens, assigning bond orders, filling in missing side‐chain atoms and loops, assigning protein protonation states at pH = 7.4 using PROPKA [66], and optimizing the hydrogen‐bonding network. Subsequently, unrelated small molecules and ions were removed, and the protein structure was separated from its original ligand. For ligand‐free structures, ligands were obtained by structural alignment with the respective *holo*‐form structures. A pseudo‐ligand consisting of only one single atom was additionally introduced for these two structures to enable supplementary testing. Furthermore, the full‐length structures were clipped as pocket structures, defined as residues within 10 Å of the original ligand, to ensure compatibility with certain models. This step was necessary because these models require explicit localization of the binding pocket or would incur prohibitively high computational costs when performing inference with the full‐length structure.

#### 4.1.2 Active Compounds Dataset

Active compounds were collected from the May 2025 version of BindingDB [35] by searching according to each target's UniProt ID. To address the complexity of targets with multiple binding pockets, the search results were further refined using the Target Name as an additional filter. For targets whose original ligands are antagonists or inhibitors, active compounds were selected based on the criterion of having a K_i_ or IC_50_ ≤ 1 µm. For agonists or other ligand types where no inhibitory activity was reported, the criteria were adjusted to K_d_ or EC_50_. The final output consisted of molecular SMILES strings. These molecules subsequently underwent additional filtering to remove salts, metal ions, isotopes, and incomplete structures. Furthermore, a molecular weight threshold of 750 Da was also applied to exclude PROTAC‐like compounds. For allosteric targets, such as MEK1 and BRAF, we collected all structures of the same allosteric type from the Kincore [67] database and retrieved their corresponding ligands. The Bemis‐Murcko (BM) scaffolds of these ligands were then used to match the filtered compounds, thereby ensuring a stringent selection of allosteric ligands. After applying the above filters, if the number of retained molecules exceeded 1000, the dataset was clustered by BM scaffold clustering, followed by random sampling to reduce redundancy.

### 4.2 Benchmark Protocols

#### 4.2.1 Preprocessing

For each target, the molecules generated by the models were saved either as SDF files or as SMILES strings in text format and then imported into the workflow. During import, validation checks were performed to eliminate any invalid molecules, followed by deduplication based on canonical SMILES. From the resulting set, only the first 1000 molecules were retained as the test set. This specific number was determined, inspired by the work of Özçelik et al. [14], to strike a balance between molecular diversity and computational cost (Figure S2).

#### 4.2.2 Docking and Protein‐Ligand Interaction Detection

The preprocessed full‐length protein structures described above were directly used as receptors in the docking process. Before docking, generated molecules required additional preparation through a pipeline based on RDKit [68] and Open Babel [69]. If a generated molecule did not have a known initial 3D conformation, a conformation was generated using ETKDGv3 [70, 71]. If this failed, the make3D function in Pybel [72] was used as an alternative. Subsequently, energy minimization was performed with the MMFF94 force field [73] for 200 steps. After that, molecules were subsequently protonated at pH = 7.4 with Open Babel. Docking was executed using Gnina 1.3 [74], an open‐source docking program derived from AutoDock Vina. The docking parameters were set as follows: exhaustiveness = 8; docking grid centered on the original ligand of the target; grid size = 20 Å × 20 Å × 20 Å; and the fast CNN scoring model was used to reduce computational cost. Only the top‐ranked docking pose was retained. The docked poses and their corresponding scores were stored in a pickle file for subsequent evaluation. After deprotonation (to avoid potential interactions detection errors), either the docked pose or the initial pose was combined with the receptor and saved as PDB files, which were then analyzed with PLIP to detect PLIs. The detected interactions were compared against the predefined key interactions, and the complete profiles along with their matching results were reported in JSON format.

#### 4.2.3 Evaluation and Analysis

The evaluation workflow is divided into two modules: MoleEval, which focuses on molecular properties, and DockEval, which focuses on PLI analysis. The MoleEval module directly reads either the preprocessed generated molecules or the pickle files generated during docking. It calculates a comprehensive array of molecular properties, including physicochemical descriptors (e.g., clogP), drug‐likeness metrics (e.g., QED), global structural descriptors (e.g., Fsp3 [75]), and topological structural descriptors such as BertzCT [76] and ring‐system‐related metrics. For descriptors that rely on Morgan fingerprints (e.g., SA Score [47]), the molecules are stripped of their initial 3D conformations before calculation in order to avoid bias. The DockEval module processes the files generated during docking. It quantifies the degree of similarity between the detected PLIs and the predefined key interactions, and evaluates docking poses and binding modes.

After obtaining the results from the two per‐molecule evaluation modules, the Analysis module performs further statistics and target‐level evaluations. For PLI‐related analysis, statistical evaluations are performed on the docking results, including significance testing of docking scores between generated molecules, reference ligands, and random molecules to analyze chemical distance. To assess the diversity of generated molecules, the ECFP4 fingerprints are used to calculate metrics such as internal diversity (IntDiv) and #Circle. These generated molecules are then compared against reference active molecules and random molecules using multiple chemical distance measures. For property‐based analysis, the module summarizes molecular attributes at the target level, including undesirable substructures and molecular plausibility. Detailed descriptions of all metrics can be found in Supporting Information.

#### 4.2.4 Statistical Comparison

We adopted the following procedure for significance testing in statistical comparison. First, we assessed the normality of the distributions of reference, random, and generated molecules using the D'Agostino and Pearson test. Since the distributions were generally non‐normal, we opted for non‐parametric methods for pairwise comparisons involving generated molecules using the Mann‐Whitney U test. Additionally, we reported Cliff's delta as the effect size measure to quantify the magnitude of the difference between the groups. The resulting *p*‐values were adjusted for multiple testing using the Benjamini‐Hochberg FDR correction. A null hypothesis was rejected when the adjusted *p*‐value was < 0.05 and the effect size exceeded 0.147 [77], indicating a statistically significant distributional difference. For docking scores, where our primary interest was in determining whether generated molecules achieved lower (and thus better) score distributions compared to the reference or random molecules, we performed one‐sided (left‐tailed) tests. All significance testing was performed with Python packages Scipy [78], statsmodels [79], and scikit‐posthocs [80].

All statistical comparisons are performed at the target level, and the aggregated summaries therefore reflect both model effects and variability across targets. A related limitation is that the presentation of uncertainty at this aggregated level does not explicitly disentangle model‐specific effects from differences in target difficulty. In practice, variations in binding‐site properties and interaction mechanisms may contribute substantially to cross‐target variability. Developing hierarchical or meta‐analytic approaches to better separate these factors and provide more rigorous uncertainty estimates remains an important direction for future work.

#### 4.2.5 Virtual Screening Protocols

For the two selected targets, the multilayer virtual screening protocol incorporated a series of stringent hard filters and empirical soft filters to ensure the selection of high‐quality candidate molecules. For the hard filters, at the PLI level, docking and rescoring scores were required to be ≤ −8, with all predefined key interactions fully satisfied. At the structural level, molecules were required to be complete, composed exclusively of common atoms (C, H, O, N, B, P, S, and halogens), with a maximum ring size of no more than seven atoms and a molecular weight between 250 and 750. For drug‐likeness, the SA Score was constrained to ≤ 4.0, QED to ≥ 0.4, with at least four Lipinski's rules satisfied, and no structural alerts present. For the experience‐based soft filters, at the PLI level, MolCraft candidates were restricted to molecules with re‐docking scores ≤ −10 and reset docking scores < −8, ensuring high predicted affinity, while DeepBlock was filtered by a SuCOS threshold ≥ 0.35 to preserve ligand‐consistent poses. Additional physicochemical constraints were applied, informed by empirical properties of approved kinase inhibitors (molecular weight 300–600, clogP 1–6) [81].

The MM‐GBSA calculations were performed based on the docking poses using the Prime MM‐GBSA module in the Schrödinger 2018 suite. The VSGB solvation model and the OPLS3 force field were employed. Protein flexibility was considered by defining residues within 5.0 Å of the ligand as flexible, and minimization was used as the sampling method.

### 4.3 Computational Environment

To ensure a fair evaluation of model performance such as runtime, model deployment, and molecule generation were conducted on an Ubuntu 22.04 server equipped with AMD EPYC 7K62 CPU and NVIDIA RTX 4090 GPU. Most computations within the benchmark were performed under the same environment, while certain CPU‐intensive tasks such as interaction detection were executed on server with an AMD EPYC 9654 CPU. For all tested models and methods, we respected the originally reported settings by employing the publicly available checkpoints and parameter configurations provided by the respective authors. The sampling batch size or total number of generated samples for each model is provided in Table S15.

## Author Contributions

Rui Qin, Jike Wang, Tingjun Hou, and Yu Kang conceptualized and designed the research study. Rui Qin, Zijie Chen, and Yurong Li constructed the dataset. Rui Qin and Zijie Chen wrote the code and collected all the data. Rui Qin, Zijie Chen, Longji Shen, Odin Zhang, Qinghan Wang, Qun Su, and Jike Wang performed the analysis. Rui Qin, Zijie Chen, Meijing Fang, Yilong Su, Jike Wang, Tingjun Hou, and Yu Kang wrote the paper. All authors read and approved the paper.

## Funding

This work was financially supported by the National Key Research and Development Program of China (2024YFA1307501), the National Natural Science Foundation of China (82373791), and the Central Guidance for Local Science and Technology Development Funds Project (2025ZY01022).

## Conflicts of Interest

None of the authors have a conflict of interest to disclose.

## Supporting information




**Supporting File**: advs75411‐sup‐0001‐SuppMat.docx.

## Data Availability

The code used in the study is publicly available from the GitHub repository (https://github.com/sorui‐qin/TarPass). Evaluation results for all test models or methods can be found at Zenodo (https://doi.org/10.5281/zenodo.17649772).
