# Supplementary material for: Revisiting Target‐Aware de novo Molecular Generation with TarPass: Between Rational Design and Texas Sharpshooter
Source: Adv Sci (Weinh). 2026 Apr 22;13(40):e75411. doi: 10.1002/advs.75411 (PMC13335704; doi:10.1002/advs.75411)
Supplement: Supplementary file 1 — Supporting File: advs75411‐sup‐0001‐SuppMat.docx. [file ADVS-13-e75411-s001.docx]

**Supplementary Information for**

**Revisiting Target-Aware *de novo* Molecular Generation with TarPass: Between Rational Design and Texas Sharpshooter**

**Contents**

[1 Supplementary Tables 2](#_Toc213163239)

[2 Supplementary Figures and Preliminary Results 19](#_Toc213163240)

[3 Details of Evaluation Metrics 27](#_Toc213163241)

[3.1 Binding Affinity and Interactions 27](#_Toc213163242)

[3.2 Binding modes 27](#_Toc213163243)

[3.3 Structural Properties 29](#_Toc213163244)

[3.4 Drug-likeness 30](#_Toc213163245)

[3.5 Structural Alerts 31](#_Toc213163246)

[3.6 Physicochemical Properties 32](#_Toc213163247)

[3.7 Chemical Distances 33](#_Toc213163248)

[4 Targets and Interactions Information 34](#_Toc213163249)

[4.1 Enzymes 34](#_Toc213163250)

[4.2 GPCRs 37](#_Toc213163251)

[4.3 Kinases 38](#_Toc213163252)

[4.4 Nuclear receptors 42](#_Toc213163253)

[4.5 Other targets 43](#_Toc213163254)

[5. Introduction to Models or Methods for Evaluation 46](#_Toc213163255)

[References 49](#_Toc213163256)

# 1 Supplementary Tables

**Supplementary Table 1.** Relevant information on the test targets

| Target | PDB ID | Release Year | Resolution (Å) | Classification | Ligand Type | Highest Clinical Stage |
| --- | --- | --- | --- | --- | --- | --- |
| 5-HT2A | 7WC7 | 2022 | 2.60 | GPCR | Inhibitor | Approved |
| 5-HT2A_AF | AFP28223F1 | 2021 | - | - | - | - |
| BCL2 | 8HTS | 2024 | 1.25 | Apoptosis regulator (Other) | Inhibitor | Approved |
| Beta2AR | 8JJL | 2023 | 3.20 | GPCR | Agonist | Approved |
| BRAF | 6XFP | 2021 | 2.00 | Kinase | Type II inhibitor | Approved |
| BRD4 | 8PXA | 2023 | 1.30 | Epigenetic regulator (Other) | Inhibitor | Phase III |
| BRD4-apo | 2OSS | 2007 | 1.35 | - | - | - |
| BTK | 8FLL | 2023 | 1.50 | Kinase | Inhibitor | Approved |
| DRD2 | 7DFP | 2020 | 3.10 | GPCR | Antagonist | Approved |
| HDAC6 | 8BJK | 2022 | 1.35 | Enzyme | Inhibitor | Approved |
| HIV-RT | 8FCD | 2022 | 2.57 | Enzyme | Inhibitor (NNRTI) | Approved |
| JAK2 | 8BM2 | 2023 | 1.50 | Kinase | Inhibitor | Approved |
| MEK1 | 7M0Y | 2021 | 3.45 | Kinase | Type III inhibitor | Approved |
| NAMPT | 7PPE | 2022 | 1.86 | Enzyme | Inhibitor | Phase I |
| PI3K-alpha | 7PG6 | 2022 | 2.50 | Kinase | Inhibitor | Approved |
| PPAR-alpha | 6KAY | 2020 | 1.74 | Nuclear receptor | Antagonist | Approved |
| PRMT5 | 7S1S | 2022 | 2.62 | Enzyme | MTA-cooperative inhibitor | Phase III |
| ROCK1 | 7JOU | 2020 | 3.32 | Kinase | Inhibitor | Approved |
| RXR-alpha | 8PP0 | 2024 | 1.90 | Nuclear receptor | Agonist | Approved |
| TYK2 | 8TB5 | 2022 | 2.32 | Kinase | Type IV inhibitor  (JH2 domain) | Approved |

Data on the highest clinical stage were obtained from the Pharmacodia database as of January 2025. Since BRD4-apo is not included in the training set, its public release time was not considered when performing the time-split criterion

**Supplementary Table 2.** The performance of the models in terms of binding affinity

| Model | | Vina Score  (Med., ↓) | | Vina Score  (Q1, ↓) | | LE | | Improved vs Ref. | | Improved vs Rand. | |
| --- | --- | --- | --- | --- | --- | --- | --- | --- | --- | --- | --- |
|  |  | Dock | Score | Dock | Score | Dock | Score | Dock | Score | Dock | Score |
| Non-3D | DRAGONFLY | -8.192 | - | -9.329 | - | 0.261 |  | 0 | - | 0.85 |  |
|  | DeepBlock | -7.776 | - | -8.749 | - | 0.339 |  | 0 | - | 0.25 |  |
|  | SimpleSBDD | -8.230 | - | -8.974 | - | 0.319 |  | 0 | - | 0.25 |  |
|  | TamGen | -6.890 | - | -7.581 | - | 0.357 |  | 0 | - | 0.05 |  |
| 3D  *in-situ* | DiffSBDD | -8.032 | 6.966 | -9.188 | -0.208 | 0.326 | 0.253 | 0 | 0 | 0.30 | 0.10 |
|  | DrugFlow | -9.776 | -8.494 | -10.689 | -9.569 | 0.335 | 0.283 | 0.65 | 0.20 | 0.90 | 0.50 |
|  | IPDiff | -7.718 | -7.564 | -9.420 | -9.399 | 0.421 | 0.411 | 0.05 | 0.20 | 0.30 | 0.45 |
|  | Lingo3DMol | -9.024 | -3.713 | -9.734 | -5.278 | 0.285 | 0.134 | 0.20 | 0 | 0.90 | 0 |
|  | MolCraft | -9.987 | -9.470 | -11.278 | -10.722 | 0.350 | 0.333 | 0.70 | 0.55 | 0.90 | 0.80 |
|  | PocketFlow | -7.795 | -4.805 | -9.117 | -6.211 | 0.412 | 0.276 | 0 | 0 | 0.20 | 0 |
|  | SurfGen | -9.531 | -6.808 | -10.945 | -8.338 | 0.377 | 0.255 | 0.50 | 0.10 | 0.65 | 0.20 |
|  | TargetDiff | -7.583 | -5.901 | -8.865 | -7.394 | 0.399 | 0.347 | 0.05 | 0.05 | 0.20 | 0.10 |
| Opt. | DrugFlow-PA | -10.374 | -9.449 | -11.382 | -10.413 | 0.404 | 0.347 | 0.70 | 0.55 | 0.95 | 0.75 |
|  | MolPilot | -10.483 | -10.279 | -11.331 | -11.084 | 0.338 | 0.348 | 0.95 | 0.80 | 0.95 | 0.95 |
|  | REINVENT4 | -10.023 | - | -10.741 | - | 0.367 | - | 0.70 | - | 0.90 | - |
| Reference | | -9.216 | - | -9.953 | - | 0.295 | - | - | - | 0.90 | - |
| Random | | -7.835 | - | -8.848 | - | 0.293 | - | 0 | - | - | - |

**LE**, ligand efficiency. **Improved vs. xx** refers to the proportion of targets for which the binding affinity is significantly better compared with either the reference or random molecules.

**Supplementary Table 3.** Statistics of the parts of physicochemical descriptors used in chemical distance measure.

| Model | | clogP | HBD | HBA | MolWt | TPSA | MolVol | Heavy  atoms | Hetero  atoms | Fsp3 |
| --- | --- | --- | --- | --- | --- | --- | --- | --- | --- | --- |
| Non-3D | DRAGONFLY | 4.51 | 1.95 | 5.14 | 460.58 | 87.06 | 415.91 | 33.27 | 7.99 | 0.33 |
|  | DeepBlock | 2.80 | 1.95 | 4.77 | 344.69 | 82.29 | 302.86 | 24.35 | 6.80 | 0.32 |
|  | SimpleSBDD | 3.33 | 1.10 | 4.38 | 375.31 | 62.63 | 303.98 | 26.47 | 5.82 | 0.01 |
|  | TamGen | 1.52 | 2.67 | 4.42 | 281.58 | 91.16 | 248.47 | 19.49 | 6.36 | 0.42 |
| 3D  *in-situ* | DiffSBDD | 2.26 | 2.74 | 5.83 | 381.60 | 93.08 | 338.72 | 26.50 | 7.56 | 0.60 |
|  | DrugFlow | 3.69 | 2.30 | 5.38 | 428.05 | 93.96 | 368.53 | 30.43 | 7.99 | 0.30 |
|  | IPDiff | 4.25 | 0.98 | 1.69 | 278.63 | 29.17 | 265.30 | 20.32 | 1.96 | 0.64 |
|  | Lingo3DMol | 2.79 | 2.96 | 6.98 | 473.36 | 120.06 | 390.58 | 32.91 | 10.67 | 0.33 |
|  | MolCraft | 3.70 | 2.25 | 4.96 | 410.43 | 84.31 | 355.89 | 29.44 | 7.33 | 0.34 |
|  | PocketFlow | 4.17 | 0.89 | 1.76 | 284.57 | 33.67 | 252.84 | 20.48 | 3.29 | 0.24 |
|  | SurfGen | 3.23 | 1.93 | 5.42 | 372.04 | 84.89 | 312.07 | 27.96 | 6.32 | 0.16 |
|  | TargetDiff | 2.14 | 2.06 | 3.74 | 282.24 | 61.80 | 253.73 | 20.33 | 4.78 | 0.50 |
| Opt. | DrugFlow-PA | 5.19 | 1.16 | 2.81 | 366.24 | 46.20 | 322.93 | 26.69 | 4.23 | 0.20 |
|  | MolPilot | 3.03 | 2.64 | 6.91 | 435.71 | 113.69 | 389.03 | 32.10 | 8.33 | 0.33 |
|  | REINVENT4 | 6.59 | 0.68 | 1.67 | 378.03 | 26.71 | 338.76 | 28.03 | 2.88 | 0.08 |
| Reference | | 4.09 | 1.98 | 5.79 | 453.01 | 89.67 | 395.01 | 32.10 | 8.38 | 0.31 |
| Random | | 3.38 | 1.55 | 5.33 | 404.90 | 82.86 | 355.45 | 28.45 | 7.56 | 0.34 |

All details of descriptors can be found at Chapter 3.

**Supplementary Table 4.** The performance of the models in terms of interactions

| Model | | Exact Match | | Match Ratio | | Detected Int. | |
| --- | --- | --- | --- | --- | --- | --- | --- |
|  |  | Dock | Score | Dock | Score | Dock | Score |
| Non-3D | DRAGONFLY | 0.249 | - | 0.482 |  | 16.006 | - |
|  | DeepBlock | 0.276 | - | 0.495 |  | 13.364 | - |
|  | SimpleSBDD | 0.166 | - | 0.419 |  | 13.788 | - |
|  | TamGen | 0.221 | - | 0.424 |  | 12.478 | - |
| 3D  *in-situ* | DiffSBDD | 0.313 | 0.370 | 0.536 | 0.608 | 16.094 | 16.582 |
|  | DrugFlow | 0.522 | 0.598 | 0.733 | 0.800 | 17.502 | 17.889 |
|  | IPDiff | 0.145 | 0.147 | 0.350 | 0.403 | 11.297 | 11.593 |
|  | Lingo3DMol | 0.332 | 0.351 | 0.577 | 0.578 | 16.143 | 15.140 |
|  | MolCraft | 0.456 | 0.531 | 0.676 | 0.744 | 16.689 | 16.850 |
|  | PocketFlow | 0.181 | 0.156 | 0.395 | 0.380 | 11.602 | 9.264 |
|  | SurfGen | 0.235 | 0.213 | 0.458 | 0.411 | 13.096 | 11.493 |
|  | TargetDiff | 0.232 | 0.215 | 0.443 | 0.470 | 12.057 | 11.140 |
| Opt. | DrugFlow-PA | 0.417 | 0.463 | 0.642 | 0.697 | 15.507 | 15.331 |
|  | MolPilot | 0.430 | 0.428 | 0.658 | 0.664 | 18.048 | 17.759 |
|  | REINVENT4 | 0.173 | - | 0.403 | - | 14.164 | - |
| Reference | | 0.514 | - | 0.692 | - | 16.329 | - |
| Random | | 0.223 | - | 0.447 | - | 13.817 | - |

Detected Int.: Detected Interactions.

**Supplementary Table 5.** The performance of the models in terms of initial and docked poses

| Model | | No-clash | | SuCOS | | Shape Sim. | | ESP Sim. | | Cent. Displ. (Å) | |
| --- | --- | --- | --- | --- | --- | --- | --- | --- | --- | --- | --- |
|  |  | Dock | Score | Dock | Score | Dock | Score | Dock | Score | Dock | Score |
| Non-3D | DRAGONFLY | 0.887 | - | 0.283 | - | 0.304 | - | 0.518 | - | 3.124 | - |
|  | DeepBlock | 0.950 | - | 0.259 | - | 0.304 | - | 0.523 | - | 3.180 | - |
|  | SimpleSBDD | 0.977 | - | 0.271 | - | 0.316 | - | 0.512 | - | 2.939 | - |
|  | TamGen | 0.977 | - | 0.213 | - | 0.268 | - | 0.515 | - | 3.709 | - |
| 3D  *in-situ* | DiffSBDD | 0.931 | 0.559 | 0.283 | 0.306 | 0.331 | 0.364 | 0.523 | 0.525 | 2.876 | 2.336 |
|  | DrugFlow | 0.913 | 0.797 | 0.374 | 0.445 | 0.427 | 0.517 | 0.555 | 0.569 | 1.710 | 0.777 |
|  | IPDiff | 0.980 | 0.864 | 0.221 | 0.277 | 0.267 | 0.349 | 0.522 | 0.518 | 3.902 | 1.388 |
|  | Lingo3DMol | 0.969 | 0.629 | 0.322 | 0.313 | 0.349 | 0.338 | 0.529 | 0.532 | 2.373 | 1.945 |
|  | MolCraft | 0.944 | 0.849 | 0.352 | 0.399 | 0.408 | 0.468 | 0.538 | 0.546 | 1.864 | 1.016 |
|  | PocketFlow | 0.978 | 0.767 | 0.243 | 0.240 | 0.278 | 0.287 | 0.523 | 0.513 | 3.582 | 2.088 |
|  | SurfGen | 0.923 | 0.653 | 0.270 | 0.342 | 0.307 | 0.414 | 0.516 | 0.504 | 3.376 | 1.979 |
|  | TargetDiff | 0.967 | 0.861 | 0.234 | 0.267 | 0.289 | 0.345 | 0.522 | 0.521 | 3.321 | 1.258 |
| Opt. | DrugFlow-PA | 0.959 | 0.922 | 0.364 | 0.433 | 0.426 | 0.523 | 0.569 | 0.585 | 1.856 | 0.782 |
|  | MolPilot | 0.966 | 0.926 | 0.361 | 0.405 | 0.424 | 0.485 | 0.532 | 0.535 | 1.786 | 1.023 |
|  | REINVENT4 | 0.977 | - | 0.292 | - | 0.319 | - | 0.527 | - | 2.993 | - |
| Reference | | 0.935 | - | 0.347 | - | 0.364 | - | 0.558 | - | 2.471 | - |
| Random | | 0.917 | - | 0.264 | - | 0.303 | - | 0.517 | - | 3.222 | - |

**Sim**.: similarity. **Cent. Displ.**: centroid displacement.

**Supplementary Table 6.** Results of conformation-reset docking for the selected models

| Metrics | | DrugFlow | IPDiff | Lingo3DMol | MolCraft | PocketFlow |
| --- | --- | --- | --- | --- | --- | --- |
| Vina Score  (Med, ↓) | Reset | -8.813 | -6.602 | -8.915 | -8.393 | -7.565 |
|  | Δ | 0.963 | 1.116 | 0.109 | 1.594 | 0.230 |
| Vina Score  (Q1, ↓) | Reset | -9.728 | -7.889 | -9.687 | -9.627 | -8.830 |
|  | Δ | 0.961 | 1.531 | 0.047 | 1.651 | 0.287 |
| LE | Reset | 0.305 | 0.371 | 0.281 | 0.308 | 0.404 |
|  | Δ | -0.030 | -0.050 | -0.004 | -0.042 | -0.008 |
| Improved vs  Ref. | Reset | 0.15 | 0 | 0.25 | 0.10 | 0 |
|  | Δ | -0.50 | -0.05 | 0.05 | -0.60 | 0 |
| Improved vs  Rand. | Reset | 0.70 | 0.05 | 0.90 | 0.55 | 0.15 |
|  | Δ | -0.20 | -0.25 | 0 | -0.35 | -0.05 |
| Exact Match | Reset | 0.412 | 0.119 | 0.336 | 0.345 | 0.178 |
|  | Δ | -0.110 | -0.026 | 0.004 | -0.111 | -0.003 |
| Match Ratio | Reset | 0.633 | 0.308 | 0.572 | 0.562 | 0.394 |
|  | Δ | -0.100 | -0.042 | -0.005 | -0.114 | -0.001 |
| Detected Int. | Reset | 16.410 | 10.415 | 16.219 | 15.140 | 11.751 |
|  | Δ | -1.092 | -0.882 | 0.076 | -1.549 | 0.149 |
| No-clash | Reset | 0.861 | 0.934 | 0.965 | 0.865 | 0.969 |
|  | Δ | -0.052 | -0.046 | -0.004 | -0.079 | -0.009 |
| SuCOS | Reset | 0.321 | 0.183 | 0.315 | 0.288 | 0.241 |
|  | Δ | -0.053 | -0.038 | -0.007 | -0.064 | -0.002 |
| Shape Sim. | Reset | 0.364 | 0.217 | 0.342 | 0.331 | 0.275 |
|  | Δ | -0.063 | -0.050 | -0.007 | -0.077 | -0.003 |
| Esp Sim. | Reset | 0.534 | 0.519 | 0.520 | 0.524 | 0.523 |
|  | Δ | -0.021 | -0.003 | -0.009 | -0.014 | 0.000 |
| Cent. Disp. (Å) | Reset | 2.367 | 4.752 | 2.502 | 2.773 | 3.633 |
|  | Δ | 0.657 | 0.850 | 0.129 | 0.909 | 0.051 |

**Supplementary Table 7.** Conformational check results of part of 3D in-situ models and their optimized variants with PoseBusters

| Metrics | DrugFlow | IPDiff | Lingo3DMol | MolCraft | PocketFlow | DrugFlow-PA | MolPilot |
| --- | --- | --- | --- | --- | --- | --- | --- |
| All atoms connected | 0.923 | 0.923 | 1 | 1 | 1 | 0.882 | 0.994 |
| Bond lengths | 0.912 | 0.909 | 0.910 | 0.998 | 0.756 | 0.687 | 0.946 |
| Bond angles | 0.951 | 0.575 | 0.647 | 0.979 | 0.958 | 0.809 | 0.998 |
| Internal steric clash | 0.950 | 0.897 | 0.638 | 0.979 | 0.922 | 0.896 | 0.995 |
| Aromatic ring flatness | 0.999 | 0.998 | 0.996 | 1 | 0.961 | 0.997 | 0.999 |
| Non-aromatic ring non-flatness | 0.989 | 0.939 | 0.999 | 0.798 | 0.973 | 0.999 | 0.984 |
| Double bond flatness | 0.983 | 0.995 | 0.999 | 1 | 0.987 | 0.998 | 0.988 |
| Internal energy | 0.925 | 0.502 | 0.628 | 0.947 | 0.821 | 0.857 | 0.983 |

**Supplementary Table 8.** Statistics of the basic structural properties of model-generated molecules.

| Model | | Completeness | Chiral Atoms | Spiro Atoms | Torsion Angles | Flexibility | Spacial Score | Heteroatom  Ratio |
| --- | --- | --- | --- | --- | --- | --- | --- | --- |
| Non-3D | DRAGONFLY | 1 | 0.372 | 0.023 | 8.767 | 0.376 | 14.775 | 0.238 |
|  | DeepBlock | 1 | 0.879 | 0.005 | 5.148 | 0.265 | 16.248 | 0.283 |
|  | SimpleSBDD | 1 | 1.319 | 0.030 | 5.791 | 0.265 | 20.042 | 0.220 |
|  | TamGen | 1 | 1.239 | 0.002 | 4.085 | 0.277 | 18.235 | 0.325 |
| 3D  *in-situ* | DiffSBDD | 1 | 3.841 | 0.085 | 7.295 | 0.434 | 26.951 | 0.290 |
|  | DrugFlow | 0.923 | 1.647 | 0.030 | 6.081 | 0.240 | 18.241 | 0.263 |
|  | IPDiff | 0.933 | 3.681 | 0.106 | 2.163 | 0.163 | 33.930 | 0.101 |
|  | Lingo3DMol | 1 | 1.629 | 0 | 8.615 | 0.304 | 16.287 | 0.327 |
|  | MolCraft | 1 | 1.889 | 0.014 | 5.164 | 0.239 | 20.750 | 0.248 |
|  | PocketFlow | 1 | 0.836 | 0.007 | 4.063 | 0.255 | 15.117 | 0.160 |
|  | SurfGen | 1 | 0.965 | 0.011 | 1.729 | 0.064 | 17.939 | 0.231 |
|  | TargetDiff | 1 | 2.422 | 0.024 | 3.025 | 0.231 | 26.842 | 0.239 |
| Opt. | DrugFlow-PA | 0.882 | 0.690 | 0.037 | 4.365 | 0.200 | 14.418 | 0.160 |
|  | MolPilot | 0.994 | 1.532 | 0.011 | 5.870 | 0.197 | 18.571 | 0.257 |
|  | REINVENT4 | 1 | 0.046 | 0 | 4.115 | 0.177 | 11.198 | 0.117 |
| Reference | | 1 | 0.546 | 0.028 | 7.070 | 0.259 | 15.503 | 0.260 |
| Random | | 0.956 | 0.896 | 0.037 | 6.682 | 0.282 | 16.348 | 0.266 |

**Supplementary Table 9.** Statistics of the ring-related properties of model-generated molecules.

| Model | | Rings Properties | | | | Fused Rings | | |
| --- | --- | --- | --- | --- | --- | --- | --- | --- |
|  |  | Ring Number | Acyclic% | Undesired Rings% | Aromatic Rings | Fused Number | Fused Ratio | Highly Fused% |
| Non-3D | DRAGONFLY | 3.945 | 0.850 | 10.890 | 2.125 | 0.515 | 0.485 | 5.260 |
|  | DeepBlock | 3.006 | 2.150 | 6.070 | 2.940 | 0.574 | 0.530 | 11.960 |
|  | SimpleSBDD | 3.620 | 0.058 | 7.648 | 1.831 | 0.599 | 0.534 | 8.837 |
|  | TamGen | 1.966 | 2.268 | 4.611 | 1.294 | 0.357 | 0.356 | 4.305 |
| 3D  *in-situ* | DiffSBDD | 2.984 | 6.405 | 42.366 | 0.736 | 0.588 | 0.504 | 17.860 |
|  | DrugFlow | 3.991 | 0.205 | 19.961 | 2.492 | 0.745 | 0.664 | 27.449 |
|  | IPDiff | 3.165 | 2.913 | 22.790 | 0.299 | 0.710 | 0.665 | 42.270 |
|  | Lingo3DMol | 3.525 | 0.090 | 5.370 | 2.835 | 0.437 | 0.395 | 0.065 |
|  | MolCraft | 4.075 | 1.270 | 7.316 | 1.844 | 0.877 | 0.711 | 38.286 |
|  | PocketFlow | 2.403 | 4.195 | 2.290 | 1.868 | 0.372 | 0.356 | 13.205 |
|  | SurfGen | 5.503 | 0.152 | 6.470 | 2.852 | 1.057 | 0.904 | 74.114 |
|  | TargetDiff | 2.835 | 3.734 | 19.113 | 0.597 | 0.661 | 0.619 | 34.017 |
| Opt. | DrugFlow-PA | 4.051 | 0.581 | 22.132 | 3.225 | 0.737 | 0.648 | 25.703 |
|  | MolPilot | 4.745 | 0.016 | 5.130 | 2.728 | 1.164 | 0.885 | 49.789 |
|  | REINVENT4 | 4.330 | 1.035 | 1.205 | 4.252 | 0.849 | 0.647 | 13.545 |
| Reference | | 4.279 | 0.035 | 15.845 | 3.226 | 0.899 | 0.732 | 12.348 |
| Random | | 3.496 | 0.800 | 7.700 | 2.508 | 0.588 | 0.523 | 12.600 |

**Supplementary Table 10.** Statistics of the drug-likeness properties and structural alerts of model-generated molecules.

| Model | | Drug-likeness | | | Alerts (↓) | | |
| --- | --- | --- | --- | --- | --- | --- | --- |
|  |  | QED | SA Score  (↓) | Lipinski | PAINS | SureChEMBL | Glaxo |
| Non-3D | DRAGONFLY | 0.469 | 2.648 | 3.956 | 0.032 | 0.137 | 0.050 |
|  | DeepBlock | 0.623 | 2.905 | 3.906 | 0.050 | 0.165 | 0.048 |
|  | SimpleSBDD | 0.622 | 3.777 | 3.906 | 0.053 | 0.378 | 0.215 |
|  | TamGen | 0.606 | 3.263 | 3.951 | 0.050 | 0.153 | 0.037 |
| 3D  *in-situ* | DiffSBDD | 0.461 | 4.654 | 3.930 | 0.033 | 0.526 | 0.280 |
|  | DrugFlow | 0.525 | 3.660 | 3.935 | 0.031 | 0.282 | 0.092 |
|  | IPDiff | 0.570 | 4.421 | 3.664 | 0.014 | 0.339 | 0.072 |
|  | Lingo3DMol | 0.394 | 3.573 | 4.022 | 0.039 | 0.295 | 0.069 |
|  | MolCraft | 0.546 | 3.998 | 3.886 | 0.039 | 0.281 | 0.079 |
|  | PocketFlow | 0.563 | 2.805 | 3.708 | 0.008 | 0.448 | 0.092 |
|  | SurfGen | 0.474 | 3.946 | 3.955 | 0.064 | 0.306 | 0.069 |
|  | TargetDiff | 0.609 | 4.363 | 3.929 | 0.022 | 0.307 | 0.080 |
| Opt. | DrugFlow-PA | 0.575 | 2.771 | 3.482 | 0.015 | 0.081 | 0.052 |
|  | MolPilot | 0.499 | 3.791 | 3.845 | 0.054 | 0.272 | 0.052 |
|  | REINVENT4 | 0.398 | 2.187 | 3.288 | 0.026 | 0.238 | 0.011 |
| Reference | | 0.474 | 2.995 | 3.960 | 0.042 | 0.188 | 0.025 |
| Random | | 0.563 | 2.888 | 3.987 | 0.053 | 0.196 | 0.068 |

**Supplementary Table 11.** Average Tanimoto similarity and the maximum Tanimoto similarity (Tc_max_) between generated molecules and CrossDocked2020 training set.

| Model | | Tanimoto Sim.  (Avg.) | Tc_max_ | |
| --- | --- | --- | --- | --- |
|  |  |  | Mean | Max |
| Non-3D | DRAGONFLY | 0.107 | 0.390 | 0.397 |
|  | DeepBlock | 0.100 | 0.506 | 0.570 |
|  | SimpleSBDD | 0.047 | 0.121 | 0.125 |
|  | TamGen | 0.102 | 0.479 | 0.528 |
| 3D  *in-situ* | DiffSBDD | 0.086 | 0.245 | 0.282 |
|  | DrugFlow | 0.088 | 0.239 | 0.327 |
|  | IPDiff | 0.062 | 0.233 | 0.294 |
|  | Lingo3DMol | 0.098 | 0.280 | 0.320 |
|  | MolCraft | 0.078 | 0.212 | 0.280 |
|  | PocketFlow | 0.087 | 0.335 | 0.376 |
|  | SurfGen | 0.066 | 0.198 | 0.243 |
|  | TargetDiff | 0.073 | 0.226 | 0.291 |
| Opt. | DrugFlow-PA | 0.072 | 0.204 | 0.244 |
|  | MolPilot | 0.081 | 0.205 | 0.261 |
|  | REINVENT4 | 0.088 | 0.358 | 0.422 |
| Reference | | 0.101 | 0.380 | 0.530 |
| Random | | 0.101 | 0.351 | 0.351 |

The Tanimoto distance was computed using molecules generated by each model for each target. Compared with the main text, nvMolkit 0.4.0 was employed here to accelerate the computation.

**Supplementary Table 12.** Two-way ANOVA results for docking scores (Scores) and interaction match rates (Matched) of generated molecules across models and targets.

| Metric | Category | Source | SS | DF | MS | F | p-unc | np2 |
| --- | --- | --- | --- | --- | --- | --- | --- | --- |
| Scores | Dock | Model | 3.322e5 | 14 | 23731.97 | 743.12 | 0 | 0.03 |
|  |  | Target | 2.854e5 | 19 | 15019.27 | 470.30 | 0 | 0.03 |
|  |  | Model * Target | 6.165e5 | 266 | 2317.50 | 72.57 | 0 | 0.06 |
|  |  | Residual | 9.310e6 | 2.915e5 | 31.94 |  |  |  |
|  | Score | Model | 5.708e6 | 9 | 6.342e5 | 6699.74 | 0 | 0.24 |
|  |  | Target | 6.708e6 | 19 | 3.531e5 | 3729.57 | 0 | 0.27 |
|  |  | Model * Target | 2.457e7 | 171 | 1.437e5 | 1517.99 | 0 | 0.57 |
|  |  | Residual | 1.833e7 | 1.936e5 | 94.66 |  |  |  |
| Matched | Dock | Model | 4934.05 | 14 | 352.43 | 3898.15 | 0 | 0.16 |
|  |  | Target | 9873.96 | 19 | 519.68 | 5748.05 | 0 | 0.27 |
|  |  | Model * Target | 4330.80 | 266 | 16.28 | 180.08 | 0 | 0.14 |
|  |  | Residual | 26357.53 | 2.915e5 | 0.09 |  |  |  |
|  | Score | Model | 4219.19 | 9 | 468.80 | 7076.88 | 0 | 0.25 |
|  |  | Target | 6589.12 | 19 | 346.80 | 5235.15 | 0 | 0.34 |
|  |  | Model * Target | 4168.20 | 171 | 24.38 | 367.97 | 0 | 0.25 |
|  |  | Residual | 12826.91 | 1.936e5 | 0.07 |  |  |  |

Two-way Anova was performed with Python package pingouin. **SS**: Sum of Squares, representing the contribution of each factor to the overall variance. **DF**: Degrees of Freedom, indicating the number of independent parameters estimated. **MS**: Mean Square, calculated as SS divided by DF. **F**: F-value, a statistical measure used to test the significance of each factor's effect. **p-unc**: Unadjusted p-value, representing the probability of the effect's significance. **np2**: Partial Eta Squared (η²), measuring effect size and reflecting the proportion of variance explained by the factor.

**Supplementary Table 13.** FCD values of tested models between molecules generated to consistency and specificity test set and their reference compounds.

| Model | 5-HT2A | | BRD4 | | JAK2-TYK2 | | | |
| --- | --- | --- | --- | --- | --- | --- | --- | --- |
|  | holo-apo | Δ_Avg._ | holo-apo | Δ_Avg._ | JAK2-TYK2 | Δ_Avg._ | JAK2-  TYK2ref | TYK2-  JAK2ref |
| DRAGONFLY | 2.466 | 0.143 | 2.527 | 0.082 | 2.498 | 0.111 | 27.342 | 19.520 |
| DeepBlock | 2.172 | 8.129 | 0.881 | 9.420 | 6.927 | 3.374 | 24.549 | 22.527 |
| SimpleSBDD | 0.676 | 0.844 | 0.829 | 0.691 | 1.042 | 0.478 | 39.034 | 32.594 |
| TamGen | - | - | 6.352 | -0.305 | 6.511 | -0.464 | 37.923 | 35.152 |
| DiffSBDD | 4.193 | 1.085 | 5.491 | -0.213 | 2.315 | 2.963 | 31.707 | 26.013 |
| DrugFlow | 4.145 | 14.971 | 4.172 | 14.944 | 15.642 | 3.474 | 39.232 | 42.690 |
| IPDiff | 8.812 | -1.502 | 8.105 | -0.795 | 4.281 | 3.029 | 56.230 | 50.125 |
| Lingo3DMol | 15.155 | 7.911 | 35.993 | -12.927 | 15.577 | 7.489 | 23.783 | 25.162 |
| MolCraft | 0.844 | 4.556 | 4.150 | 1.250 | 2.300 | 3.100 | 38.363 | 34.225 |
| PocketFlow | 2.965 | 3.727 | 4.361 | 2.331 | 14.761 | -8.069 | 43.832 | 37.941 |
| SurfGen | 2.296 | 4.056 | 2.114 | 4.238 | 2.937 | 3.415 | 43.075 | 38.001 |
| TargetDiff | 3.455 | 0.995 | 6.894 | -2.444 | 1.899 | 2.551 | 40.443 | 36.108 |
| DrugFlow-PA | 1.190 | 4.822 | 3.208 | 2.804 | 3.606 | 2.406 | 47.314 | 37.183 |
| MolPilot | 1.003 | 4.443 | 0.728 | 4.718 | 2.459 | 2.987 | 39.616 | 37.055 |
| REINVENT4 | 36.813 | -7.181 | 31.390 | -1.758 | 15.756 | 13.876 | 48.836 | 46.983 |

**ΔAvg.:** The difference between the average pairwise FCD values to the test FCD, which all not ideal values in holo-apo pairs are marked in red. **Note:** TamGen unable to generate molecules in 5-HT2A_AF.

**Supplementary Table 14.** The mean centroid displacement of different sets of generated molecules within the same protein after cross-docking.

| Target | Metric | DRAGONFLY | DeepBlock | DrugFlow | MolCraft | PocketFlow | SurfGen |
| --- | --- | --- | --- | --- | --- | --- | --- |
| 5-HT2A | A-B | 0.063 | 0.141 | 0.720 | 0.942 | 0.293 | 1.171 |
|  | A-C | 2.426 | 2.438 | 2.145 | 1.840 | 1.771 | 3.287 |
|  | B-C | 2.438 | 2.423 | 2.813 | 2.776 | 2.010 | 2.120 |
| 5-HT2A_AF | A-B | 0.072 | 0.099 | 0.745 | 0.448 | 0.880 | 0.379 |
|  | A-C | 5.431 | 5.830 | 6.295 | 5.963 | 5.422 | 5.618 |
|  | B-C | 5.359 | 5.821 | 5.748 | 6.152 | 6.016 | 5.258 |
| BRD4 | A-B | 0.050 | 0.123 | 1.180 | 0.467 | 0.873 | 0.776 |
|  | A-C | 1.153 | 1.217 | 1.012 | 1.059 | 2.086 | 1.328 |
|  | B-C | 1.147 | 1.335 | 1.101 | 1.426 | 1.775 | 1.705 |
| BRD4-apo | A-B | 0.059 | 0.066 | 1.068 | 0.438 | 0.795 | 0.185 |
|  | A-C | 4.426 | 4.306 | 4.702 | 4.133 | 3.806 | 4.988 |
|  | B-C | 4.431 | 4.297 | 5.134 | 4.395 | 4.153 | 5.018 |
| JAK2 | A-B | 0.098 | 0.325 | 0.834 | 0.473 | 0.836 | 0.707 |
|  | A-C | 0.996 | 1.565 | 1.813 | 1.362 | 0.600 | 1.853 |
|  | B-C | 0.973 | 1.881 | 1.285 | 1.803 | 1.374 | 2.411 |
| TYK2 | A-B | 0.075 | 0.160 | 1.593 | 1.879 | 0.735 | 0.742 |
|  | A-C | 2.871 | 2.312 | 1.086 | 1.150 | 2.425 | 2.525 |
|  | B-C | 2.818 | 2.399 | 2.488 | 2.638 | 3.156 | 3.244 |

**A**: the mean centroid of the molecules generated for that protein; **B**: the mean centroid of the molecules generated for the other protein in the protein pair; **C**: the predefined generation center and also the docking grid center of that protein. Units: Å.

**Supplementary Table 15.** Generation batch size or sample size settings for the evaluated models

| Model | Batch Size | Model | Batch Size | Model | Batch Size |
| --- | --- | --- | --- | --- | --- |
| Non-3D | | 3D in-situ | | Optimization | |
| DRAGONFLY | 48 | DiffSBDD | 32 | DrugFlow-PA | 32 |
| DeepBlock | 64 | DrugFlow | 32 | MolPilot | 10 |
| SimpleSBDD | 1200 (sp) | IPDiff | 25 | REINVENT4 | 128 |
| TamGen | 1200 (sp) | Lingo3DMol | 1200 (sp) |  |  |
|  |  | MolCraft | 100 |  |  |
|  |  | PocketFlow | 1200 (sp) |  |  |
|  |  | SurfGen | 1200 (sp) |  |  |
|  |  | TargetDiff | 1200 (sp) |  |  |

*sp* indicates that the model does not have an explicit batch size or does not generate samples in a parallelized manner; therefore, the reported value corresponds directly to sample size.

**Supplementary Table 16.** The predefined key interaction sets for each target.

| Target | H-Bond | HBA | HBD | Halogen bond | Hydrophobic  contact | π-stacking | Salt Bridge  lneg | Salt Bridge  pneg |
| --- | --- | --- | --- | --- | --- | --- | --- | --- |
| 5-HT2A |  |  |  |  | Trp336/Phe339/Phe340 |  |  | Asp155 |
| BCL2 |  |  |  |  | (1) Phe104; (2) Tyr108/Arg146; (3) Phe153; (4) Phe112 |  |  |  |
| BRAF | Cys532 |  |  |  |  |  |  |  |
| BRD4 | Asn140 |  |  |  | (1) Trp81/Pro82/Phe83; (2) Leu92/Leu94/Val87/Tyr97 |  |  |  |
| BTK |  | Met477/Glu475 | Met477 |  |  |  |  |  |
| Beta2AR | (1) Ser203/Ser207; (2) Asn379/Tyr383 |  |  |  |  | Phe357 |  |  |
| DRD2 |  |  |  |  | Trp386 |  |  | Asp114 |
| HDAC6 |  |  |  |  | Phe583/His614 |  |  |  |
| HIV-RT |  |  |  |  | (1) Tyr181/Tyr188/Phe227/Trp229; (2) Leu100/Lys101; (3) Lys103/Val106/Leu234/Pro236/Tyr318 |  |  |  |
| JAK2 |  | Leu932/Glu930 | Leu932 |  |  |  |  |  |
| MEK1 | Ser212 |  |  | Val127 |  |  |  |  |
| NAMPT | Ser275/Asp219 |  |  |  | Val242 | Phe193+Tyr18' |  |  |
| PI3Kα | Val851 |  |  |  | Tyr836/Ile932 |  |  |  |
| PPARα | Ser280 |  |  |  |  |  | His440 |  |
| PRMT5 | (1) Lys333/Glu444/Glu435; (2) Glu312/Ser578/Phe580 |  |  |  |  | Phe327/Trp579 |  |  |
| ROCK1 | Met156 |  |  |  |  |  |  |  |
| RXRα | Ala327 |  |  |  |  |  | Arg316 |  |
| TYK2 | (1) Val690; (2) Lys642/Glu688 |  |  |  |  |  |  |  |

**Note:** *lneg/pneg* indicate that the protein or ligand carries a negative charge. In the interaction set descriptions, a forward slash (/) denotes “or”, and a plus sign (+) denotes “and”. In NAMPT, Tyr18’ indicates that this residue belongs to the other chain.

# 2 Supplementary Figures and Preliminary Results


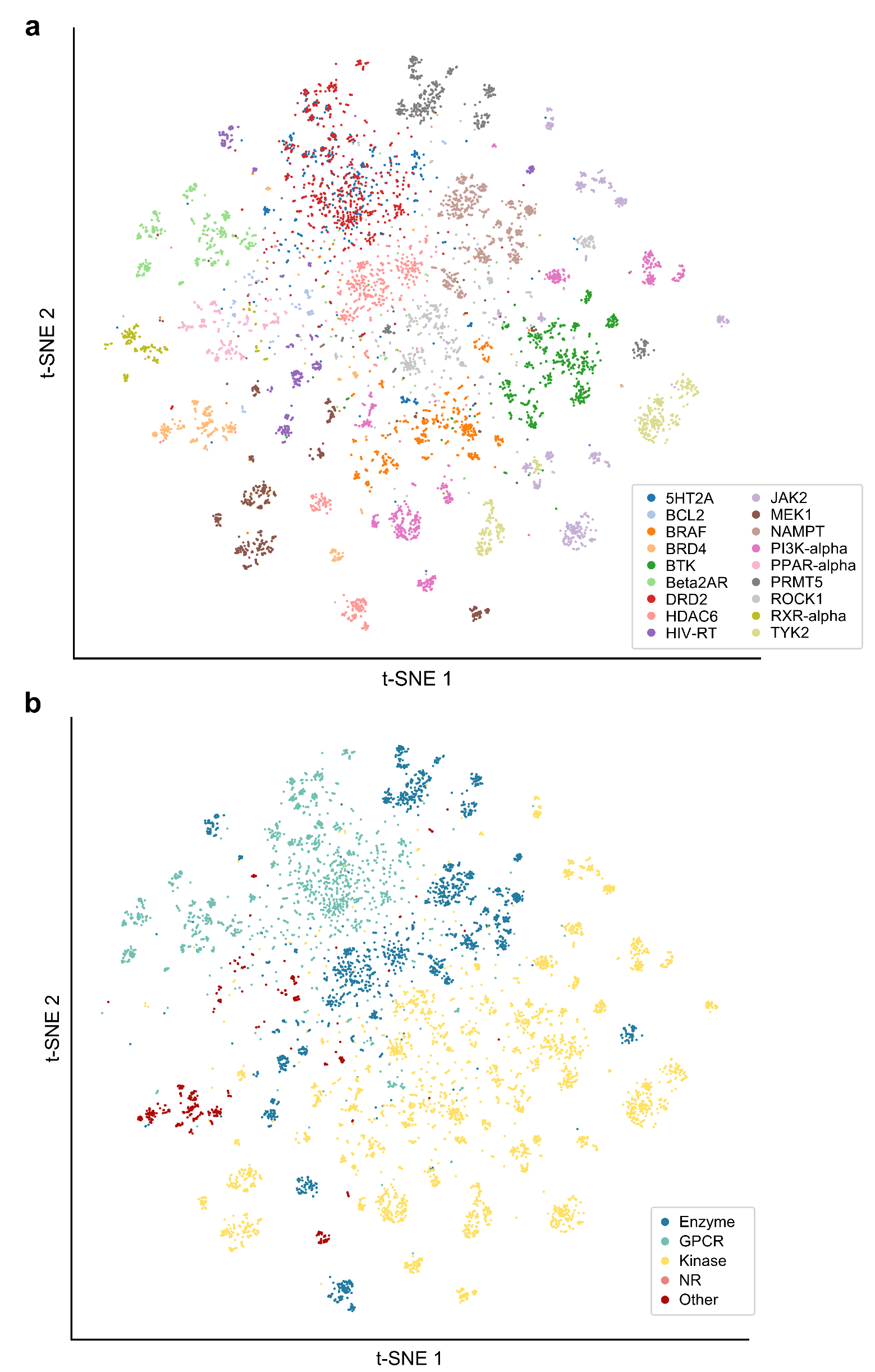


**Supplementary Figure 1.** t-SNE projection of active reference compounds based on ECFP4 fingerprints, grouped by **(a)** target and **(b)** protein family classification.


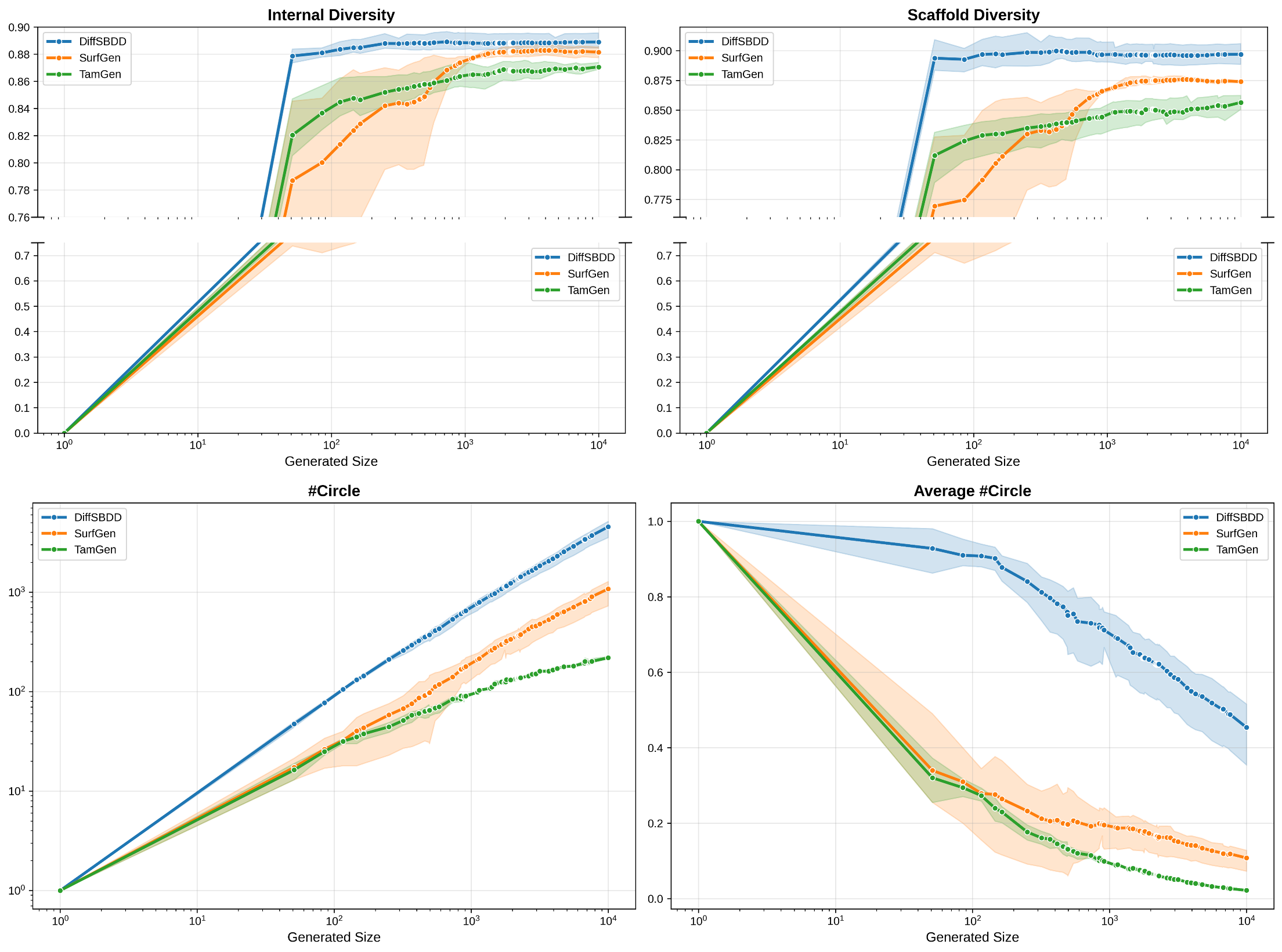
**Supplementary Figure 2.** Trends in diversity metrics with increasing numbers of generated molecules.

Inspired by the work of Özçelik et al.[1](#_ENREF_1), we selected three models to generate molecules in targets from three different protein families and examined the convergence range of diversity. The maximum number of generated molecules was set to 10,000 due to computational constraints. Two diversity metrics were used: internal diversity (molecules and scaffolds) and #Circle (and the average contribution per molecule, Average #Circle). For IntDiv, both the DiffSBDD and TamGen converged by around 100 molecules, while SurfGen at around 1,000 molecules. For #Circle, since this metric increases with sample size[2](#_ENREF_2), Average #Circle provides a more meaningful assessment. Except for DiffSBDD, Average #Circle stabilized after about 1,000 molecules, suggesting that the incremental contribution of new molecules to overall diversity diminished and that the chemical space distribution reached a stable boundary. Balancing computational cost and diversity outcomes, we selected 1,000 molecules as the sampling size for testing.

**
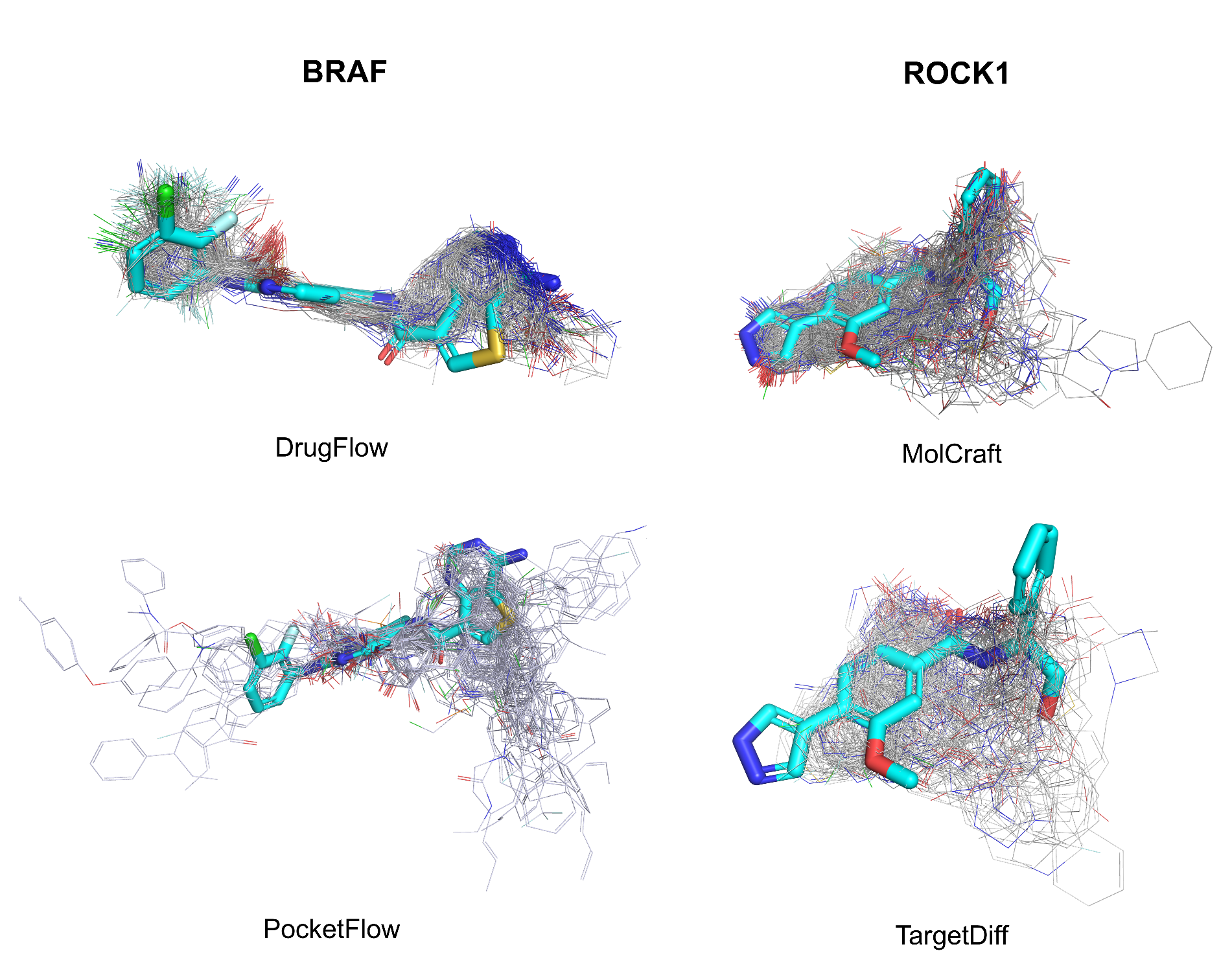
**

**Supplementary Figure 3.** The origin ligand of the target (light blue sticks) and the generated molecules for that target by the model (lines).

The molecules generated by DrugFlow and MolCraft exhibited a high degree of spatial overlap with the original ligands, particularly in the case of DrugFlow. In the pseudo-ligand generation test shown in Supplementary Figure 4, DrugFlow even failed to generate any molecules.

**
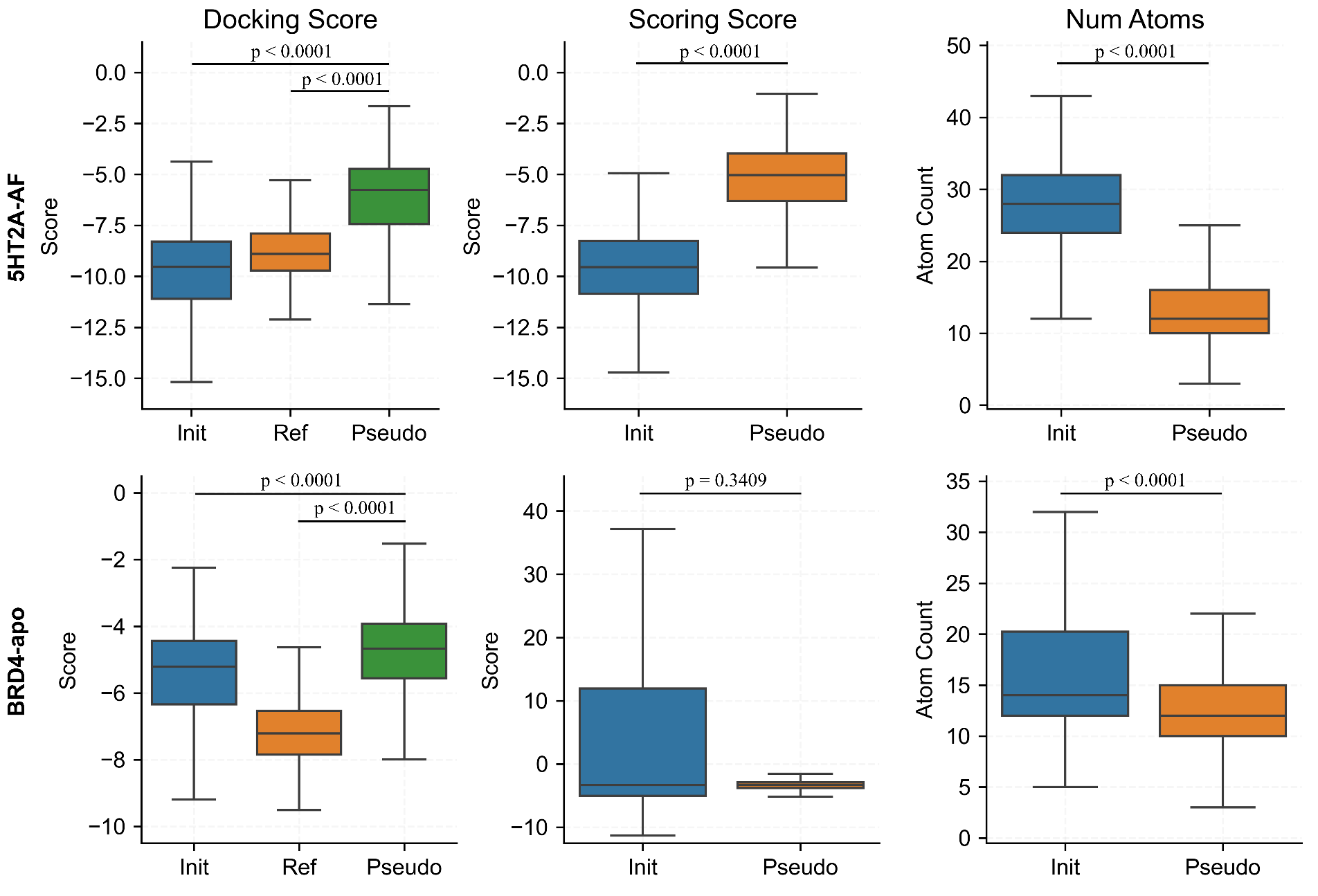
**

**Supplementary Figure 4.** Boxplots of distributions of docking scores, scoring scores, and molecular weight of generated molecules (n=1000) sampling a pseudo-ligand (Pseudo) consisting of a single atom in two ligand-free structures with MolCraft, compared with generation results with sampling the original ligand (Init), and the reference molecules (Ref). Significance tests were analyzed with a two-sided Mann-Whitney U test.

The results show that DrugFlow completely failed to generate molecules from the pseudo-ligand, while MolCraft’s generation quality markedly declined (Supplementary Figure 4). In both targets, the generated molecules contained markedly fewer atoms, with a median of around 11. This reduction was especially pronounced in 5-HT2A_AF, where the median atom count decreased by 15 compared with generations in the initial ligand, whereas in BRD4-apo the reduction was only a few atoms. Key interaction recovery also varied sharply. In BRD4-apo, the exact match rate of initial poses even dropped more than 60-fold (0.501 vs. 0.008), indicating that MolCraft’s performance strongly depends on sampling around the reference ligand.


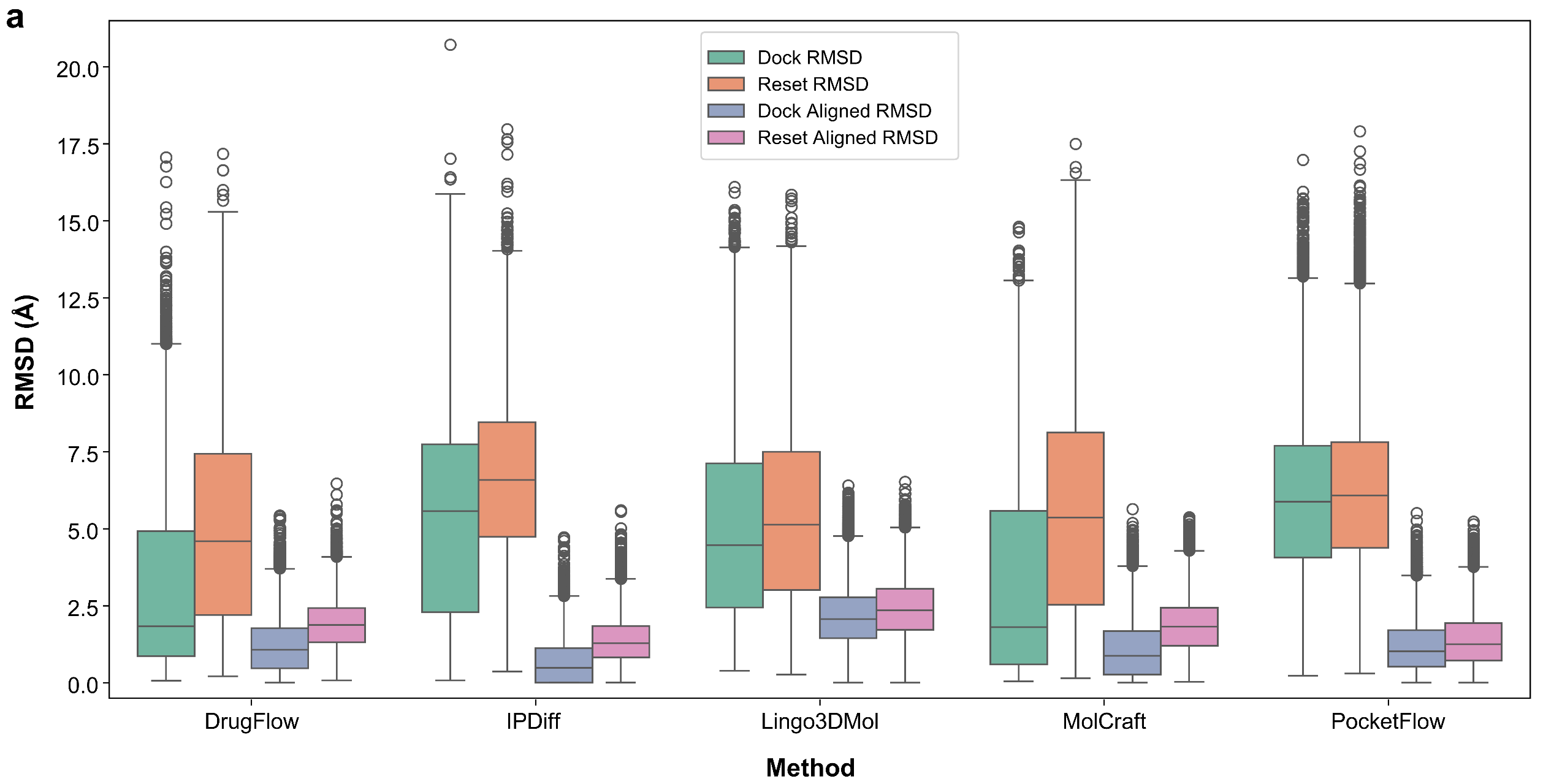


**Supplementary Figure 5.** Boxplots showing the distributions of coordinate and aligned RMSD for selected 3D *in-situ* models after redocking and conformation-reset docking, relative to their initial poses. Few outliers with RMSD values >25 Å are excluded from the figure.

**
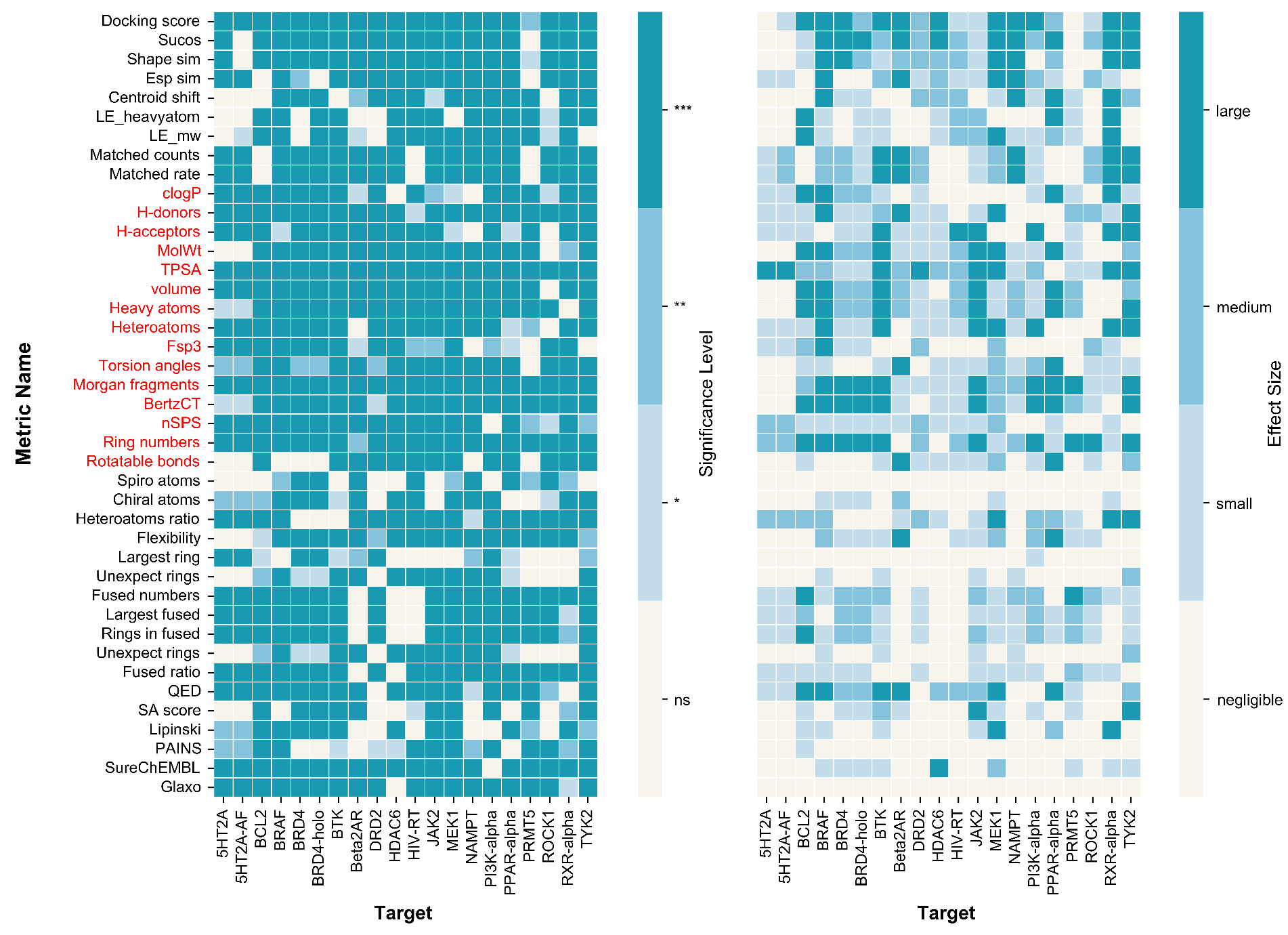
**

**Supplementary Figure 6.** Significance analysis of affinities, interactions, physicochemical properties, structural features, and drug-likeness descriptors between reference and random molecules across all targets in TarPass. Descriptors highlighted in red showed significant differences with large effect sizes across most targets and were therefore selected for calculating the Wasserstein distance based on similarity. Significance was assessed using a two-sided Mann-Whitney U test. Significance Level: ns *p* ≥ 0.05, **p* < 0.05, ***p* < 0.01, ****p* < 0.001. Effect sizes were calculated using Cliff’s delta, with thresholds defined as negligible < 0.147, small < 0.33, medium < 0.474, and large > 0.474


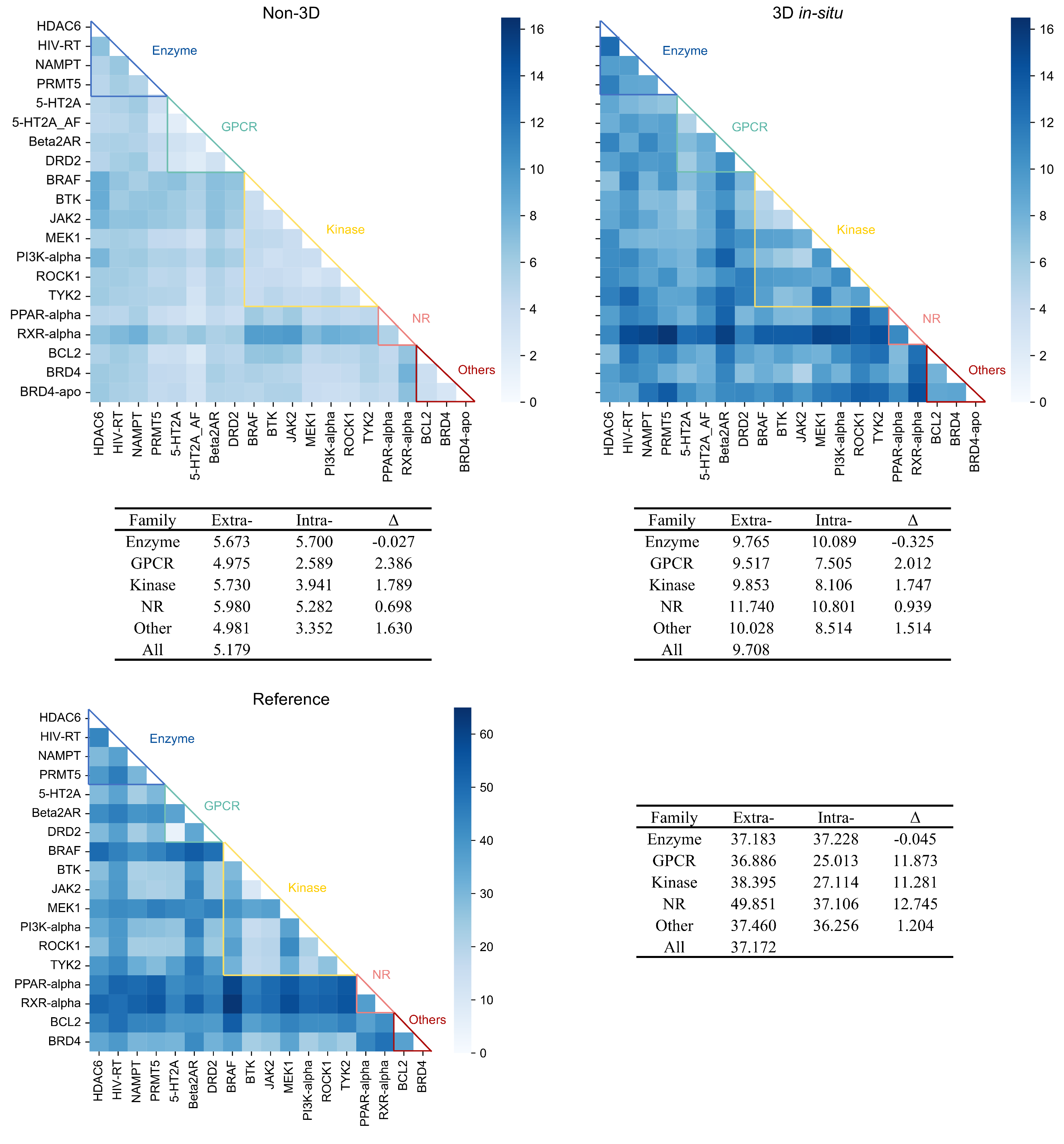


**Supplementary Figure 7.** Heatmaps of average pairwise FCD values among all targets in non-3D, 3D *in-situ* paradigms and reference molecules. Tables around each subplot show the detail value of extra- and intra-family FCD values and their difference.


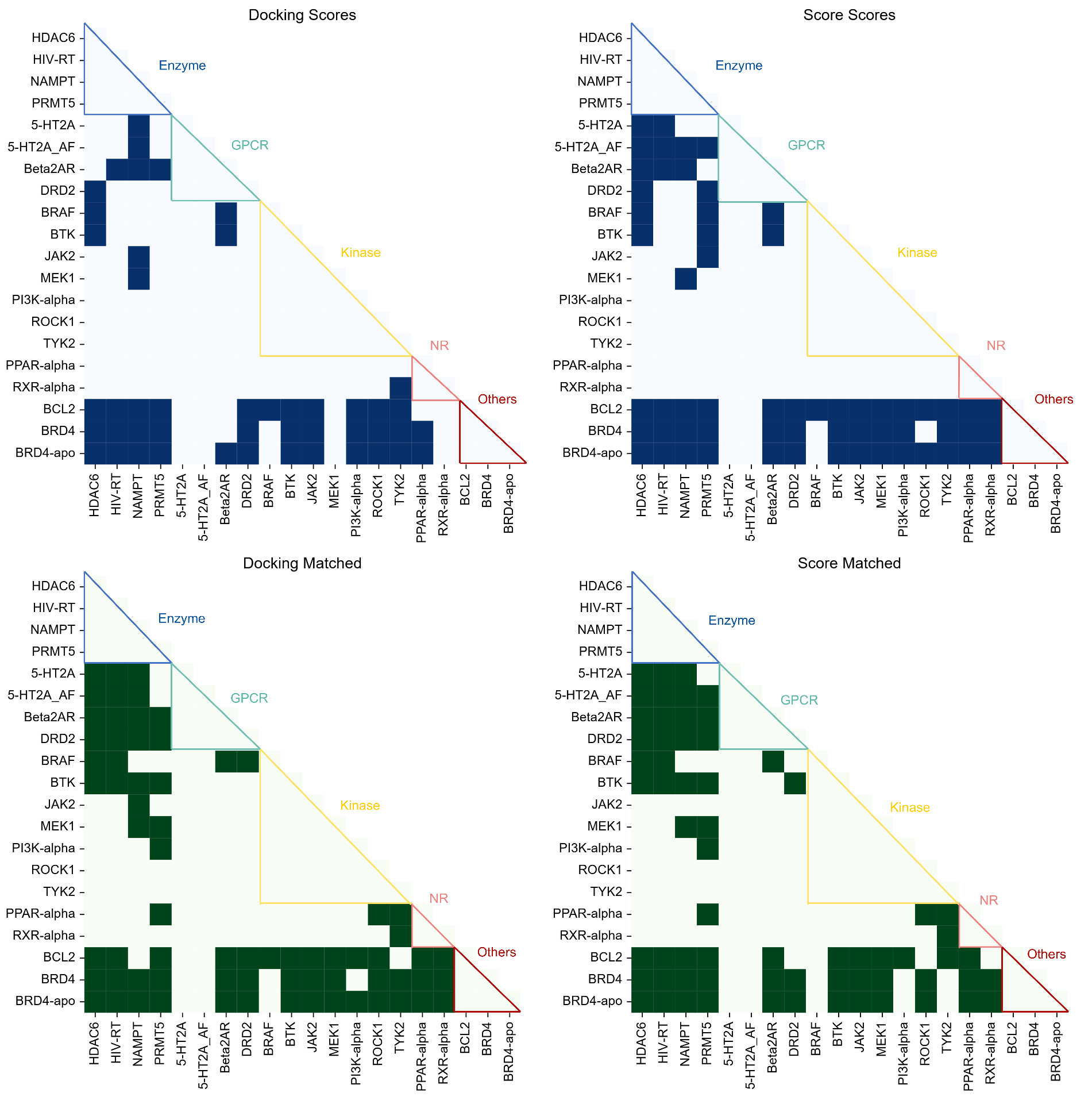


**Supplementary Figure 8.** Significance heatmaps of docking score (scores) and interaction match rate (matched) from redocking and rescoring results. The significance determination was based on the results of the two-way ANOVA (Supplementary Table 11). Pairwise Games-Howell post hoc tests were performed for targets with Python packages pingouin, followed by multiple comparison correction. Significance was defined using a dual criterion: adjusted *p*-values < 0.05 and absolute value of Hedges’ g effect size greater than 0.2[3](#_ENREF_3).

# 3 Details of Evaluation Metrics

In the supplementary tables, each metric ($M$) reported at the model or method level was calculated using the following equation:

$$\begin{aligned} M=\frac{1}{T}\sum_{i=1}^{T} (\frac{1}{N}\sum_{j=1}^{N} v_{ij}) \#\left( S1 \right) \end{aligned}$$

Where, $T$ is the number of tested targets, $N$ is the number of generated molecules to the corresponding target, $v$ is the metric value to the single molecule, if the metric is Boolean, then the value is either 0 (False) or 1 (True).

The equation indicates that each metric was first averaged within each target, followed by computing the mean across targets. However, for docking scores, the median and Q1 (top25%) values within each target was used instead, and then the mean across targets was calculated.

## 3.1 Binding Affinity and Interactions

**Vina Score**: Docking score obtained with Gnina.

**Ligand efficiency (LE)**: A ligand efficiency metric calculated from a docking score, obtained by normalizing the predicted binding affinity (or docking score) by the number of heavy atoms in the ligand. It reflects the average contribution of each non-hydrogen atom to the predicted binding interaction.

$$\begin{aligned} \mathrm{LE}\boldsymbol{=}\frac{\left| Docking Score \right|}{N_{heavy atom}} \mathrm{if}Docking Score <0 else NaN\#\left( S2 \right) \end{aligned}$$

**Exact Match:** A Boolean value indicating whether the predefined set of key interactions is fully contained within the detected interaction set.

**Match Ratio:** A Boolean value indicating whether any of the predefined key interactions are present in the detected interaction set.

**Detected Interactions:** The total number of interactions detected using PLIP.

## 3.2 Binding modes

**Root Mean Square Deviation (RMSD)**: If a reference ligand pose exists, RMSD is defined as the square root of the average of the squared distances between the corresponding heavy atoms (non-hydrogen) of the docked ligand pose and the reference pose, after optimal superposition of the two structures.

$$\begin{aligned} \mathrm{RMSD}=\sqrt{\frac{1}{N}\sum_{i} \parallel x_{i}^{dock}-x_{i}^{ref}\parallel^{2}} \#\left( S3 \right) \end{aligned}$$

**No Clashes**: A Boolean quality filter indicating whether a docked molecular pose is free from severe steric clashes, defined as nonbonded interatomic distances being no less than a specified threshold (typically the sum of the atoms’ van der Waals radii minus a small tolerance, e.g., 0.4 Å). Poses with atom-atom overlaps beyond the threshold are marked as having clashes.

**SuCOS**: SuCOS is a combined score that mixes a shape (volume/surface) overlap term and a chemical‑feature (pharmacophore) overlap term into a single scalar that reflects both geometric and feature agreement between two conformations. Typical implementations composed of half shape overlap (shape protrude similarity) and half chemical feature overlap[4](#_ENREF_4).

$$\begin{aligned} \mathrm{SuCOS}=0.5\left( 1-ShapeProtrudeDist \right)+0.5\left( ScoreFeats \right) \#\left( S4 \right) \end{aligned}$$

**Shape Sim.**: Shape similarity quantifies how much the 3D volumes or Gaussian atom densities of two conformers (A & B in Eq. S5) overlap ($O$) after optimal rigid alignment. Volume Tanimoto used as the normalized measure for this work.

$$\begin{aligned} S_{\text{Tanimoto}}=\frac{O}{V_{A} + V_{B} - O}\quad\in\left[ 0,1 \right]\#\left( S5 \right) \end{aligned}$$

**ESP Sim.** (electrostatic potential similarity): ESP similarity compares the electrostatic potential distributions of two molecules in space[5](#_ENREF_5). To calculate EPS similarity, it will firstly generate 3D conformations for the two molecules and assign consistent partial charges (e.g., RESP, AM1‑BCC, or Gasteiger, we used MMFF[6](#_ENREF_6)), then compute each molecule’s electrostatic potential φ(r) at position $\boldsymbol{r}$ as the Coulomb sum of atomic charges (Eq. S6) with analytical Gaussian fitting method as default which approximate each potential with a sum of three Gaussian functions and integrate the fit. After rigidly aligning the molecules, compute the normalized overlap integral of their potentials with Carbo-type similarity (Eq. S7).

$$\begin{aligned} \phi\left( \boldsymbol{r} \right)=\sum_{i} \frac{q_{i}}{4\pi\varepsilon_{0}\left| \boldsymbol{r}-r_{i} \right|}\#\left( S6 \right) \end{aligned}$$

$$\begin{aligned} \text{ESP Sim}\left( A,B \right)=\frac{\int\phi_{A}\left( \mathbf{r} \right)\phi_{B}\left( \mathbf{r} \right)d\mathbf{r}}{\sqrt{\int[\phi_{A}(\mathbf{r})]^{2}d\mathbf{r}\text{ }\int[\phi_{B}(\mathbf{r})]^{2}d\mathbf{r}}}\#\left( S7 \right) \end{aligned}$$

**Centroid shift**: Distance between the centroid of the docked ligand and reference centroid (if reference ligand is provided). Used as a proxy for binding pose displacement.

$$\begin{aligned} d=\parallel C_{\mathrm{dock}}-C_{\mathrm{ref}}\parallel\#\left( S8 \right) \end{aligned}$$

## 3.3 Structural Properties

**Csp3 fraction (Fsp3)**: The ratio of the number of sp³-hybridized carbon atoms to the total number of carbon atoms in a molecule. It is calculated as:

$$\begin{aligned} Fsp3 = \frac{N_{Csp3}}{N_{C}} \#\left( S9 \right) \end{aligned}$$

This parameter reflects molecular saturation and structural complexity, with higher values often associated with improved drug-like properties such as solubility. [7](#_ENREF_7)

**Chiral atoms:** The number of chiral centers in the molecule, including unassigned centers.

**Torsion angle** (Dihedral angle): The angle between two planes formed by four connected atoms (A-B-C-D), measured by the rotation around the B-C bond. It quantifies the conformational state of the molecule.[8](#_ENREF_8)

**Rotatable bonds**: The number of freely rotatable single bonds, generally excluding terminal bonds, amide C–N bonds, or bonds within rigid rings.[9](#_ENREF_9)

**Morgan fragments:** Substructural fragments generated using the Morgan fingerprint algorithm, which encodes atom environments iteratively within a specified radius.[10](#_ENREF_10)

**BertzCT** (Bertz Chemical Complexity Index) is a unitless numerical descriptor that quantifies the topological complexity of a molecule, where topological complexity refers to the degree of structural intricacy in a molecule’s connectivity graph, represented with atoms as vertices and bonds as edges. This complexity arises from factors such as branching, the presence and interconnection of ring systems, variations in atom degrees, and the degree of molecular symmetry, with less symmetric and more irregular structures being considered more complex[11](#_ENREF_11).

**nSPS** (normalized Spacial Score): The Spacial Score divided by the number of heavy atoms in the molecule, providing a size-independent measure of spatial complexity and three-dimensionality,[12](#_ENREF_12) which originated from the Spacial Score (SPS) , an empirical scoring system proposed to uniformly express the spacial complexity of small molecules on a highly granular scale. It builds upon the underlying principles of two widely used molecular complexity indicators: the fraction of sp3-hybridized carbons and the fraction of stereogenic carbons, while overcoming the limitations of their simplistic nature in capturing comprehensive molecular topology.

**Ring numbers:** The number of rings in the molecular graph, determined by cycle detection algorithms (e.g., cyclomatic number or smallest set of smallest rings).

**All common elements**: A Boolean descriptor that returns True if a molecule contains only the following elements: carbon (C), hydrogen (H), nitrogen (N), oxygen (O), fluorine (F), chlorine (Cl), bromine (Br), iodine (I), phosphorus (P), sulfur (S).

**Completeness**: Whether the molecule is a single connected component (no disconnected fragments).

**Flexibility:** A custom ratio reflecting molecular flexibility, defined as the number of rotatable bonds normalized by estimated rigid bonds.

$$\begin{aligned} \text{Flexibility}=\frac{N_{\text{rotatable bonds}}}{max\left( 1,\left( N_{\text{heavy atoms}}-1 \right)-N_{\text{rotatable bonds}} \right)} \#\left( S10 \right) \end{aligned}$$

## 3.4 Drug-likeness

**QED** (Quantitative Estimate of Drug-likeness): A numerical score between 0 and 1 quantifying how “drug-like” a molecule is, derived by fitting desirability functions to the empirical distributions of eight physicochemical properties observed in orally marketed drugs: molecular weight, octanol-water partition coefficient (LogP), topological polar surface area (TPSA), number of hydrogen bond donors (HBD), number of hydrogen bond acceptors (HBA), number of rotatable bonds (RB), number of aromatic rings, and the presence of structural alerts. The final QED score is computed as the weighted geometric mean of the desirability values for these properties.[13](#_ENREF_13)

**SA Score** (Synthetic Accessibility Score): A heuristic molecular descriptor that estimates the ease of chemical synthesis for a given compound[14](#_ENREF_14). The score is calculated as the sum of fragment contributions—derived from the occurrence frequency of molecular fragments in large, synthesizable compound databases—and complexity penalties, which account for structure features such as rare ring systems, stereocenters, and unusual atom types.

**Lipinski (Rule-of-Five)**: A set of empirical guidelines proposed by Christopher A. Lipinski in 1997 to evaluate the drug-likeness of a compound, particularly its potential for good oral bioavailability in humans. According to the rule, poor absorption or permeation is more likely when a molecule violates two or more of the following criteria:

1. Molecular weight ≤ 500 Daltons;
2. LogP (octanol–water partition coefficient) ≤ 5;
3. Number of hydrogen bond donors (sum of OH and NH groups) ≤ 5;
4. Number of hydrogen bond acceptors (sum of N and O atoms) ≤ 10;
5. Rotatable bonds ≤ 10.

## 3.5 Structural Alerts

**PAINS** (Pan-Assay Interference Compounds): A set of structural motifs known to produce frequent false positives across multiple biological assays, due to non-specific mechanisms such as covalent modification of targets, redox cycling, or assay interference. In cheminformatics, PAINS filters detect whether a given molecule contains any of these substructures, typically returning a boolean flag. The concept and associated SMARTS patterns were defined by Baell and Holloway (2010) for use in screening library curation[15](#_ENREF_15).

**SureChEMBL**: A binary descriptor indicating whether a molecule contains any substructure patterns listed in the SureChEMBL structural alert set. These alerts are curated SMARTS patterns derived from the ChEMBL database, chemical patent literature, and medicinal chemistry sources, representing problematic moieties such as toxicophores, reactive functional groups, or common assay interference motifs.[16](#_ENREF_16)

**Glaxo** (GSK structural alerts): A boolean descriptor indicating whether the molecule contains chemical substructures listed in the GlaxoSmithKline (GSK) structural alert catalog. These alerts correspond to functional groups considered unfavorable in drug development due to potential toxicity, chemical instability, or metabolic liabilities.

## 3.6 Physicochemical Properties

**cLogP:** cLogP values are calculated 1-octanol/water partition coefficients.[8](#_ENREF_8) Typically estimated by fragmental or atom-based contribution methods, summing empirical contributions of atoms or fragments.

**H-donors (HBD)**: The number of hydrogen bond donors in a molecule.

**H-acceptors (HBA):** The number of hydrogen bond acceptors in a molecule.

**MolWt (Molecular weight)**: Ratio of the mass of a molecule to the unified atomic mass unit. Sometimes called the molecular weight or relative molar mass.[8](#_ENREF_8)

**Topological Polar Surface Area** (**TPSA**): The sum of the surface areas of polar atoms in a molecule, computed solely from the two‑dimensional topology (graph) of the molecule without the need for 3D coordinates, typically using fragment‑based contributions derived from a training set.[17](#_ENREF_17)

**Volume (Molecular volume):** The spatial volume occupied by a molecule, which can be computed from its 3D conformation using methods such as van der Waals volume or solvent-excluded volume calculations, or approximated via empirical formulas that sum predefined atomic and bond contributions; the specific approach varies depending on the software implementation.

**Heavy atoms:** The total number of atoms in a molecule excluding hydrogen atoms[18](#_ENREF_18).

**Heteroatoms:** Atoms in an organic molecule that are neither carbon nor hydrogen. Typical heteroatoms include nitrogen (N), oxygen (O), sulfur (S), phosphorus (P), and the halogens (F, Cl, Br, I).

## 3.7 Chemical Distances

**Internal Diversity (IntDiv)**: IntDiv measures how diverse the generated molecules are with respect to each other, typically using pairwise Tanimoto distances based on molecular fingerprints (e.g., ECFP4). Higher values indicate greater structural diversity within the generated set.

$$\begin{aligned} \text{IntDiv} = 1 - \frac{2}{N\left( N - 1 \right)} \sum_{i < j} T\left( x_{i}, x_{j} \right) \#\left( S11 \right) \end{aligned}$$

where $T(x_{i},x_{j})$is the Tanimoto similarity between molecules $x_{i}$and $x_{j}$, and $N$is the number of generated molecules.

**#Circle**: #Circle is a locality-based chemical space coverage measure[2](#_ENREF_2). Described as:

$$\begin{aligned} \#Circles \left( \mathcal{S;}d,t \right)=\max_{\mathcal{C\subseteq S}} \left| \mathcal{C} \right|\text{ s.t. }d\left( x,y \right)>t, \forall x\neq y\in\mathcal{C \#}\left( S12 \right) \end{aligned}$$

Where $t$ is a distance threshold set as 0.75. $\mathcal{C}$ is a subset of $\mathcal{S}$, containing a specific molecule x and its non-neighboring molecules.

**Fréchet ChemNet Distance (FCD)**: FCD evaluates how close the distribution of generated molecules is to that of real (reference) molecules. It’s analogous to the Fréchet Inception Distance in image generation, but uses molecular embeddings from a pretrained neural network ChemNet. Lower FCD values indicate higher fidelity of chemical and biology features to the reference chemical space[19](#_ENREF_19).

$$\begin{aligned} \text{FCD}=|\mu_{g}-\mu_{r}|_{2}^{2}+\text{Tr}\left( \Sigma_{g}+\Sigma_{r}-2\left( \Sigma_{g}\Sigma_{r} \right)^{1/2} \right)\#\left( S13 \right) \end{aligned}$$

where $(\mu_{g},\Sigma_{g})$and $(\mu_{r},\Sigma_{r})$are the mean and covariance of the generated and reference molecule embeddings, respectively.

**Wasserstein distance**: The Wasserstein distance measures the minimum cost of transforming one probability distribution into another, where “cost” is defined by how far probability mass must be moved.

$$\begin{aligned} W\left( P,Q \right)=\inf_{\gamma\in\Pi\left( P,Q \right)} E_{\left( x,y \right)\sim\gamma}\left[ ||x-y|| \right]\#\left( S14 \right) \end{aligned}$$

Where$P$and $Q$are two probability distributions,$\Pi(P,Q)$is the set of all joint distributions with marginals $P$and $Q$, $\gamma(x,y)$describes how much probability mass is transported from $x$to $y$, and $\parallel x-y\parallel$is the cost (typically Euclidean distance).

# 4 Targets and Interactions Information

## 4.1 Enzymes

- **HDAC6** Key interaction(s): Hydrophobic contacts to Phe583 **or** Phe643.

HDAC6 is a member of the histone deacetylase (HDAC) family and is considered a potential therapeutic target for cancer treatment. In this study, the selected HDAC6 structure is derived from *Danio rerio* (zebrafish) rather than from humans, which exhibits high sequence and structural homology with the human protein. The ligand-binding pocket used in this study corresponds to the CD2 catalytic domain of HDAC6, with the bound ligand being an inhibitor.

HDAC6 inhibitors can generally be described by three pharmacophoric components: a zinc-binding group that coordinates with the catalytic zinc ion, a linker that traverses the hydrophobic channel, and a terminal cap group[20-23](#_ENREF_20). Due to current technical limitations, metal-associated interactions were not considered, and the zinc coordination was therefore excluded. Among the remaining pharmacophoric features, the linker region is highly conserved, which typically consists of an aliphatic chain or a large, rigid aromatic moiety[20](#_ENREF_20). Phe583 and Phe643 were selected as key residues because they are located on opposite sides of the hydrophobic pocket, flanking the ligand[21](#_ENREF_21)^,^[23-25](#_ENREF_23). From a conservative perspective, the presence of a hydrophobic interaction (including π-stacking as categorized by PLIP) with either of these residues was considered to satisfy the pharmacophore criterion.

- **HIV-RT** Key interactions: Hydrophobic contacts to (1) Tyr181 **or** Tyr188 **or** Phe227 **or** Trp229; (2) Leu100 **or** Lys101; and (3) Lys103 **or** Val106 **or** Leu234 **or** Pro236 **or** Tyr318.

HIV reverse transcriptase (RT) is an essential enzyme in the viral replication cycle and serves as a major target for anti-HIV therapy, as it has no homolog in eukaryotic cells[26](#_ENREF_26). In this study, the binding pocket used corresponds to the non-nucleoside inhibitor binding pocket (NNIBP), which accommodates non-nucleoside reverse transcriptase inhibitors (NNRTIs). This pocket is located approximately 10 Å away from the polymerase active site and represents a highly hydrophobic allosteric site[27](#_ENREF_27).

NNRTIs typically exhibit high conformational flexibility, enabling them to fit into the horseshoe-shaped NNIBP, which can be divided into three subregions: the tunnel, entrance, and groove, each associated with specific pharmacophoric interactions[27](#_ENREF_27)^,^[28](#_ENREF_28). In the tunnel region, Tyr181, Tyr188, Phe227, and Trp229 are key residues commonly involved in hydrophobic contacts. The entrance region primarily involves Leu100 or Lys101, while the groove encompasses several residues, including Lys103, Val106, Leu234, Pro236, and Tyr318[28](#_ENREF_28)^,^[29](#_ENREF_29).

- **NAMPT** Key interactions: (1) Hydrogen bonds to Ser275 **or** Asp219; (2) Hydrophobic contacts to Val242; (3) π-stackings to Phe193 **and** Tyr18' (chain B).

Nicotinamide phosphoribosyltransferase (NAMPT) is a key enzyme involved in the biosynthesis of nicotinamide adenine dinucleotide (NAD) and has been found to be overexpressed in various tumor cells[30](#_ENREF_30). In this study, the selected binding pocket corresponds to the active catalytic site, where the co-crystallized ligand is an inhibitor.

NAMPT functions as a homodimer, with its binding pocket located at the protein–protein interface between the two monomers[31](#_ENREF_31). Under physiological conditions, both the natural substrate nicotinamide (NM) and the product nicotinamide mononucleotide (NMN) must simultaneously form π–π stacking interactions with two stacked aromatic residues: Phe193 from one monomer and Tyr18′ from the other monomer[31](#_ENREF_31). These constitute the most critical interaction group. Because the pocket exhibits a long, tunnel-shaped topology, additional interactions beyond the innermost aromatic pair are typically observed. Within the tunnel, NMN and other inhibitors often form hydrogen bonds with Ser275 and/or Asp219[31-33](#_ENREF_31). Val242, positioned closer to the outer region of the pocket, frequently engages in hydrophobic contacts with inhibitors, indicating that the ligand occupies a substantial portion of the binding cavity. Consequently, Val242 is also considered a key residue in the interaction set[33](#_ENREF_33).

- **PRMT5** Key interactions: Hydrogen bonds to (1) Lys333 **or** Glu444 **or** Glu435; (2) Glu312 **or** Ser578 **or** Phe580; (3) π-stackings to Phe327 **or** Trp579.

PRMT5 belongs to the type II family of protein arginine methyltransferases (PRMTs), which use S-adenosylmethionine (SAM) as a methyl donor to catalyze highly specific symmetric dimethylation of arginine residues[34](#_ENREF_34). Dysregulation of PRMT5 activity has been widely observed in multiple cancers and other diseases, making it an important therapeutic target. First-generation PRMT5 inhibitors act through direct inhibition of PRMT5, which also suppresses in healthy tissues, leading to toxicity and a narrow therapeutic window[35](#_ENREF_35). Subsequent studies revealed that, deletion of the tumor suppressor gene CDKN2A is often accompanied by the co-deletion of the adjacent MTAP gene[36](#_ENREF_36). Loss of MTAP results in the accumulation of its substrate, methylthioadenosine (MTA), a weak natural inhibitor of PRMT5 that competes with SAM for binding at the cofactor site, forming a PRMT5/MTA complex. This weak inhibition increases the dependence of cancer cells on PRMT5, rendering them highly sensitive to PRMT5 inhibition[36](#_ENREF_36). Consequently, selective inhibition of the PRMT5/MTA complex represents a synthetic lethal strategy with high tumor specificity.

In this study, the employed structure corresponds to the PRMT5/MTA complex, with the co-crystallized ligand being an MTA-cooperative inhibitor. Researchers typically use a histone H4 peptide bound to the PRMT5/MTA complex to model substrate engagement[34](#_ENREF_34)^,^[36](#_ENREF_36). MTA-cooperative inhibitors generally occupy the binding site of the SGRG motif within the H4 peptide, particularly extending into the narrow arginine-binding tunnel formed by Leu312, Phe327, and Trp579, which constitutes the SAM-binding catalytic site[34](#_ENREF_34). Therefore, our focus is on the key interactions within this pocket. Within the arginine-binding cavity, the aromatic scaffold of the ligand is sandwiched between the side chains of Phe327 and Trp579 through π–π stacking[37](#_ENREF_37)^,^[38](#_ENREF_38); according to the conservative criteria adopted in PLIP analysis, interaction with either residue is considered valid. In the region closer to MTA, residues such as Lys333, Glu444, or Glu435 frequently participate in hydrogen bonding interactions[35](#_ENREF_35)^,^[39](#_ENREF_39). Additionally, residues located toward the outer side of the pocket including Glu312, Ser578, and Phe580, which also commonly engage in hydrogen bonding, contributing to ligand stabilization[39](#_ENREF_39)^,^[40](#_ENREF_40).

## 4.2 GPCRs

- **5-HT2A** Key interactions: Hydrophobic contacts to (1) Trp336 **or** Phe339 **or** Phe340; (2) Salt bridge (protein negative) to Asp155.

Serotonin, also known as 5-hydroxytryptamine (5-HT), is a crucial neurotransmitter involved in numerous physiological and neurological processes. A wide range of drugs targeting serotonin receptors are currently used in the treatment of psychiatric and neurological disorders. In this study, we selected the inhibitor-bound structure of the 5-HT2A receptor in inactive states and an *apo*-structure predicted by AlphaFold.

5-HT, as the endogenous ligand of 5-HT2A, its terminal amine forms a salt bridge with Asp155^3.32^, which constitutes the most critical interaction within the orthosteric binding pocket[41](#_ENREF_41). This salt bridge is a hallmark feature that can be consistently observed among ligands exhibiting diverse pharmacological profiles including agonists, antagonists, and partial agonists[42-45](#_ENREF_42). For inhibitors in particular, contact with the “toggle switch” residue Trp336^6.48^ is especially important: hydrophobic contacts or π–π stackings with this residue help to stabilize the receptor in its inactive conformation[41](#_ENREF_41)^,^[46](#_ENREF_46). In addition, hydrophobic contacts between Phe339 and Phe340, residues located near the toggle switch, are highly conserved, and site-directed mutagenesis studies have confirmed their critical roles in ligand binding[41](#_ENREF_41)^,^[43](#_ENREF_43)^,^[44](#_ENREF_44). From a conservative standpoint, any hydrophobic interaction involving these residues is considered acceptable.

- **Beta2AR** Key interactions: Hydrogen bonds to (1) Ser203 **or** Ser207; Asn379 **or** Tyr383; (3) π-stackings to Phe357.

The β_2_-adrenergic receptor (Beta2AR) is a prototypical class A GPCR and a well-established drug target. In this study, we used the cryo-EM structure of Beta2AR in complex with olodaterol, a clinically approved full agonist, as the representative model structure. This construct is an engineered fusion protein, and therefore its amino acid numbering differs slightly from that of the wild-type receptor[47](#_ENREF_47).

A key conserved residue, Phe^6.52^ (corresponding to residue 290 in most structures, or 357 in current structure), plays a crucial role in binding both the endogenous ligand epinephrine and other β-phenylethylamine based adrenergic agonists by forming a π–π stacking interaction with the aromatic ring of the ligand’s core scaffold. This interaction is consistently observed across ligands with diverse pharmacological effects, including agonists and antagonists[47-50](#_ENREF_47). In addition, within the β-phenylethylamine scaffold, the two serine residues Ser203^5.42^ and Ser207^5.46^ often form hydrogen bonds with the catechol hydroxyl groups of epinephrine; in certain antagonist-bound structures, however, only one of these interactions is maintained. At the ligand’s tail, the alkanolamine moiety typically forms a hydrogen bond with either Asn^7.39^ or Tyr^7.43^ (corresponding to residues 312/316 in the wild type, or 379/383 in the current structure) [47-50](#_ENREF_47). Collectively, these conserved interactions define the fundamental pharmacophoric features governing ligand recognition and binding within the Beta2AR.

- **DRD2** Key interactions: (1) Hydrophobic contacts to Trp386; (2) Salt bridge (protein negative) to Asp114.

The D₂ dopamine receptor (DRD2) is a primary therapeutic target for both antipsychotic drugs and Parkinson’s disease treatments. In this study, we employed the inactive-state structure of DRD2 bound to the antagonist spiperone.

Similar to the 5-HT2A receptor discussed earlier, as an aminergic GPCR, DRD2 features a highly conserved salt bridge between the ligand’s protonated amine group and Asp^3.32^ (corresponding to Asp114 in DRD2) which is a hallmark interaction essential for ligand recognition and binding[51-54](#_ENREF_51). In addition, consistent with its antagonistic nature, hydrophobic or π-π interactions with the toggle switch residue Trp386^6.48^ are also critical, as these contacts help stabilize the receptor in its inactive conformation[51](#_ENREF_51)^,^[52](#_ENREF_52).

## 4.3 Kinases

- **BRAF** Key interaction(s): Hydrogen bonds to Cys532.

BRAF belongs to the RAF kinase family, which initiates signal transduction through the MAPK signaling cascade to regulate cell growth, proliferation, and differentiation[55](#_ENREF_55). Traditional ATP-competitive type I BRAF inhibitors effectively block the MAPK pathway in BRAF V600E mutant cells. However, in wild-type BRAF, these inhibitors can induce conformational changes that promote heterodimerization with CRAF, paradoxically leading to hyperactivation of the MAPK pathway[55](#_ENREF_55)^,^[56](#_ENREF_56). This undesired activation can be mitigated by type II inhibitors, which bind to the kinase in its DFG-out conformation. The structure used in this study corresponds to such a BRAF type II inhibitor complex.

For kinases, hydrogen bonding within the hinge region of the orthosteric pocket is highly conserved; in BRAF, this interaction is primarily mediated by Cys532[55](#_ENREF_55)^,^[56](#_ENREF_56). In the DFG-out conformation of BRAF, in addition to the flipped DFG motif within the activation loop, a key structural feature is the outward movement of the αC-helix, which prevents dimer formation by moving away from the ATP-binding site[55](#_ENREF_55)^,^[56](#_ENREF_56). However, the specific interactions involving the αC-helix are not strictly conserved. For example, some ligands form hydrogen bonds with Glu501[57](#_ENREF_57)^,^[58](#_ENREF_58), whereas others interact with Leu505 instead[59](#_ENREF_59). Therefore, in this study, we focused solely on the hinge-region interactions, while omitting detailed discussion of DFG-specific contacts.

- **BTK** Key interactions: Hydrogen bonds to (1) Met477 as donor and (2) Met477 **or** Glu475 as acceptor.

Bruton’s tyrosine kinase (BTK) plays a central role in the activation of B cells and myeloid cells and is widely regarded as a prominent therapeutic target for autoimmune diseases such as rheumatoid arthritis and systemic lupus erythematosus[60](#_ENREF_60). In this study, we used a co-crystal structure featuring an ATP-competitive, DFG-in, noncovalent orthosteric inhibitor.

As noted earlier, hinge hydrogen bonding is a conserved hallmark of kinase orthosteric pockets. For BTK, small-molecule inhibitors typically engage in a characteristic ‘bidentate’—sometimes even ‘tridentate’—hydrogen-bonding pattern with the hinge. The indispensable interaction is a hydrogen bond donated by the amide nitrogen of Met477, while the companion hydrogen bond(s) generally involve the backbone carbonyl of Met477 and[61](#_ENREF_61)^,^[62](#_ENREF_62)/or[63](#_ENREF_63)^,^[64](#_ENREF_64) Glu475 acting as acceptors. Beyond the hinge region, BTK inhibitors may extend either toward the H3 pocket or the solvent-exposed region, and therefore no additional interactions considered.

- **JAK2** Key interactions: Hydrogen bonds to (1) Leu932 as doner and (2) Leu932 **or** Glu930 as acceptor.

The Janus kinase (JAK) family comprises a group of non-receptor tyrosine kinases including JAK1-3 and TYK2, which play essential roles in numerous intracellular signaling pathways. All family members share a characteristic domain architecture consisting of the catalytically active JAK homology 1 (JH1) domain, an adjacent pseudokinase domain (JH2), and five additional homology domains (JH3-7)[65](#_ENREF_65). In this study, we used a canonical JAK2 structure bound to an orthosteric inhibitor targeting the ATP-binding pocket.

Similar to BTK, JAK2 inhibitors typically form a bidentate hydrogen-bonding pattern with the hinge region of the orthosteric kinase pocket. The principal interaction features a hydrogen bond donated by the backbone amide of Leu932, accompanied by one or more hydrogen bonds in which the backbone carbonyl oxygen of Glu930 and/or Leu932 serves as the acceptor[66](#_ENREF_66)^,^[67](#_ENREF_67).

- **MEK1** Key interactions: (1) Hydrogen bonds to Ser212; (2) Halogen bonds to Val127.

MEK1/2 is a key component of the RAS-RAF-MEK-ERK (or MAPK) signaling cascade, which regulates various cellular processes including proliferation, survival, and differentiation, and is closely associated with the development of multiple human cancers. Most clinically approved MEK1 inhibitors are non-ATP-competitive type III allosteric inhibitors that bind to an allosteric pocket adjacent to the ATP-binding site, forming a ternary MEK-ATP-MEKi complex[68](#_ENREF_68). The structure used in this study belongs to this class.

For such inhibitors, the most critical binding interaction involves a hydrogen bond with the backbone amide of Ser212, which is essential for blocking the RAF-mediated feedback phosphorylation of wild-type MEK[69-72](#_ENREF_69). In addition, many clinically used MEK1 inhibitors contain halogenated aniline groups that penetrate a hydrophobic subpocket and form a halogen bond with Val127, thereby enhancing binding potency [71](#_ENREF_71)^,^[72](#_ENREF_72).

- **PI3K-alpha** Key interactions: (1) Hydrogen bonds to Val851; (2) Hydrophobic contacts to Tyr836 **or** Ile932.

Phosphatidylinositol-3 kinases (PI3Ks) are lipid kinases that phosphorylate the signaling lipid PIP2 to PIP3, and the PI3K pathway is among the most frequently activated pathways in human cancers[73](#_ENREF_73). PI3Kα consists of two subunits: the catalytic subunit p110 and the regulatory subunit p85. Most approved and investigational PI3K inhibitors are ATP-competitive agents targeting the orthosteric pocket of the p110 subunit, whose ATP-binding site is nearly identical across PI3K isoforms[73](#_ENREF_73). The structure used in this study corresponds to this class of inhibitors.

At the hinge region, the hydrogen-bonding interaction mediated by Val851, one of the most conserved residues within PI3K, is essential for ligand binding[73-75](#_ENREF_73). Beyond the hinge interaction, most PI3Kα inhibitors extend toward the distal end of the ATP-binding pocket in the direction of the DFG motif. In the intermediate hydrophobic subpocket, residues such as Tyr836 or Ile932 provide critical hydrophobic contacts, which further enhance ligand binding potency[73](#_ENREF_73)^,^[76](#_ENREF_76)^,^[77](#_ENREF_77).

- **ROCK1** Key interaction(s): Hydrogen bonds to Val156.

As a member of the serine/threonine protein kinase family, Rho-associated protein kinase (ROCK) participates in signaling pathways that regulate multiple cellular functions, including actin cytoskeleton organization and cell adhesion[78](#_ENREF_78). Several ROCK inhibitors have already been approved for clinical use, such as for the treatment of glaucoma and cerebral vasospasm. In this study, we used the ATP-competitive orthosteric inhibitor-bound structure of ROCK1.

For the hinge-binding interactions, the hydrogen bond mediated by Val156 is the most critical feature[79](#_ENREF_79)^,^[80](#_ENREF_80). Although extensive crystallographic evidence indicates that hydrogen bonding between ROCK1 inhibitors and the pocket residue Lys105 is also common, we note that some fragment hits do not engage Lys105. Therefore, only the most conserved interaction pattern was considered in our analysis[79-81](#_ENREF_79).

- **TYK2** Key interactions: Hydrogen bonds to (1) Val690; and (2) Lys642 **or** Glu688.

As noted earlier, TYK2 is a member of the JAK family, and the structure used in this study corresponds to an allosteric inhibitor bound to the TYK2 JH2 pseudokinase domain. The TYK2 JH2 domain adopts a canonical kinase fold and is capable of binding ATP, but substitutions at key catalytic residues render it catalytically inactive, for instance, disruptions of the αC-β3 Glu-Lys salt bridge and substitutions within the DFG and HRD motifs[82-84](#_ENREF_82). Allosteric inhibition of the pseudokinase domain can sterically hinder ATP binding or reduce the flexibility required for catalysis in the active kinase domain, achieving an autoinhibition regulatory effect[82](#_ENREF_82). Consequently, targeting the JH2 domain has emerged as a promising strategy for highly selective TYK2 inhibition.

Here, we broaden the discussion of interactions to include those relevant for selective inhibition, rather than focusing solely on conserved interactions such as ATP-like hinge binding, to better contextualize later analyses of target specificity. Although TYK2 JH2 retains features of a canonical kinase hinge, the key hydrogen-bond interaction involves Val690, the residue homologous to Leu932 in JAK2[84](#_ENREF_84). A defining feature of TYK2 selectivity is the unique alanine pocket created by two structural differences: the replacement of the JAK2 Val at the gatekeeper-adjacent position with a smaller Ala in TYK2, and the substitution of the conserved DFG motif with DPG[85](#_ENREF_85)^,^[86](#_ENREF_86). These changes collectively form a distinctive cavity that is indispensable for selective JH2-domain ligand binding[87](#_ENREF_87)^,^[88](#_ENREF_88). Thus, outside the alanine pocket, we consider hydrogen-bond interactions involving Glu688 in the hinge region and Lys642, whose side chain rotates toward the ligand due to the structural changes mentioned above[86](#_ENREF_86). These interactions are essentially observed in selective TYK2 JH2 inhibitors and were therefore included as key interaction groups in our analysis[87-90](#_ENREF_87).

## 4.4 Nuclear receptors

- **PPAR-alpha** Key interactions: (1) Hydrogen bonds to Ser280; (2) Salt bridge (ligand negative) to His440.

Peroxisome proliferator-activated receptors (PPARs) regulate the expression of genes involved in fatty-acid metabolism and are the primary targets of most triglyceride-lowering fibrate drugs[91](#_ENREF_91). In this study, we used a structure in complex with an antagonist.

The ligand-binding domain (LBD) of PPAR adopts the canonical three-layer α-helical sandwich seen in nuclear receptors, with a Y-shaped ligand-binding pocket embedded within it.[92-94](#_ENREF_92) Among the three arms of this Y-shaped cavity, only one is polar. Within this polar arm, the salt bridge formed with His440 is a key interaction engaged by the carboxylate group of endogenous fatty acids, and Ser280 forms a hydrogen bond that is consistently observed across diverse ligands[91](#_ENREF_91)^,^[92](#_ENREF_92). Because existing ligands may extend into either of the other two hydrophobic arms, interactions in those regions were not considered.

- **RXR-alpha** Key interactions: (1) Hydrogen bonds to Ala327; (2) Salt bridge (ligand negative) to Arg316.

The retinoid X receptor (RXR), a bile-acid-activated nuclear receptor that regulates lipid, cholesterol, and glucose metabolism, is an important therapeutic target for various metabolic diseases. The structure used in this study represents a non-steroidal RXRα agonist complex.

The RXRα LBD contains a highly lipophilic ligand-binding pocket. Most RXR ligands mimic the endogenous ligand retinoic acid by forming a salt bridge with Arg316; moreover, nearly all RXRα ligands possess a carboxylate group that forms a key hydrogen bond with Ala327[95](#_ENREF_95)^,^[96](#_ENREF_96). Beyond these interactions, ligand recognition is largely governed by shape complementarity and hydrophobic contacts, and no other notably conserved residues are observed.

## 4.5 Other targets

- **BCL** Key interactions: Hydrophobic contacts to (1) Phe104; (2) Tyr108 **or** Arg146; (3) Phe153; and (4) Phe112.

B-cell lymphoma 2 (Bcl-2) is a key regulatory target in apoptosis and plays an essential role in lymphoid malignancies as well as certain solid tumors. The Bcl-2 family proteins are broadly classified into anti-apoptotic and pro-apoptotic groups, which can be further divided into distinct subfamilies. They typically contain one or more Bcl-2 homology regions (BH1–BH4) that mediate protein–protein interactions. Among these, the BH3 domain is the critical region through which pro-apoptotic proteins engage anti-apoptotic counterparts[97](#_ENREF_97)^,^[98](#_ENREF_98). For BCL-2 playing an anti-apoptotic role, it binds BH3-only proteins within the Bcl-2 family, thereby preventing their association with pro-apoptotic proteins such as BAX and ultimately blocking apoptosis[99](#_ENREF_99). Accordingly, small molecules that mimic the BH3 motif can antagonize BCL-2 overexpression and restore sensitivity to intrinsic apoptosis, making this an attractive strategy for cancer therapy. The structure used in this study corresponds to the BCL-2 protein bound to such BH3-mimetic inhibitors.

The binding pocket between BCL-2 and BH3 forms a hydrophobic groove characteristic of protein–protein interaction interfaces. Among its features, the P2 and P4 hydrophobic subpockets are particularly crucial for BH3 engagement[99-103](#_ENREF_99). Phe153 resides at the distal end of P2, whereas Phe112 is positioned within the hydrophobic cavity of P2; most reported BCL-2 inhibitors make hydrophobic contacts with these residues. Within the P3 tunnel connecting the P2 and P4 regions, inhibitors typically contact either Tyr108 or Arg146. In the P4 pocket, a canonical hydrophobic contact occurs with Phe104. Through this series of hydrophobic interactions spanning P2 to P4, small-molecule inhibitors effectively mimic the native BH3–BCL-2 interface.

- **BRD4** Key interactions: (1) Hydrogen bonds to Asn140; Hydrophobic contacts to (2) Trp81 **or** Pro82 **or** Phe83; and (3) Leu92 **or** Leu94 **or** Val87 **or** Tyr97.

Bromodomain-containing proteins (BCPs) can specifically recognize and bind acetylated lysine residues on histones and represent an important class of epigenetic post-translational modification readers[104](#_ENREF_104). Among them, the bromodomain and extra-terminal (BET) family is particularly notable, and BET proteins typically contain two N-terminal bromodomains (BDs)[105](#_ENREF_105). BRD4 is one member of the BET family, and its two bromodomains (BD1 and BD2) are highly conserved. Both domains recognize and bind acetylated lysine residues, yet they display distinct substrate preferences toward acetylated sites on histone H3 and H4 tails[106](#_ENREF_106). In this study, we focused on structural data of BD1 in complex with well-characterized inhibitors and did not further consider BD isoform selectivity. In addition, a canonical apo BD1 structure was included in our benchmark.

The BRD4 bromodomain forms a hydrophobic pocket composed of four antiparallel α-helices and the connecting loop regions[104](#_ENREF_104). Structural studies of histone binding have shown that the substrate engages in a critical interaction with the conserved Asn140, which is also a defining feature across inhibitor-bound complexes[105](#_ENREF_105)^,^[107](#_ENREF_107). Another characteristic interaction involves a structured water network mediated by the conserved Tyr97[104](#_ENREF_104)^,^[108](#_ENREF_108). Though due to technical limitations, this water network was not considered in the present study. Beyond hydrogen bonding, a unique structural element of the BD is the WPF shelf, formed by Trp81, Pro82, and Phe83; acetyl-lysine mimetic ligands frequently extend hydrophobic substituents toward this region to establish key van-der-Waals contacts[109-111](#_ENREF_109). Opposite to the WPF shelf is the ZA channel, formed by the αZ and αA helices, which also serves as a common ligand-binding region. Within this channel, inhibitors often form extensive hydrophobic contacts with residues such as Leu92, Leu94, Val87, or Tyr97[104](#_ENREF_104)^,^[110](#_ENREF_110).

# 5. Introduction to Models or Methods for Evaluation

Our selection criteria included models published in peer-reviewed venues within the two years prior to June 2025, for which publicly available checkpoints were provided.

**Non-3D Models** DeepBlock formulates molecular generation as an autoregressive assembly of predefined chemical fragments. The model conditions on protein sequence embeddings and iteratively samples building blocks using both simulated annealing and Bayesian optimization.

DRAGONFLY applies a graph transformer neural network to model protein–ligand interactions or as interactome data, with a long-short-term memory (LSTM) based chemical language model for token-wise generation. It can encode 3D-protein embeddings and 2D-ligands to one-dimensional feature vector to generate molecules autoregressively.

SimpleSBDD adopts an economical performance-aware optimization setting. It first samples molecular graphs without atom labels, optimizes them using predicted affinity scores, and then assigns atom types and 3D conformations to construct complete ligands.

TamGen is a GPT-like chemical language model with two modules to encode target protein in structure and sequence. It generates molecules as token sequences in an autoregressive manner, followed by refinement steps to ensure chemical validity and compatibility with protein context.

**3D *in-situ* Models** DiffSBDD applies an SE(3)-equivariant graph diffusion process that jointly denoises atomic identities and 3D coordinates. The model generates ligands non-autoregressively in a pocket-centered coordinate frame, ensuring invariance to rotation and translation.

DrugFlow integrates flow matching with a discrete Markov bridge to map base distributions into molecular structures. The framework samples atomic positions and types conditioned on pocket features.

IPDiff incorporates predefined protein–ligand interaction priors into a 3D diffusion framework. Integrating pocket information to model in both noising and denoising procedure, the model enforces interaction-aware constraints while predicting coordinates and atom types within the binding pocket.

Lingo3DMol combines fragment-level autoregression with coordinate prediction. A chemical language backbone proposes fragments in a format called FSMILES, which are then positioned in three dimensions relative to the binding pocket. Additionally, a non-covalent interaction (NCI) prediction model is also applied to predict the NCI site for generating the first ligand atom.

PocketFlow is an autoregressive flow model operating at the atom level. At each step, it selects a focal atom, predicts a new atom type and position, and assigns bonds using triangular attention and valence constraints. Chemical knowledge rules are applied during sampling, with resampling if constraints are violated.

MolCraft formulates molecular generation as Bayesian Flow Networks. The model parameterizes both atomic placement and molecular geometry in a continuous space, allowing probabilistic sampling consistent with pocket context.

PocketFlow is an autoregressive flow model operating at the atom level. At each step, it selects a focal atom, predicts a new atom type and position, and assigns bonds using triangular attention and valence constraints. Chemical knowledge rules are applied during sampling, with resampling if constraints are violated.

SurfGen uses equivariant graph neural networks to encode protein surface topology and local geometry. Molecules are generated by placing atoms relative to surface features, aiming to reproduce complementary spatial patterns.

TargetDiff extends equivariant diffusion models to jointly model atomic identities and spatial coordinates. It conditions generation on localized pocket environments and iteratively denoises noisy molecular states into valid ligands.

**Optimization-based Methods** DrugFlow-PA extends the DrugFlow framework with preference alignment. Using pairwise comparison data, it adjusts sampling trajectories so that generated molecules are shifted toward distributions consistent with preferred binding or plausibility outcomes.

MolPilot employs a variational lower bound–optimal scheduling strategy to balance discrete and continuous modalities during training. As an optimization extension based on Molcraft, it modifies sampling schedules of flow or diffusion backbones to improve ligand construction.

REINVENT4 is a generative framework based on recurrent and transformer architectures for SMILES or SELFIES strings. It supports reinforcement learning, transfer learning, and curriculum strategies, enabling iterative optimization of generated molecules with respect to user-defined scoring functions. Here we use reinforcement learning with only Vina Score obtained by QuickVina2 as reward.

# References

1. Özçelik, R. & Grisoni, F. The Jungle of Generative Drug Discovery: Traps, Treasures, and Ways Out. *arXiv preprint arXiv:2501.05457* (2024).

2. Xie, Y., Xu, Z., Ma, J. & Mei, Q. HOW MUCH SPACE HAS BEEN EXPLORED? MEASURING THE CHEMICAL SPACE COVERED BY DATABASES AND MACHINE-GENERATED MOLECULES. in *11th International Conference on Learning Representations, ICLR 2023* (2023).

3. Hedges, L.V. Distribution Theory for Glass's Estimator of Effect Size and Related Estimators. *Journal of Educational Statistics* **6**, 107-128 (1981).

4. Leung, S., Bodkin, M., Von Delft, F., Brennan, P. & Morris, G. SuCOS is better than RMSD for evaluating fragment elaboration and docking poses. *ChemRxiv* (2019).

5. Bolcato, G., Heid, E. & Boström, J. On the Value of Using 3D Shape and Electrostatic Similarities in Deep Generative Methods. *Journal of Chemical Information and Modeling* **62**, 1388-1398 (2022).

6. LU Tian, C.F.-W. Comparison of Computational Methods for Atomic Charges. *Acta Phys. -Chim. Sin.* **28**, 1-18 (2012).

7. Lovering, F., Bikker, J. & Humblet, C. Escape from Flatland: Increasing Saturation as an Approach to Improving Clinical Success. *Journal of Medicinal Chemistry* **52**, 6752-6756 (2009).

8. Chalk, S. & McEwen, L. The IUPAC Gold Book. *Chemistry International* **39**, 25-30 (2017).

9. Lipinski, C.A., Lombardo, F., Dominy, B.W. & Feeney, P.J. Experimental and computational approaches to estimate solubility and permeability in drug discovery and development settings. *Advanced Drug Delivery Reviews* **64**, 4-17 (2012).

10. Rogers, D. & Hahn, M. Extended-Connectivity Fingerprints. *Journal of Chemical Information and Modeling* **50**, 742-754 (2010).

11. Bertz, S.H. The first general index of molecular complexity. *Journal of the American Chemical Society* **103**, 3599-3601 (1981).

12. Krzyzanowski, A., Pahl, A., Grigalunas, M. & Waldmann, H. Spacial Score─A Comprehensive Topological Indicator for Small-Molecule Complexity. *Journal of Medicinal Chemistry* **66**, 12739-12750 (2023).

13. Bickerton, G.R., Paolini, G.V., Besnard, J., Muresan, S. & Hopkins, A.L. Quantifying the chemical beauty of drugs. *Nature Chemistry* **4**, 90-98 (2012).

14. Ertl, P. & Schuffenhauer, A. Estimation of synthetic accessibility score of drug-like molecules based on molecular complexity and fragment contributions. *Journal of Cheminformatics* **1**, 8 (2009).

15. Baell, J.B. & Holloway, G.A. New Substructure Filters for Removal of Pan Assay Interference Compounds (PAINS) from Screening Libraries and for Their Exclusion in Bioassays. *Journal of Medicinal Chemistry* **53**, 2719-2740 (2010).

16. Papadatos, G.*, et al.* SureChEMBL: a large-scale, chemically annotated patent document database. *Nucleic Acids Research* **44**, D1220-D1228 (2016).

17. Ertl, P., Rohde, B. & Selzer, P. Fast Calculation of Molecular Polar Surface Area as a Sum of Fragment-Based Contributions and Its Application to the Prediction of Drug Transport Properties. *Journal of Medicinal Chemistry* **43**, 3714-3717 (2000).

18. Hopkins, A.L., Groom, C.R. & Alex, A. Ligand efficiency: a useful metric for lead selection. *Drug Discovery Today* **9**, 430-431 (2004).

19. Preuer, K., Renz, P., Unterthiner, T., Hochreiter, S. & Klambauer, G. Fréchet ChemNet Distance: A Metric for Generative Models for Molecules in Drug Discovery. *Journal of Chemical Information and Modeling* **58**, 1736-1741 (2018).

20. Scheuerer, S.*, et al.* Biological and structural investigation of tetrahydro-β-carboline-based selective HDAC6 inhibitors with improved stability. *European Journal of Medicinal Chemistry* **276**, 116676 (2024).

21. Fischer, F.*, et al.* Deciphering the Therapeutic Potential of Novel Pentyloxyamide-Based Class I, IIb HDAC Inhibitors against Therapy-Resistant Leukemia. *Journal of Medicinal Chemistry* **67**, 21223-21250 (2024).

22. Porter, N.J., Mahendran, A., Breslow, R. & Christianson, D.W. Unusual zinc-binding mode of HDAC6-selective hydroxamate inhibitors. *Proceedings of the National Academy of Sciences* **114**, 13459-13464 (2017).

23. Hai, Y. & Christianson, D.W. Histone deacetylase 6 structure and molecular basis of catalysis and inhibition. *Nature Chemical Biology* **12**, 741-747 (2016).

24. Motlová, L.*, et al.* Comprehensive Mechanistic View of the Hydrolysis of Oxadiazole-Based Inhibitors by Histone Deacetylase 6 (HDAC6). *ACS Chemical Biology* **18**, 1594-1610 (2023).

25. Garcha, H.K.*, et al.* High Efficacy and Drug Synergy of HDAC6-Selective Inhibitor NN-429 in Natural Killer (NK)/T-Cell Lymphoma. *Pharmaceuticals* **15**, 1321 (2022).

26. Esnouf, R.M.*, et al.* Unique features in the structure of the complex between HIV-1 reverse transcriptase and the bis(heteroaryl)piperazine (BHAP) U-90152 explain resistance mutations for this nonnucleoside inhibitor. *Proceedings of the National Academy of Sciences* **94**, 3984-3989 (1997).

27. Yang, Y.*, et al.* Structural basis for potent and broad inhibition of HIV-1 RT by thiophene[3,2-d]pyrimidine non-nucleoside inhibitors. *eLife* **7**, e36340 (2018).

28. Namasivayam, V.*, et al.* The Journey of HIV-1 Non-Nucleoside Reverse Transcriptase Inhibitors (NNRTIs) from Lab to Clinic. *Journal of Medicinal Chemistry* **62**, 4851-4883 (2019).

29. Prener, L.*, et al.* Design and Synthesis of Novel HIV-1 NNRTIs with Bicyclic Cores and with Improved Physicochemical Properties. *Journal of Medicinal Chemistry* **66**, 1761-1777 (2023).

30. Wei, Y., Xiang, H. & Zhang, W. Review of various NAMPT inhibitors for the treatment of cancer. *Frontiers in Pharmacology* **Volume 13 - 2022**(2022).

31. Wang, T.*, et al.* Structure of Nampt/PBEF/visfatin, a mammalian NAD+ biosynthetic enzyme. *Nature Structural & Molecular Biology* **13**, 661-662 (2006).

32. Böhnke, N.*, et al.* A Novel NAMPT Inhibitor-Based Antibody–Drug Conjugate Payload Class for Cancer Therapy. *Bioconjugate Chemistry* **33**, 1210-1221 (2022).

33. Ratia, K.M.*, et al.* Mechanism of Allosteric Modulation of Nicotinamide Phosphoribosyltransferase to Elevate Cellular NAD+. *Biochemistry* **62**, 923-933 (2023).

34. Antonysamy, S.*, et al.* Crystal structure of the human PRMT5:MEP50 complex. *Proceedings of the National Academy of Sciences* **109**, 17960-17965 (2012).

35. Smith, C.R.*, et al.* Fragment optimization and elaboration strategies – the discovery of two lead series of PRMT5/MTA inhibitors from five fragment hits. *RSC Medicinal Chemistry* **13**, 1549-1564 (2022).

36. Mavrakis, K.J.*, et al.* Disordered methionine metabolism in MTAP/CDKN2A-deleted cancers leads to dependence on PRMT5. *Science* **351**, 1208-1213 (2016).

37. Chan-Penebre, E.*, et al.* A selective inhibitor of PRMT5 with in vivo and in vitro potency in MCL models. *Nature Chemical Biology* **11**, 432-437 (2015).

38. Belmontes, B.*, et al.* AMG 193, a Clinical Stage MTA-Cooperative PRMT5 Inhibitor, Drives Antitumor Activity Preclinically and in Patients with MTAP-Deleted Cancers. *Cancer Discovery* **15**, 139-161 (2025).

39. Smith, C.R.*, et al.* Fragment-Based Discovery of MRTX1719, a Synthetic Lethal Inhibitor of the PRMT5•MTA Complex for the Treatment of MTAP-Deleted Cancers. *Journal of Medicinal Chemistry* **65**, 1749-1766 (2022).

40. Smith, J.M.*, et al.* Discovery and In Vivo Efficacy of AZ-PRMT5i-1, a Novel PRMT5 Inhibitor with High MTA Cooperativity. *Journal of Medicinal Chemistry* **67**, 13604-13638 (2024).

41. Cao, D.*, et al.* Structure-based discovery of nonhallucinogenic psychedelic analogs. *Science* **375**, 403-411 (2022).

42. Kimura, K.T.*, et al.* Structures of the 5-HT2A receptor in complex with the antipsychotics risperidone and zotepine. *Nature Structural & Molecular Biology* **26**, 121-128 (2019).

43. Kim, K.*, et al.* Structure of a Hallucinogen-Activated Gq-Coupled 5-HT<sub>2A</sub> Serotonin Receptor. *Cell* **182**, 1574-1588.e1519 (2020).

44. Chen, Z.*, et al.* Structure-based design of a novel third-generation antipsychotic drug lead with potential antidepressant properties. *Nature Neuroscience* **25**, 39-49 (2022).

45. Oguma, T.*, et al.* Dual 5-HT2A and 5-HT2C Receptor Inverse Agonist That Affords In Vivo Antipsychotic Efficacy with Minimal hERG Inhibition for the Treatment of Dementia-Related Psychosis. *Journal of Medicinal Chemistry* **67**, 14478-14492 (2024).

46. Chen, Z.*, et al.* Flexible scaffold-based cheminformatics approach for polypharmacological drug design. *Cell* **187**, 2194-2208.e2122 (2024).

47. Guo, Q.*, et al.* A method for structure determination of GPCRs in various states. *Nature Chemical Biology* **20**, 74-82 (2024).

48. Rasmussen, S.G.F.*, et al.* Structure of a nanobody-stabilized active state of the β2 adrenoceptor. *Nature* **469**, 175-180 (2011).

49. Yang, F.*, et al.* Different conformational responses of the β2-adrenergic receptor-Gs complex upon binding of the partial agonist salbutamol or the full agonist isoprenaline. *National Science Review* **8**(2020).

50. Zhang, Y.*, et al.* Single-particle cryo-EM structural studies of the β2AR–Gs complex bound with a full agonist formoterol. *Cell Discovery* **6**, 45 (2020).

51. Im, D.*, et al.* Structure of the dopamine D2 receptor in complex with the antipsychotic drug spiperone. *Nature Communications* **11**, 6442 (2020).

52. Wang, S.*, et al.* Structure of the D2 dopamine receptor bound to the atypical antipsychotic drug risperidone. *Nature* **555**, 269-273 (2018).

53. Xu, P.*, et al.* Structural genomics of the human dopamine receptor system. *Cell Research* **33**, 604-616 (2023).

54. Zhuang, Y.*, et al.* Structural insights into the human D1 and D2 dopamine receptor signaling complexes. *Cell* **184**, 931-942.e918 (2021).

55. Gunderwala, A., Cope, N. & Wang, Z. Mechanism and inhibition of BRAF kinase. *Current Opinion in Chemical Biology* **71**, 102205 (2022).

56. Singh, A.K.*, et al.* Challenges and Opportunities in the Crusade of BRAF Inhibitors: From 2002 to 2022. *ACS Omega* **8**, 27819-27844 (2023).

57. Arora, R.*, et al.* Design, synthesis and characterisation of a novel type II B-RAF paradox breaker inhibitor. *European Journal of Medicinal Chemistry* **250**, 115231 (2023).

58. Tkacik, E.*, et al.* Structure and RAF family kinase isoform selectivity of type II RAF inhibitors tovorafenib and naporafenib. *Journal of Biological Chemistry* **299**, 104634 (2023).

59. Zhang, C.*, et al.* RAF inhibitors that evade paradoxical MAPK pathway activation. *Nature* **526**, 583-586 (2015).

60. Ran, F.*, et al.* Review of the development of BTK inhibitors in overcoming the clinical limitations of ibrutinib. *European Journal of Medicinal Chemistry* **229**, 114009 (2022).

61. Gomez, E.B.*, et al.* Preclinical characterization of pirtobrutinib, a highly selective, noncovalent (reversible) BTK inhibitor. *Blood* **142**, 62-72 (2023).

62. Montoya, S.*, et al.* Kinase-impaired BTK mutations are susceptible to clinical-stage BTK and IKZF1/3 degrader NX-2127. *Science* **383**, eadi5798 (2024).

63. Ma, B.*, et al.* Discovery of BIIB068: A Selective, Potent, Reversible Bruton’s Tyrosine Kinase Inhibitor as an Orally Efficacious Agent for Autoimmune Diseases. *Journal of Medicinal Chemistry* **63**, 12526-12541 (2020).

64. Crawford, J.J.*, et al.* Discovery of GDC-0853: A Potent, Selective, and Noncovalent Bruton’s Tyrosine Kinase Inhibitor in Early Clinical Development. *Journal of Medicinal Chemistry* **61**, 2227-2245 (2018).

65. Lucet, I.S.*, et al.* The structural basis of Janus kinase 2 inhibition by a potent and specific pan-Janus kinase inhibitor. *Blood* **107**, 176-183 (2006).

66. Davis, R.R.*, et al.* Structural Insights into JAK2 Inhibition by Ruxolitinib, Fedratinib, and Derivatives Thereof. *Journal of Medicinal Chemistry* **64**, 2228-2241 (2021).

67. Miao, Y.*, et al.* Functional and Structural Characterization of Clinical-Stage Janus Kinase 2 Inhibitors Identifies Determinants for Drug Selectivity. *Journal of Medicinal Chemistry* **67**, 10012-10024 (2024).

68. Ram, T.*, et al.* MEK inhibitors in cancer treatment: structural insights, regulation, recent advances and future perspectives. *RSC Medicinal Chemistry* **14**, 1837-1857 (2023).

69. Gonzalez-Del Pino, G.L.*, et al.* Allosteric MEK inhibitors act on BRAF/MEK complexes to block MEK activation. *Proceedings of the National Academy of Sciences* **118**, e2107207118 (2021).

70. Lito, P.*, et al.* Disruption of CRAF-Mediated MEK Activation Is Required for Effective MEK Inhibition in KRAS Mutant Tumors. *Cancer Cell* **25**, 697-710 (2014).

71. Robarge, K.D.*, et al.* Structure based design of novel 6,5 heterobicyclic mitogen-activated protein kinase kinase (MEK) inhibitors leading to the discovery of imidazo[1,5-a] pyrazine G-479. *Bioorganic & Medicinal Chemistry Letters* **24**, 4714-4723 (2014).

72. Ohren, J.F.*, et al.* Structures of human MAP kinase kinase 1 (MEK1) and MEK2 describe novel noncompetitive kinase inhibition. *Nature Structural & Molecular Biology* **11**, 1192-1197 (2004).

73. Zhang, M., Jang, H. & Nussinov, R. PI3K inhibitors: review and new strategies. *Chemical Science* **11**, 5855-5865 (2020).

74. Liu, X.*, et al.* Cryo-EM structures of PI3Kα reveal conformational changes during inhibition and activation. *Proceedings of the National Academy of Sciences* **118**, e2109327118 (2021).

75. Garces, A.E. & Stocks, M.J. Class 1 PI3K Clinical Candidates and Recent Inhibitor Design Strategies: A Medicinal Chemistry Perspective. *Journal of Medicinal Chemistry* **62**, 4815-4850 (2019).

76. Yang, X.*, et al.* New Insights into PI3K Inhibitor Design using X-ray Structures of PI3Kα Complexed with a Potent Lead Compound. *Scientific Reports* **7**, 14572 (2017).

77. Zhou, Q.*, et al.* Structural insights into the interaction of three Y-shaped ligands with PI3Kα. *Proceedings of the National Academy of Sciences* **120**, e2304071120 (2023).

78. Riento, K. & Ridley, A.J. ROCKs: multifunctional kinases in cell behaviour. *Nature Reviews Molecular Cell Biology* **4**, 446-456 (2003).

79. Green, J.*, et al.* Design, Synthesis, and Structure–Activity Relationships of Pyridine-Based Rho Kinase (ROCK) Inhibitors. *Journal of Medicinal Chemistry* **58**, 5028-5037 (2015).

80. Hobson, A.D.*, et al.* Identification of Selective Dual ROCK1 and ROCK2 Inhibitors Using Structure-Based Drug Design. *Journal of Medicinal Chemistry* **61**, 11074-11100 (2018).

81. Beroza, P.*, et al.* Chemical space docking enables large-scale structure-based virtual screening to discover ROCK1 kinase inhibitors. *Nature Communications* **13**, 6447 (2022).

82. Lupardus, P.J.*, et al.* Structure of the pseudokinase–kinase domains from protein kinase TYK2 reveals a mechanism for Janus kinase (JAK) autoinhibition. *Proceedings of the National Academy of Sciences* **111**, 8025-8030 (2014).

83. Jensen, L.T., Attfield, K.E., Feldmann, M. & Fugger, L. Allosteric TYK2 inhibition: redefining autoimmune disease therapy beyond JAK1-3 inhibitors. *eBioMedicine* **97**, 104840 (2023).

84. Min, X.*, et al.* Structural and Functional Characterization of the JH2 Pseudokinase Domain of JAK Family Tyrosine Kinase 2 (TYK2)*. *Journal of Biological Chemistry* **290**, 27261-27270 (2015).

85. Liu, C.*, et al.* Identification of Imidazo[1,2-b]pyridazine Derivatives as Potent, Selective, and Orally Active Tyk2 JH2 Inhibitors. *ACS Medicinal Chemistry Letters* **10**, 383-388 (2019).

86. Moslin, R.*, et al.* Identification of imidazo[1,2-b]pyridazine TYK2 pseudokinase ligands as potent and selective allosteric inhibitors of TYK2 signalling. *MedChemComm* **8**, 700-712 (2017).

87. Moslin, R.*, et al.* Identification of N-Methyl Nicotinamide and N-Methyl Pyridazine-3-Carboxamide Pseudokinase Domain Ligands as Highly Selective Allosteric Inhibitors of Tyrosine Kinase 2 (TYK2). *Journal of Medicinal Chemistry* **62**, 8953-8972 (2019).

88. Wrobleski, S.T.*, et al.* Highly Selective Inhibition of Tyrosine Kinase 2 (TYK2) for the Treatment of Autoimmune Diseases: Discovery of the Allosteric Inhibitor BMS-986165. *Journal of Medicinal Chemistry* **62**, 8973-8995 (2019).

89. Breinlinger, E.*, et al.* Targeting the Tyrosine Kinase 2 (TYK2) Pseudokinase Domain: Discovery of the Selective TYK2 Inhibitor ABBV-712. *Journal of Medicinal Chemistry* **66**, 14335-14356 (2023).

90. Leit, S.*, et al.* Discovery of a Potent and Selective Tyrosine Kinase 2 Inhibitor: TAK-279. *Journal of Medicinal Chemistry* **66**, 10473-10496 (2023).

91. Kamata, S.*, et al.* PPARα Ligand-Binding Domain Structures with Endogenous Fatty Acids and Fibrates. *iScience* **23**, 101727 (2020).

92. Cronet, P.*, et al.* Structure of the PPARα and -γ Ligand Binding Domain in Complex with AZ 242; Ligand Selectivity and Agonist Activation in the PPAR Family. *Structure* **9**, 699-706 (2001).

93. dos Santos, J.C.*, et al.* Different binding and recognition modes of GL479, a dual agonist of Peroxisome Proliferator-Activated Receptor α/γ. *Journal of Structural Biology* **191**, 332-340 (2015).

94. Kuwabara, N.*, et al.* Peroxisome Proliferator-Activated Receptors (PPARs) Have Multiple Binding Points That Accommodate Ligands in Various Conformations: Phenylpropanoic Acid-Type PPAR Ligands Bind to PPAR in Different Conformations, Depending on the Subtype. *Journal of Medicinal Chemistry* **55**, 893-902 (2012).

95. Schierle, S. & Merk, D. Therapeutic modulation of retinoid X receptors – SAR and therapeutic potential of RXR ligands and recent patents. *Expert Opinion on Therapeutic Patents* **29**, 605-621 (2019).

96. de Almeida, N.R. & Conda-Sheridan, M. A review of the molecular design and biological activities of RXR agonists. *Medicinal Research Reviews* **39**, 1372-1397 (2019).

97. Youle, R.J. & Strasser, A. The BCL-2 protein family: opposing activities that mediate cell death. *Nature Reviews Molecular Cell Biology* **9**, 47-59 (2008).

98. Kluck, R.M., Bossy-Wetzel, E., Green, D.R. & Newmeyer, D.D. The Release of Cytochrome c from Mitochondria: A Primary Site for Bcl-2 Regulation of Apoptosis. *Science* **275**, 1132-1136 (1997).

99. Ashkenazi, A., Fairbrother, W.J., Leverson, J.D. & Souers, A.J. From basic apoptosis discoveries to advanced selective BCL-2 family inhibitors. *Nature Reviews Drug Discovery* **16**, 273-284 (2017).

100. Guo, Y.*, et al.* Discovery of the Clinical Candidate Sonrotoclax (BGB-11417), a Highly Potent and Selective Inhibitor for Both WT and G101V Mutant Bcl-2. *Journal of Medicinal Chemistry* **67**, 7836-7858 (2024).

101. Oltersdorf, T.*, et al.* An inhibitor of Bcl-2 family proteins induces regression of solid tumours. *Nature* **435**, 677-681 (2005).

102. Souers, A.J.*, et al.* ABT-199, a potent and selective BCL-2 inhibitor, achieves antitumor activity while sparing platelets. *Nature Medicine* **19**, 202-208 (2013).

103. Liu, J.*, et al.* Sonrotoclax overcomes BCL2 G101V mutation–induced venetoclax resistance in preclinical models of hematologic malignancy. *Blood* **143**, 1825-1836 (2024).

104. Liu, Z.*, et al.* Drug Discovery Targeting Bromodomain-Containing Protein 4. *Journal of Medicinal Chemistry* **60**, 4533-4558 (2017).

105. Schwalm, M.P. & Knapp, S. BET bromodomain inhibitors. *Current Opinion in Chemical Biology* **68**, 102148 (2022).

106. Fu, L.L.*, et al.* Inhibition of BET bromodomains as a therapeutic strategy for cancer drug discovery. *Oncotarget* **6**, 5501-5516 (2015).

107. Ma, Z.*, et al.* Orally Bioavailable BRD4 BD1 Inhibitor ZL0516 Effectively Suppresses Colonic Inflammation in Animal Models of Inflammatory Bowel Disease. *ACS Pharmacology & Translational Science* **8**, 1152-1167 (2025).

108. Andrews, F.H.*, et al.* Dual-activity PI3K–BRD4 inhibitor for the orthogonal inhibition of MYC to block tumor growth and metastasis. *Proceedings of the National Academy of Sciences* **114**, E1072-E1080 (2017).

109. Bradley, E.*, et al.* Structure-Guided Design of a Domain-Selective Bromodomain and Extra Terminal N-Terminal Bromodomain Chemical Probe. *Journal of Medicinal Chemistry* **66**, 15728-15749 (2023).

110. Kim, J.-H.*, et al.* Crystal structure of [1,2,4]triazolo[4,3-b]pyridazine derivatives as BRD4 bromodomain inhibitors and structure–activity relationship study. *Scientific Reports* **13**, 10805 (2023).

111. Watson, R.J.*, et al.* GSK789: A Selective Inhibitor of the First Bromodomains (BD1) of the Bromo and Extra Terminal Domain (BET) Proteins. *Journal of Medicinal Chemistry* **63**, 9045-9069 (2020).
